# Dupilumab Improves Histopathologic Features in Patients With Eosinophilic Esophagitis: LIBERTY EoE TREET Study Results

**DOI:** 10.1016/j.gastha.2025.100646

**Published:** 2025-02-24

**Authors:** Margaret H. Collins, Marc E. Rothenberg, Evan S. Dellon, Albert J. Bredenoord, Ikuo Hirano, John Leung, Xian Sun, Lila Glotfelty, Arsalan Shabbir

**Affiliations:** 1 Division of Pathology and Laboratory Medicine, Cincinnati Hospital Medical Center, Cincinnati, Ohio; 2 Division of Allergy and Immunology, Department of Pediatrics, Cincinnati Children’s Hospital Medical Center and University of Cincinnati College of Medicine, Cincinnati, Ohio; 3 Center for Esophageal Diseases and Swallowing, University of North Carolina School of Medicine, Chapel Hill, North Carolina; 4 Amsterdam University Medical Center, Amsterdam, The Netherlands; 5 Northwestern University Feinberg School of Medicine, Chicago, Illinois; 6 Boston Specialists/Boston Food Allergy Center, Boston, Massachusetts; 7 Regeneron Pharmaceuticals Inc., Tarrytown, New York; 8 Sanofi, Bridgewater, New Jersey

**Keywords:** Eosinophilic Esophagitis, Dupilumab, Histology, Pathology, Endoscopy

## Abstract

**Background and Aims:**

This post hoc analysis assessed the effect of dupilumab on the individual components of the Eosinophilic Esophagitis Histology Scoring System (EoEHSS) and the relationship between histopathologic and endoscopic measures in the LIBERTY eosinophilic esophagitis (EoE) TREET population.

**Methods:**

The analysis included patients who received dupilumab 300 mg weekly (qw) or placebo for 24 weeks (Parts A and B). Eligible patients who completed Parts A or B entered Part C and received dupilumab 300 mg qw for 28 weeks (week 52). Changes from baseline to week 24 and week 52 in the EoEHSS grade/stage components were assessed. Associations between histopathologic, symptomatic, and endoscopic features were evaluated using Spearman correlation.

**Results:**

At week 24, dupilumab improved most EoEHSS grade/stage component scores vs placebo. These improvements were sustained at week 52 in patients continuously on dupilumab; patients who switched from placebo to dupilumab at week 24 improved EoEHSS grade/stage component scores at week 52 to levels similar to those observed in patients continuously on dupilumab. Dupilumab also increased the proportion of patients in remission at week 24 vs placebo, with further increases at week 52, as assessed by the EoE histology remission score. EoEHSS grade and stage total scores correlated strongly with peak eosinophil count (PEC), moderately to strongly with Endoscopic Reference Score, and weakly with Dysphagia Symptom Questionnaire score. Several EoEHSS grade/stage component scores correlated strongly with PEC but not with Dysphagia Symptom Questionnaire scores.

**Conclusion:**

Dupilumab 300 mg qw improved histopathologic measures of EoE beyond PEC at week 24, with improvements sustained to week 52 (Video 1).

## Graphical abstract

**Figure:**
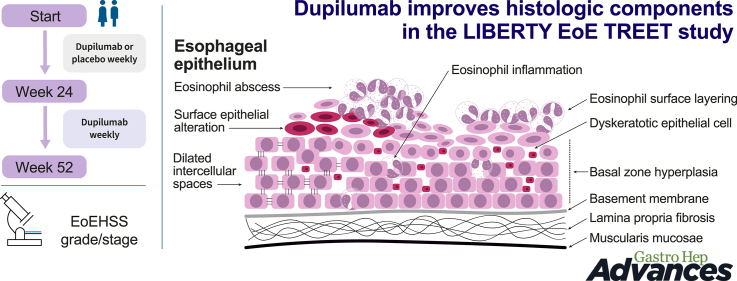

## Introduction

Eosinophilic esophagitis (EoE) is a chronic progressive type 2 immune-mediated inflammatory disease of the esophagus that is increasing in incidence and prevalence.^1^ EoE has a substantial negative impact on the quality of life of affected patients.^1^, ^2^, ^3^, ^4^, ^5^ Patients with EoE present with esophageal inflammation and remodeling partly due to eosinophil infiltration,^6^ and have symptoms such as dysphagia and/or food impaction.^7^ Untreated EoE or treatment failure can lead to esophageal fibrosis and other forms of remodeling, further increasing the risk of stricture and food impaction.^7^ EoE diagnosis relies on symptoms of esophageal dysfunction^6^ and an eosinophil density higher than 15 eosinophils per high-power field (eos/hpf) in esophageal biopsies. However, eosinophil density alone does not always correlate with the patient’s symptoms; previous studies showed improvement in eosinophil infiltration without a corresponding improvement in clinical symptoms.^8^, ^9^, ^10^ Additional histologic changes, such as basal zone hyperplasia (BZH), dilated intercellular spaces (DIS), and thickened lamina propria fibers, are commonly seen in esophageal biopsies displaying the threshold level of eosinophil inflammation (EI) required for EoE diagnosis.^11^ A scoring system that is inclusive of pathologic features beyond eosinophil infiltration could provide a more comprehensive assessment of the disease’s impact and aid in measuring the efficacy of treatments. This prompted the development and validation of the EoE Histology Scoring System (EoEHSS), which allows for a wide-range assessment of tissue inflammatory and architectural changes including epithelial and proliferative changes in esophageal biopsies. This scoring system has facilitated the assessment of the histopathologic severity (grade score) and extent (stage score) of the disease.^11^ The EoEHSS includes features both related to EI (eosinophil density, eosinophil abscesses [EA], eosinophil surface layering [ESL], and surface epithelial alteration) and excluding eosinophils (BZH, DIS, dyskeratotic epithelial cells [DEC], and lamina propria fibrosis [LPF]) in their definition.^11^ Although the threshold level of EI for EoE diagnosis (ie, 15 eos/hpf) is suitable for clinical and research purposes, clinical remission is not clearly defined, in part because of the observed reduction in EI without symptom reduction.^8^, ^9^, ^10^ A proposed histopathology remission score, the EoE histopathology remission score derived from the EoEHSS,^11^^,^^12^ correlated with reduced symptoms and molecular improvements in a single-institution study of children with EoE.

Dupilumab, a fully human monoclonal antibody, blocks the shared receptor component for interleukin (IL)-4/IL-13, key and central drivers of type 2 inflammation in multiple diseases including EoE.^13^, ^14^, ^15^ LIBERTY EoE TREET was a 3-part, double-blind, placebo-controlled, phase 3 study that demonstrated improvements in histologic, symptomatic, endoscopic, and molecular aspects of the disease in adults and adolescents with EoE treated with dupilumab up to 52 weeks.^16^^,^^17^ Weekly (qw) dupilumab treatment vs placebo improved the proportion of patients with peak eosinophil count ≤6 eos/hpf and <15 eos/hpf at week 24. Notably, in Part B of the study, 100% of patients continuously on dupilumab reached remission (defined as <15 eos/hpf) after 52 weeks of treatment. Dupilumab treatment also improved endoscopic features as measured by the EoE Endoscopic Reference Score (EREFS) total score, symptoms of dysphagia as assessed by the Dysphagia Symptom Questionnaire (DSQ) score, and EoEHSS stage and grade total scores at week 24, with further improvement to week 52.^16^^,^^17^

The objective of this study was to conduct a comprehensive analysis of the effect of dupilumab on esophageal histopathology, including the individual components of the EoEHSS and the EoEHSS remission score, and the relationship between those features and endoscopic and dysphagia scores in the LIBERTY EoE TREET population.

## Materials and Methods

### Study Design

This is an analysis of data collected during the 3-part LIBERTY EoE TREET study (NCT03633617), details of which have been published separately.^16^^,^^17^ Briefly, patients ≥12 years of age with EoE were eligible (EoE diagnosis confirmed with peak eosinophil count ≥15 eos/hpf despite 8 weeks of high-dose proton pump inhibitor [PPI] treatment and a DSQ score of ≥10 at randomization). In Part A, patients were randomized 1:1 to subcutaneous placebo or dupilumab 300 mg qw, and in Part B, 1:1:1 to subcutaneous placebo or dupilumab 300 mg qw or dupilumab 300 mg every 2 weeks (q2w). Part C was a 28-week extended active treatment period for eligible patients who completed Part A or B. In Part A–C, patients from Part A received dupilumab 300 mg qw (including patients who previously received placebo), and in Part B–C, patients from Part B who received dupilumab continued the same regimen, while those who received placebo were switched to either dupilumab 300 mg qw or dupilumab 300 mg q2w.

The current analysis included patients treated with dupilumab 300 mg qw (the approved EoE regimen) or placebo for up to 52 weeks, unless otherwise stated.

All authors had access to the study data and have reviewed and approved the final manuscript.

### Endpoints and Assessments

#### Histopathology evaluation

Esophageal biopsies were collected endoscopically and assessed for peak eosinophil count and, in addition to EI, scored for abnormalities using the EoEHSS. The EoEHSS grade and stage scores evaluate 8 pathologic features: EI (based on peak eosinophil count), BZH, EA, ESL, DIS, surface epithelial alteration, DEC, and LPF. When assessing the grade and stage component scores, the review pathologist (M.H.C.) was blinded to the study treatment and followed the definitions of each feature as shown in Table A1. Of note, the peak eosinophil count was expressed as a single discrete number following careful evaluation of all biopsy pieces in 6 different levels displayed on 1 slide; an approximation or a range of numbers is not suitable for evaluating response to therapy in clinical evaluations or therapy trials. Eosinophils in the most inflamed area were counted. In biopsies that were markedly, diffusely, and evenly inflamed, numerous areas were counted to estimate the most accurate peak eosinophil count. Biopsies were initially surveyed at medium (200×) power because eosinophils may be inapparent at lower power magnification in biopsies that are not intensely inflamed; the number of eosinophils was then counted at high (400×, 0.3 mm^2^) power. The stage score for EI was based on the amount of tissue in the biopsy displaying ≥15 eos/hpf. The stage score was not generated by counting the number of eosinophils in each high-power field but was computed during the survey of each biopsy piece in each level. The most time-consuming portion of the process of evaluating esophageal biopsies was ascertaining the true peak eosinophil count. The stage score for EI, and the grade and stage scores for the other features, were most often assigned during the process of obtaining the peak eosinophil count. Severity (grade) and extent (stage) of abnormalities were scored using a 4-point scale (0–3 [normal–maximum change]).^11^ The grade and stage score for each esophageal site was calculated as the sum of the score of each feature divided by the maximum possible score for that biopsy and ranged from 0 to 1. For instance, if all 8 features were evaluated and scored, the maximum total score would be 24. If all 8 features could not be scored, the maximum total score was reduced by 3 for each unevaluable feature. The most common feature that could not be evaluated was lamina propria, which was either missing or unevaluable due to handling/crush artifact; in cases where the lamina propria could not be evaluated, the total possible score was 21 (24 minus 3); the total score in such cases was the observed score for the remaining 7 features divided by 21. The EoEHSS grade and stage total scores were reported as the sum of the mean scores of 3 esophageal sites (proximal, mid, and distal) and ranged from 0 to 3; the component scores were derived from the nonmissing feature scores of the 3 regions and expressed as the sum of the mean scores of 3 esophageal sites and ranged from 0 to 3. Absolute changes in EoEHSS grade and stage total scores, as well as scores for each histologic component, were assessed from baseline to weeks 24 and 52. Absolute changes in EoEHSS grade and stage total score were also assessed for each esophageal site (proximal, mid, and distal). The EoEHSS components were also divided into 2 subscores: inflammatory (including eosinophil density, EA, ESL, and surface epithelial alteration) and architectural (including BZH, DIS, DEC, and LPF).^11^

The EoEHSS remission score, which may be useful to inform clinical care and interstudy comparisons,^12^ is based on the EoEHSS grade and stage scores and the peak eosinophil count. That is, a patient is in EoEHSS remission when both their EoEHSS grade and stage scores are ≤3 (the sum of component scores [range 0–24]) and their peak eosinophil count is <15 eos/hpf. The proportion of patients in remission according to the EoEHSS remission score was assessed at weeks 24 and 52.

The proportions of patients with peak eosinophil count ≤1, ≤6, and <15 eos/hpf and absolute change in peak eosinophil count were assessed for each esophageal site (proximal, mid, and distal).

#### Endoscopy evaluation

EREFS is a validated scoring system^18^ designed to assess endoscopic features of EoE including edema (absent/present [score 0/1]), rings (absent/mild/moderate/severe [0–3]), exudates (absent/mild/severe [0–2]), furrows (absent/mild/severe [0–2]), and strictures (absent/present [0/1]). Both proximal and distal esophageal regions were scored for each feature (score range 0–9), then summed to obtain the total score (0–18). EREFS can be divided into 2 subscores: inflammatory (edema, exudates, and furrows; score range 0–10) and remodeling (rings and strictures; 0–8).^18^ EREFS was assessed at baseline and weeks 24 and 52, and is included here for comparison with EoEHSS scoring.

#### Symptom (dysphagia) evaluation

The DSQ score was calculated using questions 2 and 3 of the DSQ, a validated questionnaire to assess symptoms of dysphagia (score range 0–84).^19^^,^^20^ Similarly to the EREFS score, DSQ score was assessed at baseline and weeks 24 and 52, and is included here for comparison with EoEHSS scoring.

### Statistical Analyses

For continuous variables, the confidence intervals with *P* values were based on treatment difference (dupilumab vs placebo group for Parts A and B) in the least squares mean changes using an analysis of covariance model with baseline measurement as covariate and treatment, age group (≥12–<18 vs ≥18 years), and PPI use at randomization (yes vs no) strata as fixed factors. For binary variables, *P* values were derived using the Cochran–Mantel–Haenszel test stratified by age group (≥12–<18 vs ≥18 years) and PPI use at randomization (yes vs no). All *P* values should be considered nominal unless otherwise specified.

There were no primary efficacy endpoints for Parts A–C and B–C of the study. All secondary endpoints assessed through week 52 were summarized using descriptive statistics without comparator. No formal statistical hypothesis testing was performed.

The relationships between EoEHSS grade and stage (total, inflammatory, and architectural) scores and peak eosinophil count of 3 esophageal regions (proximal, mid, and distal), as well as with EREFS (total, inflammation, and remodeling) and DSQ scores, were evaluated using Spearman correlation. The associations were assessed as strong (*r* ≥0.5), moderate (≥0.3 – <0.5), and weak (<0.3) according to Cohen’s rule of thumb.

In Parts A and B, for all continuous endpoints, values after first rescue treatment used were imputed by worst observation carried forward. Missing values were imputed by worst observation carried forward if not due to COVID-19 and by multiple imputation if due to COVID-19. For the EoEHSS remission score, patients who received rescue treatment were considered as not in remission. Any component scores missing at week 24 were imputed as not in remission if not due to COVID-19 or by multiple imputation if missing due to COVID-19.

The EoEHSS grade and stage mean component scores were derived as the mean of nonmissing component scores of the 3 esophageal regions (proximal, mid, and distal); absolute change from baseline also required a measurement at baseline of Part A or B.

## Results

### Patients and Baseline Characteristics

In Part A, 42 patients were randomized to dupilumab qw and 39 patients to placebo. In Part B, 80 patients were randomized to dupilumab qw, 81 to dupilumab q2w, and 79 to placebo. In Part C, 40 patients from Part A continued dupilumab qw and 37 switched from placebo to dupilumab qw (Part A–C); from Part B, 74 patients continued dupilumab qw, 79 continued dupilumab q2w, 37 switched from placebo to dupilumab qw, and 37 switched from placebo to dupilumab q2w (Part B–C). Baseline demographics and disease characteristics, including EREFS, EoEHSS grade and stage total scores, and component scores were well balanced across treatment groups (Table A2).

### EoEHSS Grade Total and Individual Component Scores

**Figure 1:**
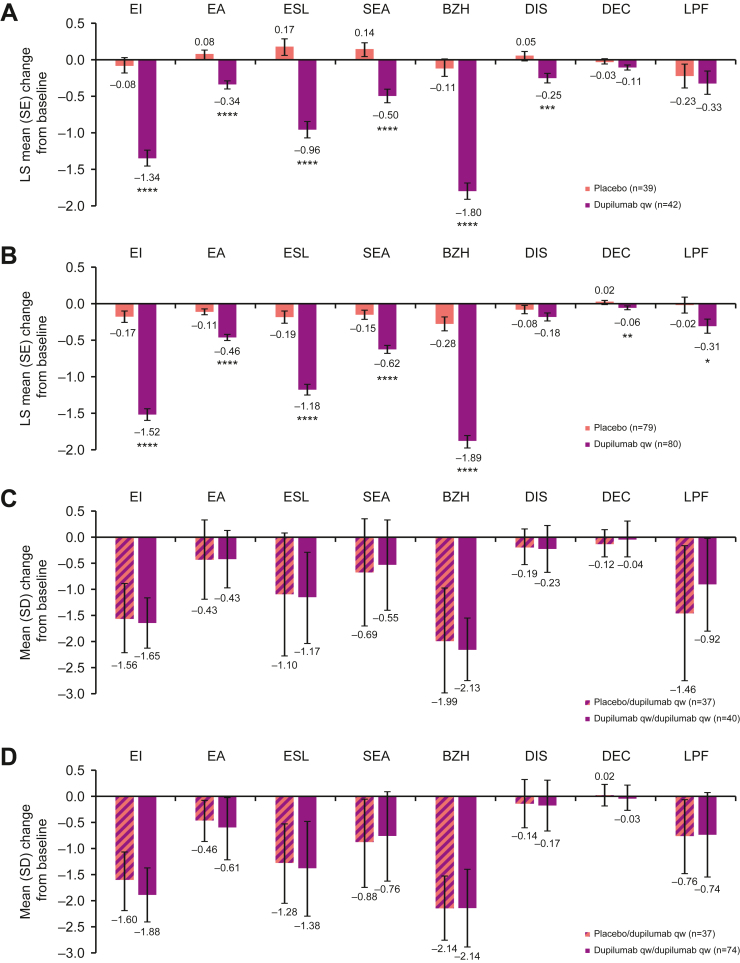
Change from baseline in EoEHSS grade component scores at week 24 (A) Part A and (B) Part B, and week 52 (C) Part A–C (relative to baseline Part A) and (D) Part B–C (relative to baseline Part B). ∗*P* < .05; ∗∗*P* < .01; ∗∗∗*P* < .001; ∗∗∗∗*P* < .0001. The EoEHSS grade and stage mean component scores were derived as the mean of nonmissing component scores of the 3 esophageal regions (proximal, mid, and distal); absolute change from baseline also required a measurement at baseline of Part A or B. LS, least squares; qw, weekly; SD, standard deviation; SE, standard error; SEA, surface epithelial alteration.

All EoEHSS grade component scores numerically improved in the dupilumab-treated group compared with placebo at week 24, except for DEC (though numeric improvement was observed only for Part B) (Figure 1A and B). Dyskeratotic epithelial cell scores were low at baseline in both groups in Part A (dupilumab mean [standard deviation] 0.14 [0.28], placebo 0.21 [0.26]) and Part B (0.13 [0.23], 0.09 [0.22]), which limited the ability to demonstrate improvements. The improvements in grade component scores were sustained at week 52 in patients continuously on dupilumab (Figure 1C and D). For patients switching from placebo to dupilumab at week 24, EoEHSS grade component scores improved at week 52 to levels similar to those observed in patients continuously on dupilumab (Figure 1C and D). Dupilumab also improved EoEHSS grade total score regardless of esophageal biopsy site at week 24 vs placebo, with improvement sustained to week 52 in patients continuously on dupilumab (Figure A1). EoEHSS grade total score also improved regardless of esophageal biopsy site in patients who switched from placebo to dupilumab at week 24 to week 52 (Figure A1B). Of note, the small number of patients with available measurements of LPF did not permit assessment of the impact of the treatments on this component over time (around 25% of lamina propria samples were evaluable at baseline of Parts A and B vs >75% for other components [Table A2], and <50% at baseline of Parts A–C and B–C vs >90% [Table A3]). Similar results were observed with dupilumab q2w treatment (Figures A1 and A2).

### EoEHSS Stage Total and Individual Component Scores

**Figure 2:**
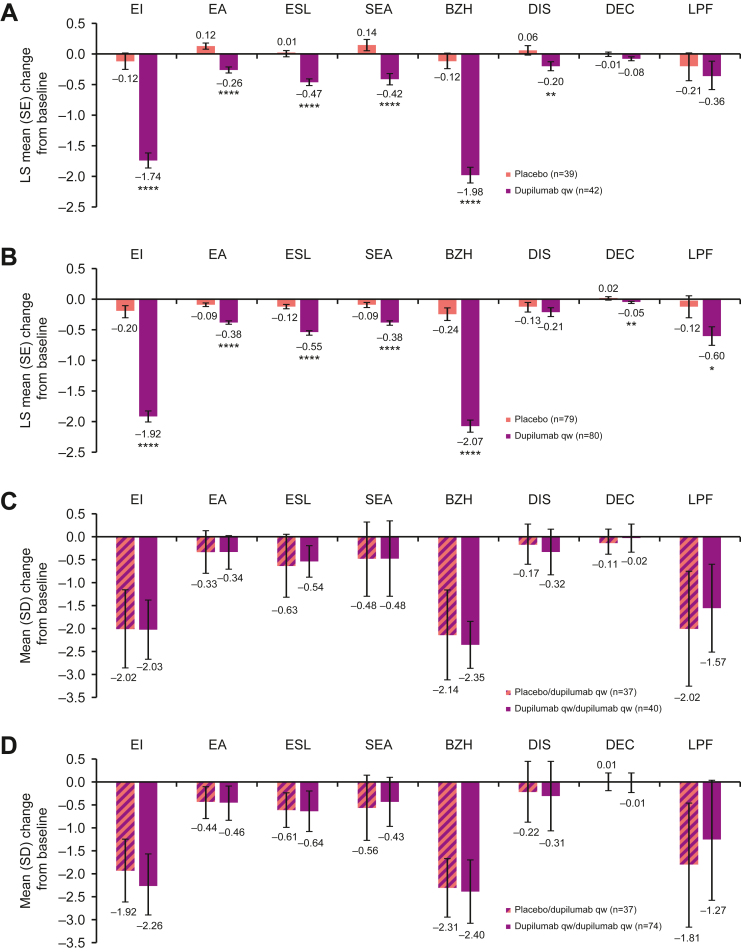
Change from baseline in EoEHSS stage component scores at week 24 (A) Part A and (B) Part B, and week 52 (C) Part A–C (relative to baseline Part A) and (D) Part B–C (relative to baseline Part B). ∗*P* < .05; ∗∗*P* < .01; ∗∗∗∗*P* < .0001. The EoEHSS grade and stage mean component scores were derived as the mean of nonmissing component scores of the 3 esophageal regions (proximal, mid, and distal); absolute change from baseline also required a measurement at baseline of Part A or B. LS, least squares; SD, standard deviation; SE, standard error; SEA, surface epithelial alteration.

All EoEHSS stage component scores improved with dupilumab vs placebo at week 24 (Figure 2A and B), except for dyskeratotic epithelial cell scores (though numeric improvement was observed only for Part B), which, similar to EoEHSS grade scores, were low at baseline in both groups in Part A (dupilumab mean [standard deviation] 0.12 [0.23], placebo 0.20 [0.24]) and Part B (0.11 [0.19], 0.08 [0.19]). The improvements in stage scores observed at week 24 were sustained at week 52 for patients continuously on dupilumab. For patients switching from placebo to dupilumab at week 24, EoEHSS stage component scores improved to levels similar to those observed for patients continuously on dupilumab (Figure 2C and D). Dupilumab also improved EoEHSS stage total score regardless of esophageal biopsy site at week 24 vs placebo, with improvement sustained to week 52 in patients continuously on dupilumab (Figure A3). EoEHSS grade total score also improved regardless of esophageal biopsy site in patients who switched from placebo to dupilumab at week 24 to week 52 (Figure A3B). Of note, similar to the EoEHSS grade score, the small number of patients with available measurements of LPF did not permit assessment of the impact of the treatments on this component over time. Similar results were observed with dupilumab q2w treatment (Figures A3 and A4).

### EoEHSS Remission Score

**Figure 3:**
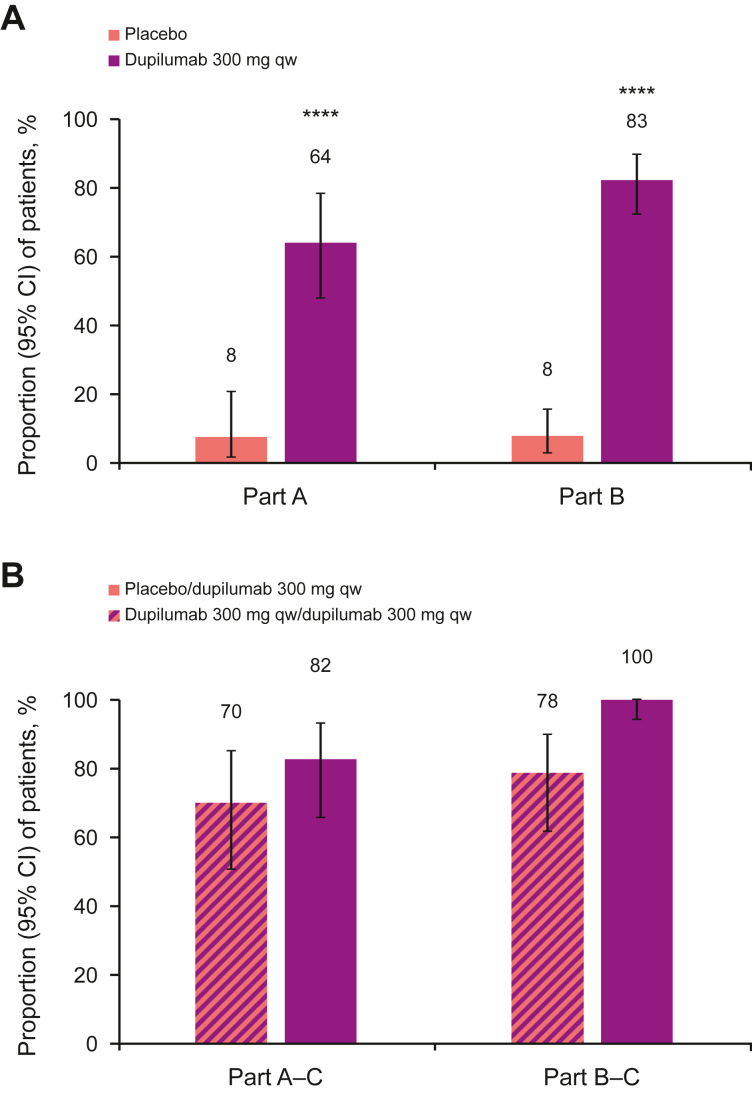
Proportion of patients in remission according to EoEHSS remission score at (A) week 24 and (B) week 52. ∗∗∗∗*P* < .0001. CI, confidence interval.

Dupilumab increased the proportion of patients in histopathologic remission according to the EoEHSS remission score vs placebo at week 24 (64% vs 8% in Part A, 83% vs 8% in Part B) (Figure 3A). The proportion of patients in remission further increased from week 24 to week 52 in patients on continuous dupilumab treatment (82% in Part A–C, 100% in Part B–C); among patients who switched from placebo to dupilumab at week 24, the proportion in remission also increased at week 52 (to 70% and 78%, respectively) (Figure 3B). Similar results, although numerically lower at week 52 (100% for continuous dupilumab qw and 84% for continuous dupilumab q2w in Part B–C), were observed with dupilumab q2w treatment (Figure A5).

### Proportion of Patients with Peak Eosinophil Count by Esophageal Site

Dupilumab improved peak eosinophil counts (Figure A6) and the proportion of patients achieving peak esophageal intraepithelial eosinophil count thresholds of ≤1, ≤6, and <15 eos/hpf (Figures A7–A9) compared with placebo at week 24, regardless of the biopsy site, with improvement sustained to week 52 in patients continuously on dupilumab. Peak eosinophil counts also improved in patients who switched from placebo to dupilumab at week 24 to week 52 (Figures A6–A9). Similar results were observed with dupilumab q2w treatment (Figures A6–A9).

### Example Esophageal Biopsies at Baseline, Week 24, and Week 52

**Figure 4:**
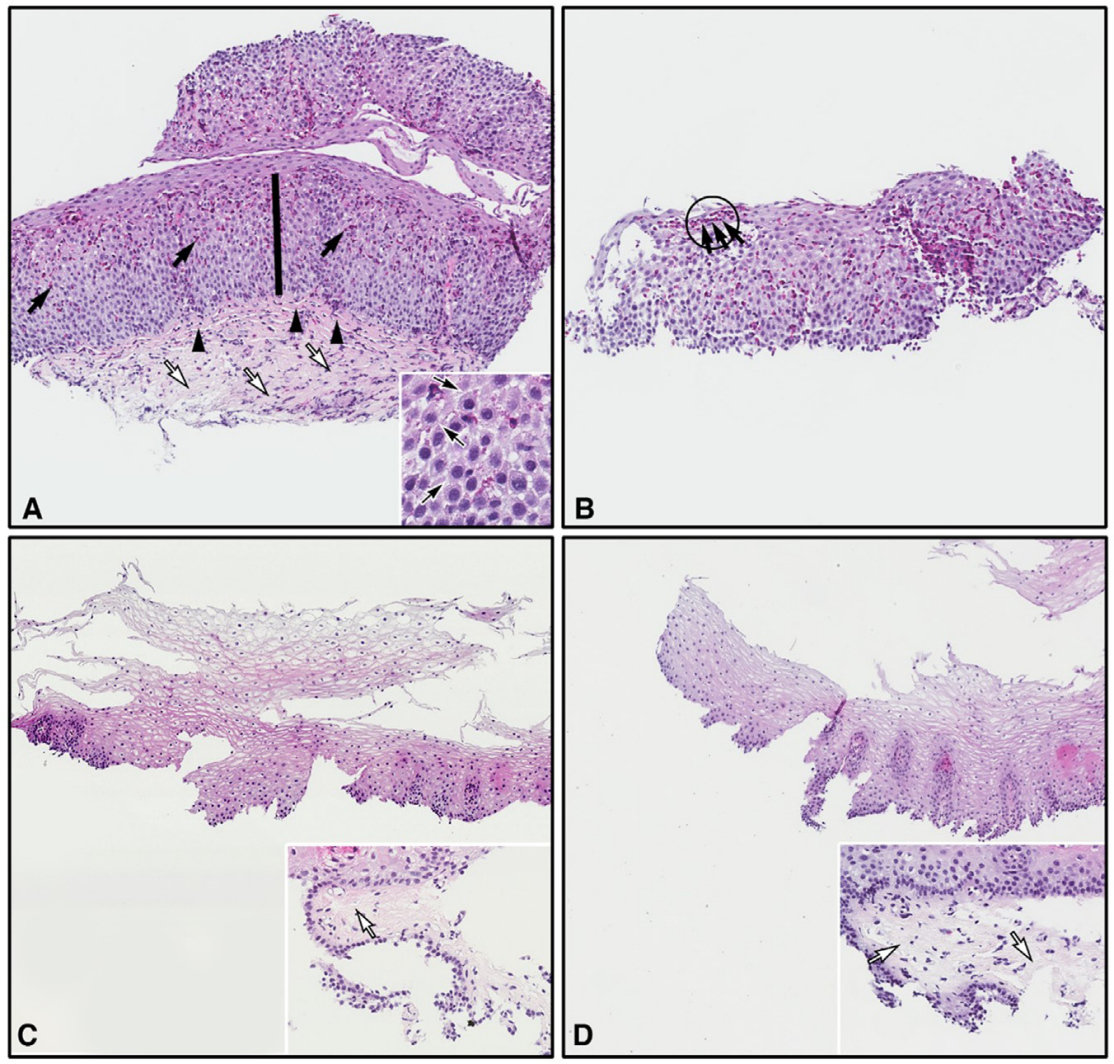
Distal esophageal biopsies from a patient treated with dupilumab 300 mg weekly in part B of the study. (A) Biopsy at baseline showing intraepithelial eosinophils (black arrows); marked basal zone hyperplasia (bar); thickening of the subepithelial fibers in the lamina propria (arrowheads) compared with normal fibers in the deeper lamina propria (white arrows); and dilated intercellular spaces (inset; arrows). (B) Additional baseline biopsy showing eosinophil surface layering (arrows) in an eosinophil abscess (circle). (C) Week 24 and (D) week 52 biopsies from the same patient showing resolution of the pathologic findings observed at baseline; inset figures show partly detached normal lamina propria with delicate connective tissue fibrils (arrows).

Figure 4 shows illustrative distal esophageal biopsies from a patient treated with dupilumab 300 mg qw in Part B of the study. At baseline (Figure 4A and B), the biopsies showed numerous intraepithelial eosinophils, marked BZH, thickening of the subepithelial fibers in the lamina propria, DIS, and surface layering of eosinophils. After 24 weeks (Figure 4C) and 52 weeks (Figure 4D) of dupilumab treatment, all pathologic abnormalities observed at baseline had resolved.

### Correlations Between EoEHSS Grade and Stage Total Score and Peak Eosinophil Count

The correlations between EoEHSS grade and stage total score and peak esophageal intraepithelial eosinophil count at baseline, week 24, and week 52 are presented in Table 1. EoEHSS grade total score strongly correlated with peak eosinophil count (correlation coefficient range 0.51–0.85 for Parts A and B at baseline and week 24; 0.57–0.94 for Parts A–C and B–C at week 52). The correlations between EoEHSS stage total score and peak eosinophil count were moderate to strong at baseline and at week 24 (0.38–0.84) and week 52 (0.34–0.76).

**Table 1:** Correlation Between EoEHSS Grade/Stage Total and Component Scores and PEC at Week 24 and Week 52

	Part A				Part A–C	
	Placebo (n = 39)		Dupilumab qw (n = 42)		Placebo/dupilumab qw (n = 37)	Dupilumab qw/dupilumab qw (n = 40)
	Baseline	Week 24	Baseline	Week 24	Week 52	Week 52
EoEHSS grade score						
PEC and total score	**0.61∗∗∗∗**	**0.85∗∗∗∗**	**0.51∗∗∗**	**0.83∗∗∗∗**	**0.94∗∗∗∗**	**0.69∗∗∗∗**
PEC and inflammatory subscore	**0.63∗∗∗∗**	**0.78∗∗∗∗**	**0.56∗∗∗∗**	**0.91∗∗∗∗**	**0.94∗∗∗∗**	**0.88∗∗∗∗**
PEC and architectural subscore	*0.46∗∗*	**0.60∗∗∗∗**	0.14	**0.70∗∗∗∗**	**0.67∗∗∗∗**	0.22
PEC and eosinophil inflammation	**0.85∗∗∗∗**	**0.88∗∗∗∗**	**0.74∗∗∗∗**	**0.88∗∗∗∗**	**0.95∗∗∗∗**	**0.90∗∗∗∗**
PEC and eosinophil abscesses	**0.72∗∗∗∗**	**0.74∗∗∗∗**	**0.55∗∗∗∗**	**0.62∗∗∗∗**	**0.52∗∗**	0.19
PEC and eosinophil surface layering	**0.56∗∗∗**	**0.72∗∗∗∗**	**0.56∗∗∗∗**	**0.62∗∗∗∗**	**0.52∗∗**	*0.42∗*
PEC and surface epithelial alteration	*0.32∗*	*0.39∗*	0.08	**0.68∗∗∗∗**	*0.47∗∗*	**0.53∗∗**
PEC and basal zone hyperplasia	*0.45∗∗*	**0.71∗∗∗∗**	*0.34∗*	**0.83∗∗∗∗**	**0.59∗∗∗**	**0.56∗∗∗**
PEC and dilated intercellular spaces	*0.30*	0.14	−0.09	*0.40∗∗*	*0.40∗*	−0.03
PEC and dyskeratotic epithelial cells	0.05	0.05	−0.18	0.10	*0.33*	−0.06
PEC and lamina propria fibrosis	**0.51∗**	*0.48∗*	0.08	*0.37*	N/A	0.03
EoEHSS stage score						
PEC and total score	**0.64∗∗∗∗**	**0.84∗∗∗∗**	**0.52∗∗∗**	**0.63∗∗∗∗**	**0.76∗∗∗∗**	**0.60∗∗∗**
PEC and inflammatory subscore	**0.64∗∗∗∗**	**0.78∗∗∗∗**	**0.60∗∗∗∗**	**0.87∗∗∗∗**	**0.76∗∗∗∗**	**0.71∗∗∗∗**
PEC and architectural subscore	*0.46∗∗*	**0.60∗∗∗∗**	0.06	**0.70∗∗∗∗**	**0.59∗∗∗**	*0.38∗*
PEC and eosinophil inflammation	**0.82∗∗∗∗**	**0.92∗∗∗∗**	**0.68∗∗∗∗**	**0.85∗∗∗∗**	**0.81∗∗∗∗**	**0.67∗∗∗∗**
PEC and eosinophil abscesses	**0.72∗∗∗∗**	**0.72∗∗∗∗**	**0.57∗∗∗∗**	**0.62∗∗∗∗**	**0.52∗∗**	0.19
PEC and eosinophil surface layering	**0.58∗∗∗∗**	**0.73∗∗∗∗**	**0.53∗∗∗**	**0.62∗∗∗∗**	**0.52∗∗**	*0.42∗*
PEC and surface epithelial alteration	0.26	*0.34∗*	0.08	**0.67∗∗∗∗**	*0.47∗∗*	**0.53∗∗∗**
PEC and basal zone hyperplasia	**0.50∗∗**	**0.73∗∗∗∗**	*0.32∗*	**0.83∗∗∗∗**	**0.60∗∗∗**	**0.55∗∗∗**
PEC and dilated intercellular spaces	**0.53∗∗∗**	*0.41∗∗*	−0.12	0.21	0.26	0.20
PEC and dyskeratotic epithelial cells	0.02	0.05	−0.18	0.10	*0.33*	−0.06
PEC and lamina propria fibrosis	**0.58∗∗**	*0.46∗*	0.13	0.29	N/A	0.03

	Part B				Part B–C	
	Placebo (n = 79)		Dupilumab qw (n = 80)		Placebo/dupilumab qw (n = 37)	Dupilumab qw/dupilumab qw (n = 74)
	Baseline	Week 24	Baseline	Week 24	Week 52	Week 52
EoEHSS grade						
PEC and total score	**0.59∗∗∗∗**	**0.64∗∗∗∗**	**0.60∗∗∗∗**	**0.67∗∗∗∗**	**0.57∗∗∗**	**0.61∗∗∗∗**
PEC and inflammatory subscore	**0.62∗∗∗∗**	**0.73∗∗∗∗**	**0.66∗∗∗∗**	**0.83∗∗∗∗**	**0.68∗∗∗∗**	**0.78∗∗∗∗**
PEC and architectural subscore	*0.36∗∗∗*	*0.44∗∗∗∗*	0.25∗	*0.45∗∗∗∗*	0.22	0.14
PEC and eosinophil inflammation	**0.70∗∗∗∗**	**0.83∗∗∗∗**	**0.75∗∗∗∗**	**0.83∗∗∗∗**	**0.70∗∗∗∗**	**0.74∗∗∗∗**
PEC and eosinophil abscesses	**0.71∗∗∗∗**	**0.61∗∗∗∗**	**0.62∗∗∗∗**	*0.46∗∗∗∗*	*0.46∗∗*	0.16
PEC and eosinophil surface layering	**0.63∗∗∗∗**	**0.65∗∗∗∗**	**0.62∗∗∗∗**	**0.50∗∗∗∗**	*0.35∗*	0.21
PEC and surface epithelial alteration	0.11	0.29∗	*0.32∗∗*	*0.42∗∗∗∗*	*0.30*	*0.32∗∗*
PEC and basal zone hyperplasia	*0.42∗∗∗*	*0.49∗∗∗∗*	*0.34∗∗*	**0.58∗∗∗∗**	**0.70∗∗∗∗**	0.08
PEC and dilated intercellular spaces	0.09	0.06	−0.07	0.21	−0.13	0.03
PEC and dyskeratotic epithelial cells	0.00	0.18	−0.08	*0.41∗∗∗*	0.02	0.19
PEC and lamina propria fibrosis	*0.33∗*	*0.37∗*	*0.36∗∗*	0.24	0.28	−0.21
EoEHSS stage						
PEC and total score	**0.54∗∗∗∗**	**0.61∗∗∗∗**	*0.49∗∗∗∗*	*0.38∗∗∗*	*0.49∗∗*	*0.34∗∗*
PEC and inflammatory subscore	**0.58∗∗∗∗**	**0.74∗∗∗∗**	**0.63∗∗∗∗**	**0.63∗∗∗∗**	**0.68∗∗∗∗**	*0.42∗∗∗*
PEC and architectural subscore	0.26∗	*0.43∗∗∗∗*	0.23∗	*0.43∗∗∗∗*	*0.40∗*	0.29∗
PEC and eosinophil inflammation	**0.65∗∗∗∗**	**0.77∗∗∗∗**	**0.62∗∗∗∗**	**0.64∗∗∗∗**	**0.72∗∗∗∗**	N/A
PEC and eosinophil abscesses	**0.70∗∗∗∗**	**0.60∗∗∗∗**	**0.62∗∗∗∗**	*0.46∗∗∗∗*	*0.46∗∗*	0.16
PEC and eosinophil surface layering	**0.60∗∗∗∗**	**0.63∗∗∗∗**	**0.56∗∗∗∗**	**0.50∗∗∗∗**	*0.35∗*	0.21
PEC and surface epithelial alteration	0.04	0.26∗	0.22∗	*0.41∗∗∗*	*0.30*	*0.32∗∗*
PEC and basal zone hyperplasia	*0.30∗∗*	**0.52∗∗∗∗**	0.24∗	**0.58∗∗∗∗**	**0.70∗∗∗∗**	0.08
PEC and dilated intercellular spaces	−0.05	0.01	0.10	0.21	0.17	0.28∗
PEC and dyskeratotic epithelial cells	−0.01	0.19	−0.09	*0.41∗∗∗*	0.02	0.19
PEC and lamina propria fibrosis	*0.35∗*	*0.36∗*	*0.31∗*	0.21	0.28	−0.21

Coefficients formatted according to Cohen’s rule of thumb: ≥0.5 = strong (bold); ≥0.3–<0.5 = moderate (italic); <0.3 = weak (roman).

∗*P* < .05; ∗∗*P* < .01; ∗∗∗*P* < .001; ∗∗∗∗*P* < .0001.

N/A, not available; PEC, peak eosinophil count; qw, weekly.

### Correlations Between EoEHSS Grade and Stage Inflammatory and Architectural Subscores and Peak Eosinophil Count

As reported in Table 1, the correlations between EoEHSS grade and stage inflammatory subscores were generally strong at baseline, at week 24, and at week 52. The correlations between EoEHSS grade and stage architectural subscores were weak to moderate at baseline and overall moderate at week 24. At week 52, the correlations ranged from strong to weak.

### Correlations Between EoEHSS Grade and Stage Component Score and Peak Eosinophil Count

The correlations between the components of EoEHSS grade and stage score and peak eosinophil count are shown in Table 1. In general, moderate or strong correlations were seen between peak eosinophil count and EA, BZH, EI, and ESL at baseline, week 24, and week 52. Correlations between peak eosinophil count and other components of EoEHSS grade and stage score were generally weak or moderate.

### Correlations Between EoEHSS Grade and EREFS Scores

Correlations between EoEHSS grade and EREFS scores are reported in Table 2. Overall, EoEHSS grade total score moderately correlated with EREFS total score and with the EREFS inflammation subscore, although the correlation was weak in Part B at week 24 (0.14 for EREFS total score and 0.16 for EREFS inflammation subscore). EoEHSS grade inflammatory subscore moderately correlated with EREFS inflammation subscore, while the correlations between EoEHSS grade architectural and EREFS inflammation and remodeling subscores were generally weak.

**Table 2:** Correlation Between EoEHSS Total, Inflammatory, and Architectural Grade/Stage Scores and EREFS Total, Inflammation, and Remodeling Scores at Week 24 and Week 52

	Part A				Part A–C	
	Placebo (n = 39)		Dupilumab qw (n = 42)		Placebo/dupilumab qw (n = 37)	Dupilumab qw/dupilumab qw (n = 40)
	Baseline	Week 24	Baseline	Week 24	Week 52	Week 52
EoEHSS grade total and EREFS total	*0.35∗*	*0.41∗*	*0.40∗∗*	*0.41∗*	*0.44∗*	0.16
EoEHSS grade total and EREFS inflammation	*0.37∗*	*0.45∗*	**0.52∗∗∗**	*0.43∗*	*0.45∗*	0.28
EoEHSS grade total and EREFS remodeling	0.06	0.03	−0.07	0.14	0.06	−0.10
EoEHSS grade architectural and EREFS inflammation	0.15	0.26	*0.35∗*	**0.51∗∗**	*0.38∗*	*0.33*
EoEHSS grade architectural and EREFS remodeling	0.24	−0.06	−0.04	*0.44∗∗*	0.09	0.29
EoEHSS grade inflammatory and EREFS inflammation	*0.40∗*	**0.51∗∗**	**0.52∗∗∗**	*0.30*	*0.42∗*	0.19
EoEHSS grade inflammatory and EREFS remodeling	0.02	0.13	−0.03	−0.08	−0.01	*−0.38∗*
EoEHSS stage total and EREFS total	*0.39∗*	*0.43∗*	*0.39∗∗*	*0.43∗∗*	**0.51∗∗**	0.21
EoEHSS stage total and EREFS inflammation	*0.46∗∗*	*0.46∗*	**0.50∗∗∗**	*0.42∗*	*0.43∗*	*0.31*
EoEHSS stage total and EREFS remodeling	0.02	0.04	−0.05	0.21	0.27	−0.06
EoEHSS stage architectural and EREFS inflammation	0.06	*0.32*	*0.36∗*	*0.43∗∗*	*0.39∗*	0.29
EoEHSS stage architectural and EREFS remodeling	0.13	0.00	0.04	0.21	0.20	0.15
EoEHSS stage inflammatory and EREFS inflammation	**0.50∗∗∗**	**0.50∗∗**	*0.46∗∗*	0.28	*0.37∗*	0.10
EoEHSS stage inflammatory and EREFS remodeling	0.04	0.07	−0.06	0.17	0.21	*−0.42∗*

	Part B				Part B–C	
	Placebo (n = 79)		Dupilumab qw (n = 80)		Placebo/dupilumab qw (n = 37)	Dupilumab qw/dupilumab qw (n = 74)
	Baseline	Week 24	Baseline	Week 24	Week 52	Week 52
EoEHSS grade total and EREFS total	*0.43∗∗∗∗*	**0.69∗∗∗∗**	*0.40∗∗∗*	0.14	*0.46∗∗*	0.24
EoEHSS grade total and EREFS inflammation	*0.37∗∗∗*	**0.63∗∗∗∗**	*0.40∗∗∗*	0.16	*0.45∗∗*	0.20
EoEHSS grade total and EREFS remodeling	0.27∗	*0.43∗∗∗*	0.16	0.03	0.14	0.03
EoEHSS grade architectural and EREFS inflammation	*0.31∗∗*	**0.62∗∗∗∗**	*0.36∗∗*	−0.01	*0.36∗*	0.07
EoEHSS grade architectural and EREFS remodeling	0.29∗	*0.43∗∗∗*	−0.02	−0.10	0.12	−0.11
EoEHSS grade inflammatory and EREFS inflammation	*0.35∗∗*	**0.57∗∗∗∗**	*0.37∗∗∗*	0.29∗	*0.38∗*	*0.31∗*
EoEHSS grade inflammatory and EREFS remodeling	0.20	*0.43∗∗∗*	0.19	0.16	0.09	0.17
EoEHSS stage total and EREFS total	*0.43∗∗∗∗*	**0.62∗∗∗∗**	*0.31∗∗*	0.12	*0.44∗∗*	0.08
EoEHSS stage total and EREFS inflammation	*0.38∗∗∗*	**0.55∗∗∗∗**	*0.35∗∗*	0.20	*0.42∗∗*	0.13
EoEHSS stage total and EREFS remodeling	0.25∗	*0.39∗∗∗*	0.09	−0.06	0.17	−0.17
EoEHSS stage architectural and EREFS inflammation	*0.30∗∗*	**0.57∗∗∗∗**	*0.36∗∗*	0.18	*0.37∗*	0.17
EoEHSS stage architectural and EREFS remodeling	0.25∗	*0.35∗∗*	−0.01	−0.11	0.14	−0.18
EoEHSS stage inflammatory and EREFS inflammation	*0.37∗∗∗*	**0.55∗∗∗∗**	*0.37∗∗∗*	0.18	*0.48∗∗*	0.18
EoEHSS stage inflammatory and EREFS remodeling	0.18	*0.47∗∗∗∗*	0.17	0.12	0.06	0.16

EoEHSS architectural subscore: including basal zone hyperplasia, dilated intercellular spaces, dyskeratotic epithelial cells, and lamina propria fibrosis. EoEHSS inflammatory subscore: eosinophil density, eosinophil abscesses, eosinophil surface layering, and surface epithelial alteration.

EREFS inflammation subscore: edema, exudates, and furrows. EREFS remodeling subscore: rings and strictures. Coefficients formatted according to Cohen’s rule of thumb: ≥0.5 = strong (bold); ≥0.3–<0.5 = moderate (italic); <0.3 = weak (roman).

∗*P* < .05; ∗∗*P* < .01; ∗∗∗*P* < .001; ∗∗∗∗*P* < .0001.

qw, weekly.

### Correlations Between EoEHSS Stage and EREFS Scores

Overall, the correlations between EoEHSS stage total score and inflammatory and architectural subscores and EREFS total score and inflammation and remodeling subscores followed the same pattern as those observed for EoEHSS grade scores (Table 2). The correlation between EoEHSS stage inflammatory and EREFS inflammation subscores ranged from strong to weak in Parts A and B.

### Correlations Between EoEHSS Grade and Stage Total Score and DSQ Score

The correlations between EoEHSS grade/stage scores and DSQ score are shown in Table 3. Overall, only weak correlations were observed, except for a strong correlation for both EoEHSS grade and stage scores for the placebo/dupilumab group at week 52 in Part A–C, and a moderate correlation for the EoEHSS grade score for the dupilumab group at baseline in Part A.

**Table 3:** Correlation Between EoEHSS Grade/Stage Total and Component Scores and DSQ Score at Week 24 and Week 52

	Part A				Part A–C	
	Placebo (n = 39)		Dupilumab qw (n = 42)		Placebo/dupilumab qw (n = 37)	Dupilumab qw/dupilumab qw (n = 40)
	Baseline	Week 24	Baseline	Week 24	Week 52	Week 52
EoEHSS grade score						
DSQ and total score	−0.04	−0.13	*0.32∗*	0.15	**0.65∗∗∗**	−0.20
DSQ and eosinophil inflammation	0.12	−0.08	*0.30*	0.08	**0.61∗∗**	−0.28
DSQ and eosinophil abscesses	0.01	0.05	0.21	0.10	*0.44∗*	−0.17
DSQ and eosinophil surface layering	0.01	−0.09	*0.30*	−0.02	*0.45∗*	*−0.30*
DSQ and surface epithelial alteration	−0.07	0.07	0.18	0.00	*0.38*	−0.12
DSQ and basal zone hyperplasia	−0.11	−0.15	0.28	0.13	*0.31*	−0.25
DSQ and dilated intercellular spaces	0.17	0.11	−0.09	0.20	0.05	0.02
DSQ and dyskeratotic epithelial cells	0.06	0.16	−0.06	−0.06	0.27	−0.09
DSQ and lamina propria fibrosis	−0.08	−0.27	0.09	0.09	NE	−0.28
EoEHSS stage score						
DSQ and total score	−0.03	−0.10	0.26	0.12	**0.62∗∗**	−0.18
DSQ and eosinophil inflammation	0.08	−0.15	0.27	0.07	**0.67∗∗∗**	−0.26
DSQ and eosinophil abscesses	−0.01	0.11	0.20	0.10	*0.44∗*	−0.17
DSQ and eosinophil surface layering	0.00	−0.09	0.26	−0.02	*0.44∗*	*−0.30*
DSQ and surface epithelial alteration	−0.05	0.19	0.18	0.01	*0.38*	−0.13
DSQ and basal zone hyperplasia	−0.03	0.02	*0.32∗*	0.17	0.29	−0.26
DSQ and dilated intercellular spaces	*0.32∗*	0.02	−0.22	0.04	0.26	0.12
DSQ and dyskeratotic epithelial cells	0.07	0.16	−0.05	−0.06	0.27	−0.09
DSQ and lamina propria fibrosis	0.04	−0.20	0.10	0.03	NE	−0.28

	Part B				Part B–C	
	Placebo (n = 79)		Dupilumab qw (n = 80)		Placebo/dupilumab qw (n = 37)	Dupilumab qw/dupilumab qw (n = 74)
	Baseline	Week 24	Baseline	Week 24	Week 52	Week 52
EoEHSS grade score						
DSQ and total score	−0.02	0.16	0.11	0.11	0.05	−0.27∗
DSQ and eosinophil inflammation	−0.06	0.14	0.11	0.07	0.00	*−0.36∗∗*
DSQ and eosinophil abscesses	−0.02	0.00	0.04	0.05	−0.21	−0.13
DSQ and eosinophil surface layering	0.00	0.17	0.07	0.13	0.27	NE
DSQ and surface epithelial alteration	0.03	0.02	−0.05	0.06	*0.32*	−0.26
DSQ and basal zone hyperplasia	−0.04	0.19	0.23∗	0.09	0.08	0.01
DSQ and dilated intercellular spaces	−0.17	0.03	−0.09	0.09	−0.17	0.05
DSQ and dyskeratotic epithelial cells	−0.06	−0.11	0.02	0.19	0.14	−0.24
DSQ and lamina propria fibrosis	−0.03	0.01	0.16	0.01	−0.28	*0.37*
EoEHSS stage score						
DSQ and total score	−0.06	0.15	0.08	0.07	−0.23	−0.19
DSQ and eosinophil inflammation	−0.01	0.09	−0.02	0.13	−0.16	NE
DSQ and eosinophil abscesses	−0.03	−0.01	0.02	0.05	−0.21	−0.13
DSQ and eosinophil surface layering	0.01	0.17	0.03	0.12	0.27	NE
DSQ and surface epithelial alteration	0.02	−0.04	−0.06	0.05	*0.32*	−0.26
DSQ and basal zone hyperplasia	0.04	0.20	0.19	0.07	0.05	0.02
DSQ and dilated intercellular spaces	−0.28∗	−0.01	0.03	−0.02	*−0.30*	−0.13
DSQ and dyskeratotic epithelial cells	−0.05	−0.10	0.03	0.19	0.14	−0.24
DSQ and lamina propria fibrosis	0.03	0.15	0.22	−0.03	−0.28	*0.37*

Coefficients formatted according to Cohen’s rule of thumb: ≥0.5 = strong (bold); ≥0.3–<0.5 = moderate (italic); <0.3 = weak (roman).

∗*P* < .05; ∗∗*P* < .01; ∗∗∗*P* < .001.

NE, nonestimable.

### Correlations Between EoEHSS Grade and Stage Component Score and DSQ Score

In general, there was a weak correlation between EoEHSS grade and stage component scores and DSQ score at baseline and week 24 in Parts A and B (Table 3). One exception was the strong correlation seen between EI and DSQ score in the placebo/dupilumab group at week 52 in Part A‒C.

## Discussion

This study comprises a comprehensive analysis of the effect of dupilumab on multiple histopathologic changes in the esophageal biopsies of patients with EoE using the EoEHSS. Overall, dupilumab improved all the commonly observed EoEHSS grade and stage components from baseline to week 24. This is with the exception of DEC, likely because these were rarely observed even at baseline. The improvements in EoEHSS grade and stage scores were maintained up to 52 weeks for patients continuously on dupilumab; patients switching from placebo to dupilumab at week 24 showed similar improvements to those who received dupilumab during the placebo-controlled portion of the trial. These findings provide evidence that blocking type 2 inflammation with dupilumab leads to beneficial changes in multiple aspects of esophageal pathology beyond peak eosinophil counts in patients with EoE.^11^

For diagnosis, EoE requires pathologically high eosinophil levels in esophageal biopsies. In our analysis, we included eosinophil count by esophageal region (proximal, mid, and distal), assessed over a range of improvement thresholds. The proportion of patients achieving each of the thresholds was higher with dupilumab than placebo across all regions, affirming robust improvements in eosinophil counts throughout the esophagus with dupilumab. EoEHSS grade and stage scores strongly correlated with peak eosinophil count, as observed in previous studies.^11^^,^^21^ Peak eosinophil count also correlated with the EoEHSS grade and stage inflammatory subscores and some of the individual components of the EoEHSS grade and stage scores, including EA, BZH, EI, and ESL. Correlations between peak eosinophil count and the EoEHSS architectural subscore varied, perhaps due to the less common occurrence of some individual components (eg, evaluable LPF). Altogether, this nonetheless confirms that EoEHSS grade and stage scores parallel the current diagnostic and histologic treatment response standard.

The EoEHSS was used in randomized placebo-controlled studies testing oral budesonide suspension^22^ or RPC4046, a monoclonal antibody against IL-13,^23^ to treat EoE. Our report demonstrates the impact of a biologic agent on the individual components and component subscores in a trial testing a monoclonal antibody to treat EoE. These data confirm the positive effects of dupilumab on pathologic characteristics of EoE biopsies. This is also the first report to use the EoEHSS remission score in a randomized controlled trial testing therapy for EoE. In this study, the remission score demonstrates marked improvement in EoE microscopic pathology following treatment with dupilumab 300 mg qw, including long-term treatment at week 52. Some clinical studies reported improvements in esophageal eosinophil counts without accompanying improvements in patient-reported dysphagia, suggesting that eosinophils are not the only driver of pathology.^10^^,^^24^, ^25^, ^26^, ^27^ As such, it is important to measure a range of other histopathologic features to gain a full picture of disease pathology and the efficacy of treatments. Here, we observed that dupilumab improved eosinophilic aspects of the disease (eosinophil density, EA, ESL, and surface epithelial alteration) and other noneosinophilic disease features (BZH, DIS). Of note, a recent report suggests that reductions in BZH may be a noneosinophilic indicator of a beneficial response to therapy in patients with EoE,^28^ and that persistence of BZH after treatment may be a cause of ongoing symptoms.^29^ The component not improved with dupilumab was DEC. The low scores for this component at baseline, which have also been observed in other studies of patients with EoE,^11^ rendered improvements in this measure difficult to detect.

A remission score using the EoEHSS was recently developed.^12^ Importantly, patients meeting the remission threshold were shown to have improvements in EoE symptoms (symptoms assessed were dysphagia, nausea and vomiting, gastroesophageal reflux disease, and pain).^12^ While the present analysis was conducted post hoc, it is notable that the number of patients in remission following dupilumab treatment was higher vs placebo at week 24 and increased over time, and that 100% of patients in Part B (the largest arm of the study) were in EoEHSS remission at week 52.

Dupilumab biweekly treatment led to overall similar results, except for the proportion of patients in remission according to the EoEHSS remission score, which was lower compared with weekly treatment. This highlights the efficacy of the approved dupilumab 300 mg weekly dosage.

Another important modality for assessing esophageal health in patients with EoE is endoscopy, which in this study was scored using the validated EREFS scale.^18^ The correlations between EoEHSS grade and stage total score and EREFS total score were moderate to strong; a similar trend was observed between EoEHSS grade and stage inflammatory subscore and EREFS inflammation subscore. This contrasts with the overall weak correlation observed between EoEHSS grade and stage architectural subscore and EREFS remodeling subscore. One possible reason for the low strength of these correlations is temporal discordance, where microscopic inflammation may not immediately translate to grossly visible anatomic findings. Another reason could be that the EoEHSS and EREFS are measuring complementary disease features. Also, endoscopy assesses remodeling features such as rings and strictures, which are structural alterations likely reflecting pathologic changes that occur in the subepithelium.^30^ Since esophageal biopsies primarily assess the epithelium, with lamina propria captured in <50% of biopsies,^11^ endoscopic assessment combined with histology provides a more complete assessment of disease activity. This is an understudied concept that could be explored in future studies. Correlations between the total and component EoEHSS grade and stage scores and DSQ score were also overall weak, suggesting that the pathology measured by EoEHSS does not directly influence dysphagia symptoms. This reinforces the notion that it is important to assess patients in EoE clinical trials using multiple and complementary endpoints to gain a full picture of treatment efficacy. In addition, it will be important to look for correlations between pathologic abnormalities and symptoms other than dysphagia in future studies.

This analysis provides a comprehensive assessment of histologic changes in the esophagus of patients with EoE following dupilumab treatment. While previous studies reported on the effect of dupilumab on histologic aspects of the disease (ie, eosinophil count and EoEHSS grade and total scores),^16^^,^^17^^,^^31^ they do not report on the 8 histologic features of EoE that compose the EoEHSS total scores. This analysis provides a unique opportunity to evaluate these histopathologic changes in the context of other uniformly captured disease assessments. This analysis, however, has the following limitations: the post hoc nature of this research; the patient population was comprised predominantly of Caucasian patients (although EoE affects mostly Caucasian patients)^32^^,^^33^; the patients were all PPI nonresponsive and many were steroid nonresponsive,^31^ so whether the same results would be seen in a population with less severe disease is not known; and there were a small number of patients with available measurements of LPF, although those measurements indicate a favorable effect of dupilumab that should be explored in future studies.

## Conclusion

Weekly dupilumab 300 mg improved the extent and severity of histologic components of disease in adolescents and adults with EoE at 24 weeks, with improvements sustained to 52 weeks. Also, dupilumab increased the proportion of patients in EoEHSS remission at week 24 vs placebo, with further increases at week 52. Using EoEHSS to systematically survey histopathologic features of the disease (other than eosinophil count) provides a comprehensive assessment of epithelial changes in the esophagus and may ultimately better guide patient care.
