# Supplementary material for: Dupilumab Improves Histopathologic Features in Patients With Eosinophilic Esophagitis: LIBERTY EoE TREET Study Results
Source: Gastro Hep Adv. 2025 Feb 24;4(6):100646. doi: 10.1016/j.gastha.2025.100646 (PMC12143628; doi:10.1016/j.gastha.2025.100646)
Supplement: Supplementary Materials [file mmc1.docx]

| **Supplementary Table 1.** Definitions of the EoEHSS Stage and Grade Components and Their Associated Scores | | | | | | | |
| --- | --- | --- | --- | --- | --- | --- | --- |
| Eosinophilic inflammation: intraepithelial eosinophils are not normally found in esophageal biopsies; therefore, any intraepithelial eosinophils were considered abnormal | | | | | | | |
| Grade score | | | | Stage score | | | |
| Based on the quantity of eosinophils in the most inflamed high-power field; the threshold value for the highest grade was set at 4 times the threshold value of inflammation for EoE diagnosis | | | | Based on the amount of the biopsy that exhibited the threshold value for EoE diagnosis | | | |
| 0 | 1 | 2 | 3 | 0 | 1 | 2 | 3 |
| PEC = 0 | PEC < 15/hpf | PEC 15–59/hpf | PEC > 60/hpf | PEC 0–14/hpf | PEC ≥ 15/hpf in < 33% of hpfs | PEC ≥ 15/hpf in 33–66% of hpfs | PEC ≥ 15/hpf in > 66% of hpfs |
| Basal zone hyperplasia: the basal zone of esophageal squamous epithelium is composed of closely packed small cells and normally occupies ≤ 15% of the total epithelial thickness; the upper limit of the basal zone was defined as the level at which basal epithelial cell nuclei were separated by a distance equal to or greater than the diameter of a basal cell nucleus | | | | | | | |
| Grade score | | | | Stage score | | | |
| Based on the amount of total epithelial thickness occupied by the basal zone | | | | Based on the amount of the biopsy that exhibited any basal zone hyperplasia | | | |
| 0 | 1 | 2 | 3 | 0 | 1 | 2 | 3 |
| Absence of BZH | BZ occupies > 15% but < 33% of total epithelial thickness | BZ occupies 33–66% of total epithelial thickness | BZ occupies > 66% of total epithelial thickness | Absence of BZH | BZH (any grade > 0) in < 33% of epithelium | BZH (any grade > 0) in 33–66% of epithelium | BZH (any grade > 0) in > 66% of epithelium |
| Eosinophil abscesses: intraepithelial eosinophil group or aggregate in which eosinophils form solid masses and the epithelial architecture is disrupted so that adjacent eosinophils are not separated by intervening epithelial tissue | | | | | | | |
| Grade score | | | | Stage score | | | |
| Based on the density of eosinophils forming an abscess | | | | Based on the amount of the biopsy that exhibited eosinophil abscesses | | | |
| 0 | 1 | 2 | 3 | 0 | 1 | 2 | 3 |
| Absence of EA | Group of 4–9 eosinophils | Group of 10–20 eosinophils | Group of > 20 eosinophils | Absence of groups or aggregates of eosinophils | EA (any grade > 0) in < 33% of epithelium | EA (any grade > 0) in 33–66% of epithelium | EA (any grade > 0) in > 66% of epithelium |
| Eosinophil surface layering: linear alignment of at least 3 eosinophils in the upper third of the epithelium parallel to the lumen | | | | | | | |
| Grade score | | | | Stage score | | | |
| Based on the number of eosinophils forming the layer | | | | Based on the amount of the biopsy that exhibited any eosinophil surface layering | | | |
| 0 | 1 | 2 | 3 | 0 | 1 | 2 | 3 |
| SL of < 3 eosinophils | SL of 3–4 eosinophils | SL of 5–10 eosinophils | SL of > 10 eosinophils | Absence of SL | SL (any grade > 0) in < 33% of epithelium | SL (any grade > 0) in 33–66% of epithelium | SL (any grade > 0) in > 66% of epithelium |
| Dilated intercellular spaces: circumferential paracellular spaces in esophageal squamous epithelium that exhibit intercellular bridges | | | | | | | |
| Grade score | | | | Stage score | | | |
| Based on the degree of magnification required to see the intercellular bridges | | | | Based on the amount of the biopsy that exhibited intercellular bridges | | | |
| 0 | 1 | 2 | 3 | 0 | 1 | 2 | 3 |
| Absence of DIS at any magnification | Intercellular bridges in DIS visible at 400× magnification only | Intercellular bridges in DIS visible at 200× magnification | Intercellular bridges in DIS visible at 100× magnification or lower | Absence of DIS at any magnification | DIS (any grade > 0) in < 33% of epithelium | DIS (any grade > 0) in 33–66% of epithelium | DIS (any grade > 0) in > 66% of epithelium |
| Surface epithelial alteration: altered tinctorial properties of surface epithelium that manifest as increased (darker red) staining of surface epithelial cells, with or without associated eosinophil infiltrate | | | | | | | |
| Grade score | | | | Stage score | | | |
| Based on the amount of eosinophil infiltration in altered surface epithelium | | | | Based on the amount of the biopsy that exhibited any surface epithelial alteration | | | |
| 0 | 1 | 2 | 3 | 0 | 1 | 2 | 3 |
| Absence of SEA | SEA without eosinophils | SEA with any eosinophils | Shed altered surface epithelium admixed with numerous eosinophils consistent with exudate | Absence of SEA | SEA (any grade > 0) in < 33% of epithelium | SEA (any grade > 0) in 33–66% of epithelium | SEA (any grade > 0) in > 66% of epithelium |
| Dyskeratotic epithelial cells: individual cells with deeply eosinophilic cytoplasm and round small hyperchromatic nuclei | | | | | | | |
| Grade score | | | | Stage score | | | |
| Based on the quantity of dyskeratotic cells | | | | Based on the amount of the biopsy that exhibited any dyskeratotic epithelial cells | | | |
| 0 | 1 | 2 | 3 | 0 | 1 | 2 | 3 |
| Absence of DEC | 1 DEC/hpf | 2–5 DEC/hpf | > 5 DEC/hpf | Absence of DEC | DEC (any grade > 0) in < 33% of epithelium | DEC (any grade > 0) in 33–66% of epithelium | DEC (any grade > 0) in > 66% of epithelium |
| Lamina propria fibrosis: thickened connective tissue fibers in the lamina propria; lamina propria fibers that were arranged singly and had a diameter smaller than a basal layer nucleus were considered normal; fibers that were cohesive without increased diameter were considered abnormal, as were fibers with a diameter equal to or greater than a basal layer cell nucleus | | | | | | | |
| Grade score | | | | Stage score | | | |
| Based on the degree of fiber thickening | | | | Based on the amount of lamina propria that showed any fibrosis | | | |
| 0 | 1 | 2 | 3 | 0 | 1 | 2 | 3 |
| Absence of LPF | Fibers are cohesive and interfibril spaces cannot be demarcated | Fiber diameter equals the diameter of a basal cell nucleus | Fiber diameter exceeds the diameter of a basal cell nucleus | Absence of LPF | LPF (any grade > 0) in < 33% of lamina propria | LPF (any grade > 0) in 33–66% of lamina propria | LPF (any grade > 0) in > 66% of lamina propria |
| Definitions are reported in Collins, et al. 2017.^1^  BZ, basal zone; BZH, basal zone hyperplasia; DEC, dyskeratotic epithelial cells; DIS, dilated intercellular spaces; EA, eosinophil abscesses; EoE, eosinophilic esophagitis; EoEHSS, Eosinophilic Esophagitis Histology Scoring System; hpf, high-power field; LPF, lamina propria fibrosis; PEC, peak eosinophil count; SEA, surface epithelial alteration; SL, surface layering. | | | | | | | |

| **Supplementary Table 2.** EoEHSS Grade and Stage Component Scores at Baseline of Parts A and B | | | | | | | |
| --- | --- | --- | --- | --- | --- | --- | --- |
|  | Part A | | Part B | | | | |
|  | Placebo (n=39) | Dupilumab qw (n=42) | Placebo (n=79) | Dupilumab qw (n=80) | Dupilumab q2w (n=81) | |  |
| HSS grade component score, median (IQR) [n] | | | | | |  |  |
| Eosinophil inflammation | 2.3 (1.0) [39] | 2.3 (0.7) [42] | 2.3 (0.7) [79] | 2.3 (0.7) [80] | 2.3 (0.7) [81] | |  |
| Eosinophil abscesses | 0.3 (0.7) [39] | 0.3 (0.7) [42] | 0.3 (0.7) [79] | 0.5 (0.8) [80] | 0.3 (1.0) [81] | |  |
| Eosinophil surface layering | 0.7 (1.7) [39] | 1.2 (1.0) [42] | 1.3 (1.3) [79] | 1.3 (1.3) [80] | 1.3 (1.3) [81] | |  |
| Surface epithelial alteration | 0.3 (1.7) [39] | 0.7 (1.3) [42] | 0.7 (1.3) [79] | 0.7 (1.3) [80] | 0.7 (1.0) [81] | |  |
| Basal zone hyperplasia | 2.7 (1.0) [39] | 2.3 (1.7) [42] | 2.3 (1.3) [79] | 2.2 (1.0) [80] | 2.3 (1.0) [81] | |  |
| Dilated intercellular spaces | 2.0 (0.0) [39] | 2.0 (0.0) [42] | 2.0 (0.0) [79] | 2.0 (0.0) [80] | 2.0 (0.0) [81] | |  |
| Dyskeratotic epithelial cells | 0.0 (0.3) [39] | 0.0 (0.3) [42] | 0.0 (0.0) [79] | 0.0 (0.3) [80] | 0.0 (0.3) [81] | |  |
| Lamina propria fibrosis | 1.3 (1.5) [23] | 0.5 (1.0) [23] | 1.0 (1.0) [38] | 1.0 (1.3) [53] | 0.7 (1.5) [57] | |  |
| HSS stage component score, median (IQR) [n] | | | | | |  |  |
| Eosinophil inflammation | 2.3 (1.3) [39] | 2.2 (1.3) [42] | 2.0 (1.0) [79] | 2.3 (1.3) [80] | 2.3 (1.0) [81] | |  |
| Eosinophil abscesses | 0.3 (0.7) [39] | 0.3 (0.7) [42] | 0.3 (0.7) [79] | 0.3 (0.7) [80] | 0.3 (0.7) [81] | |  |
| Eosinophil surface layering | 0.7 (0.7) [39] | 0.7 (0.3) [42] | 0.7 (0.7) [79] | 0.7 (0.7) [80] | 0.7 (0.7) [81] | |  |
| Surface epithelial alteration | 0.3 (0.7) [39] | 0.3 (0.7) [42] | 0.3 (0.7) [79] | 0.3 (0.7) [80] | 0.3 (0.7) [81] | |  |
| Basal zone hyperplasia | 3.0 (1.0) [39] | 3.0 (1.0) [42] | 2.7 (1.0) [79] | 3.0 (1.0) [80] | 2.7 (1.0) [81] | |  |
| Dilated intercellular spaces | 3.0 (0.3) [39] | 3.0 (0.3) [42] | 3.0 (0.3) [79] | 3.0 (0.3) [80] | 3.0 (0.3) [81] | |  |
| Dyskeratotic epithelial cells | 0.0 (0.3) [39] | 0.0 (0.3) [42] | 0.0 (0.0) [79] | 0.0 (0.3) [80] | 0.0 (0.3) [81] | |  |
| Lamina propria fibrosis | 2.7 (1.5) [23] | 1.5 (3.0) [23] | 2.5 (3.0) [38] | 1.5 (3.0) [53] | 1.5 (3.0) [57] | |  |
| n reports the number of patients in which the component scores of the 3 regions (distal, mid, and proximal) were available.  EoEHSS, Eosinophilic Esophagitis Histology Scoring System; HSS, Histology Scoring System; IQR, interquartile range; q2w, every 2 weeks; qw, weekly. | | | | | | |  |

| **Supplementary Table 3.** EoEHSS Grade and Stage Component Scores at Baseline of Parts A–C and B–C | | | | | | |  |
| --- | --- | --- | --- | --- | --- | --- | --- |
|  | Part A–C | | Part B–C | | | |  |
|  | Placebo/ dupilumab qw (n=37) | Dupilumab qw/ dupilumab qw (n=40) | Placebo/ dupilumab qw (n=37) | Placebo/ dupilumab q2w (n=37) | Dupilumab qw/ dupilumab qw (n=74) | Dupilumab q2w/ dupilumab q2w (n=79) | |
| HSS grade component score, median (IQR) [n] | | | | | | | |
| Eosinophil inflammation | 2.0 (1.0) [33] | 0.7 (0.7) [38] | 2.0 (0.7) [37] | 2.3 (1.0) [37] | 0.7 (0.7) [74] | 0.7 (0.7) [79] | |
| Eosinophil abscesses | 0.3 (0.7) [33] | 0.0 (0.0) [38] | 0.3 (0.7) [37] | 0.0 (0.3) [37] | 0.0 (0.0) [74] | 0.0 (0.0) [79] | |
| Eosinophil surface layering | 0.7 (1.0) [33] | 0.0 (0.0) [38] | 0.7 (1.3) [37] | 0.7 (1.7) [37] | 0.0 (0.0) [74] | 0.0 (0.0) [79] | |
| Surface epithelial alteration | 0.7 (1.7) [33] | 0.0 (0.0) [38] | 0.5 (1.3) [37] | 0.0 (0.8) [36] | 0.0 (0.0) [74] | 0.0 (0.3) [79] | |
| Basal zone hyperplasia | 2.0 (0.7) [33] | 0.0 (0.3) [38] | 2.3 (1.7) [37] | 2.0 (2.0) [36] | 0.0 (0.0) [74] | 0.0 (0.0) [79] | |
| Dilated intercellular spaces | 2.0 (0.0) [33] | 1.8 (0.3) [38] | 2.0 (0.0) [37] | 2.0 (0.2) [36] | 2.0 (0.3) [74] | 2.0 (0.3) [79] | |
| Dyskeratotic epithelial cells | 0.0 (0.3) [33] | 0.0 (0.0) [38] | 0.0 (0.0) [37] | 0.0 (0.3) [36] | 0.0 (0.0) [74] | 0.0 (0.0) [79] | |
| Lamina propria fibrosis | 1.0 (1.8) [16] | 0.0 (0.0) [16] | 1.0 (1.0) [21] | 1.0 (0.3) [13] | 0.0 (0.0) [33] | 0.0 (0.0) [36] | |
| HSS stage component score, median (IQR) [n] | | | | | | | |
| Eosinophil inflammation | 2.3 (1.3) [33] | 0.0 (0.0) [38] | 2.0 (1.3) [37] | 2.0 (1.7) [37] | 0.0 (0.0) [74] | 0.0 (0.0) [79] | |
| Eosinophil abscesses | 0.3 (0.3) [33] | 0.0 (0.0) [38] | 0.3 (0.7) [37] | 0.0 (0.3) [37] | 0.0 (0.0) [74] | 0.0 (0.0) [79] | |
| Eosinophil surface layering | 0.3 (0.3) [33] | 0.0 (0.0) [38] | 0.3 (0.3) [37] | 0.3 (0.7) [37] | 0.0 (0.0) [74] | 0.0 (0.0) [79] | |
| Surface epithelial alteration | 0.3 (1.0) [33] | 0.0 (0.0) [38] | 0.3 (0.7) [37] | 0.0 (0.7) [36] | 0.0 (0.0) [74] | 0.0 (0.3) [79] | |
| Basal zone hyperplasia | 2.7 (1.0) [33] | 0.0 (0.3) [38] | 3.0 (1.3) [37] | 2.8 (1.7) [36] | 0.0 (0.0) [74] | 0.0 (0.0) [79] | |
| Dilated intercellular spaces | 3.0 (0.0) [33] | 3.0 (0.7) [38] | 3.0 (0.7) [37] | 3.0 (0.3) [36] | 2.7 (0.7) [74] | 3.0 (0.7) [79] | |
| Dyskeratotic epithelial cells | 0.0 (0.3) [33] | 0.0 (0.0) [38] | 0.0 (0.0) [37] | 0.0 (0.3) [36] | 0.0 (0.0) [74] | 0.0 (0.0) [79] | |
| Lamina propria fibrosis | 2.2 (3.0) [16] | 0.0 (0.0) [16] | 1.5 (3.0) [21] | 3.0 (1.3) [13] | 0.0 (0.0) [33] | 0.0 (0.0) [36] | |
| n reports the number of patients in which the component scores of the 3 regions (distal, mid, and proximal) were available.  EoEHSS, Eosinophilic Esophagitis Histology Scoring System; HSS, Histology Scoring System; IQR, interquartile range; q2w, every 2 weeks; qw, weekly. | | | | | | | |


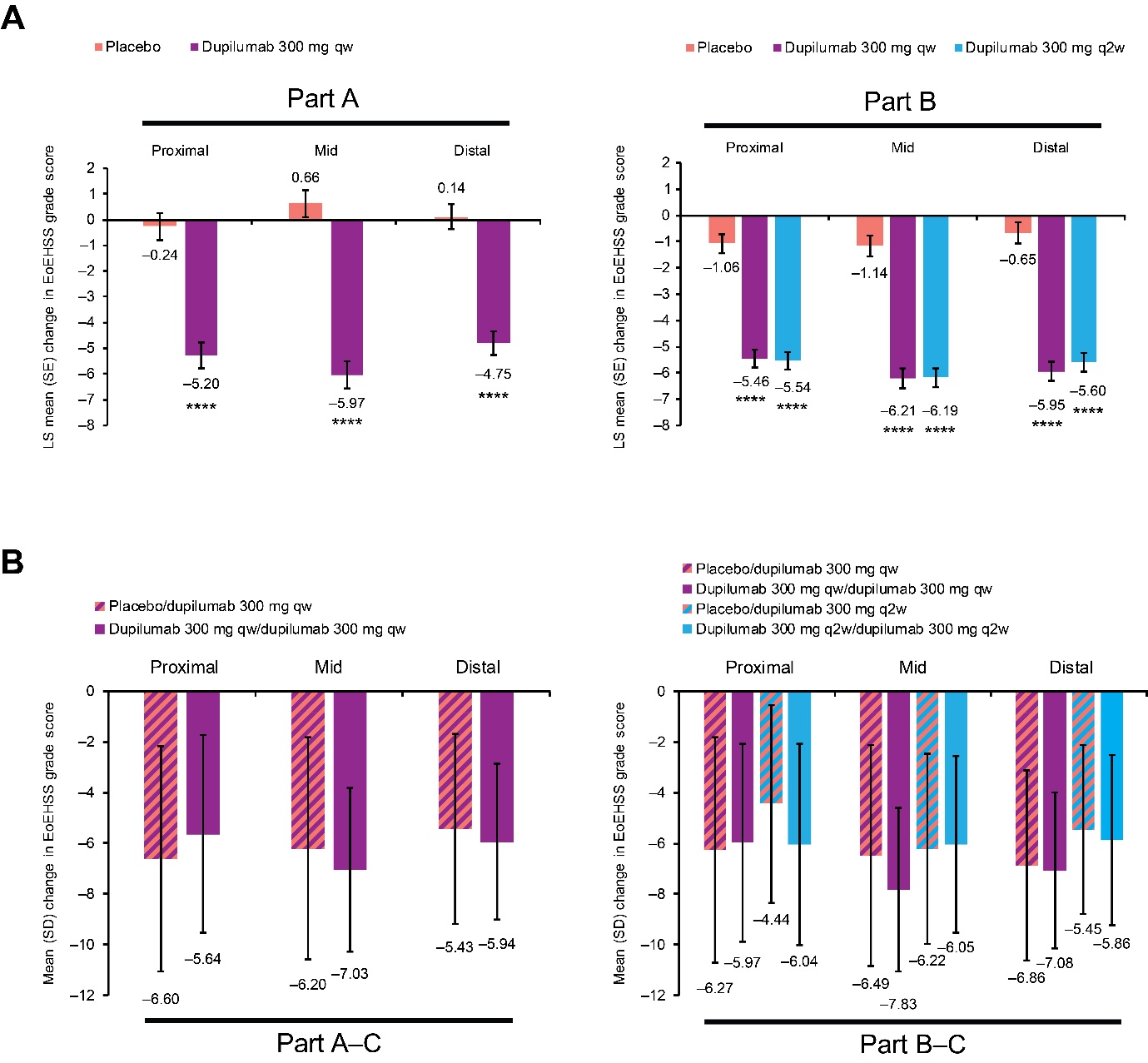


# Supplementary Figure 1. Change from baseline Part A or B in EoEHSS grade total score in proximal, mid, and distal regions at (A) week 24 and (B) week 52 (relative to baseline Part A or B).

*****P* < .0001.

EoEHSS, Eosinophilic Esophagitis Histology Scoring System; LS, least squares; q2w, every 2 weeks; qw, weekly; SD, standard deviation; SE, standard error.


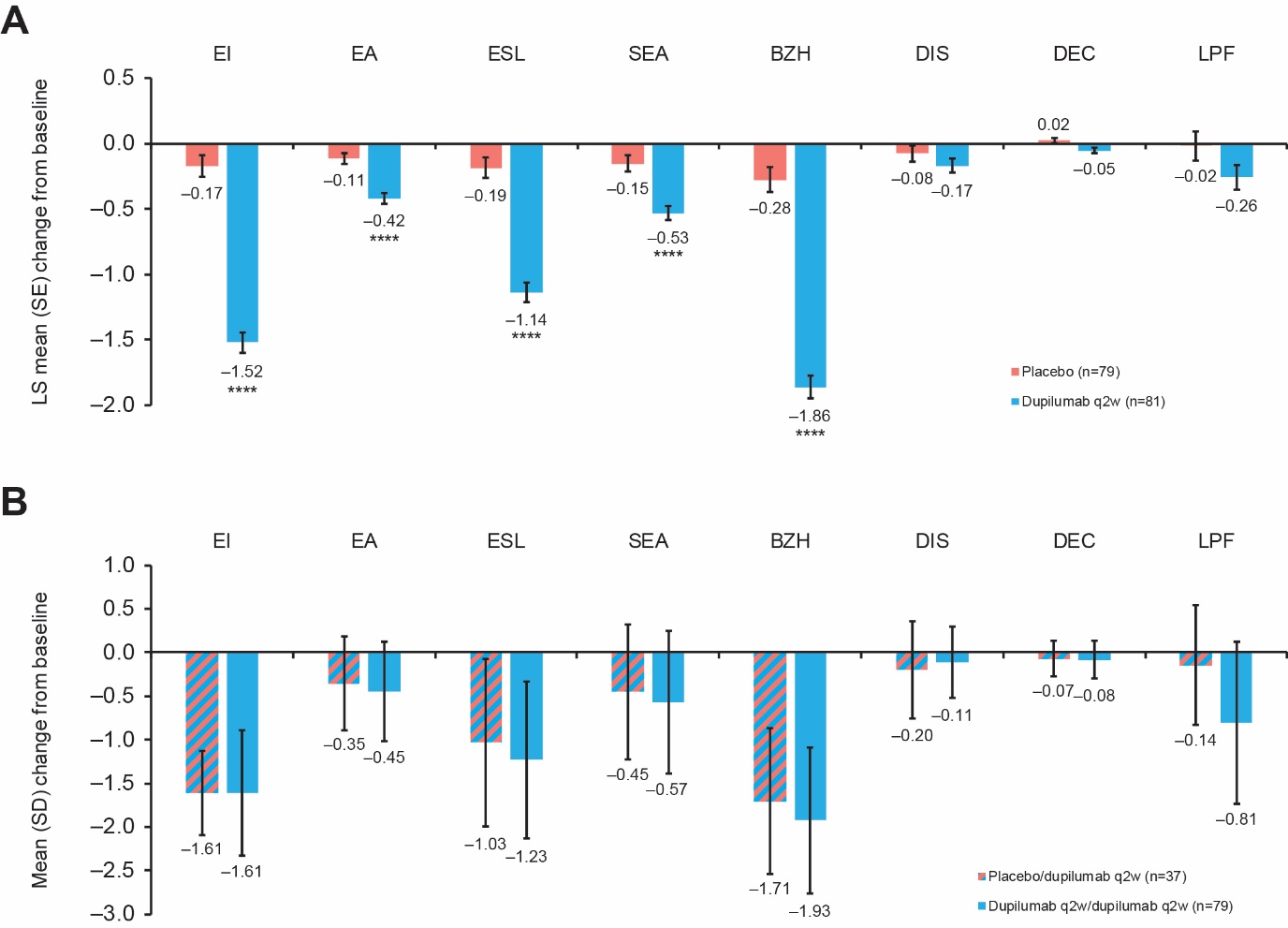


# Supplementary Figure 2. Change from baseline in EoEHSS grade component scores with dupilumab q2w at (A) week 24 and (B) week 52 (relative to baseline Part B).

*****P* < .0001.

The EoEHSS grade and stage mean component scores were derived as the mean of non-missing component scores of the 3 esophageal regions (proximal, mid, and distal); absolute change from baseline also required a measurement at baseline of Part B.

BZH, basal zone hyperplasia; DEC, dyskeratotic epithelial cells; DIS, dilated intercellular spaces; EA, eosinophil abscesses; EI, eosinophil inflammation; EoEHSS, Eosinophilic Esophagitis Histology Scoring System; ESL, eosinophil surface layering; LPF, lamina propria fibrosis; LS, least squares; q2w, every 2 weeks; SD, standard deviation; SE, standard error; SEA, surface epithelial alteration.


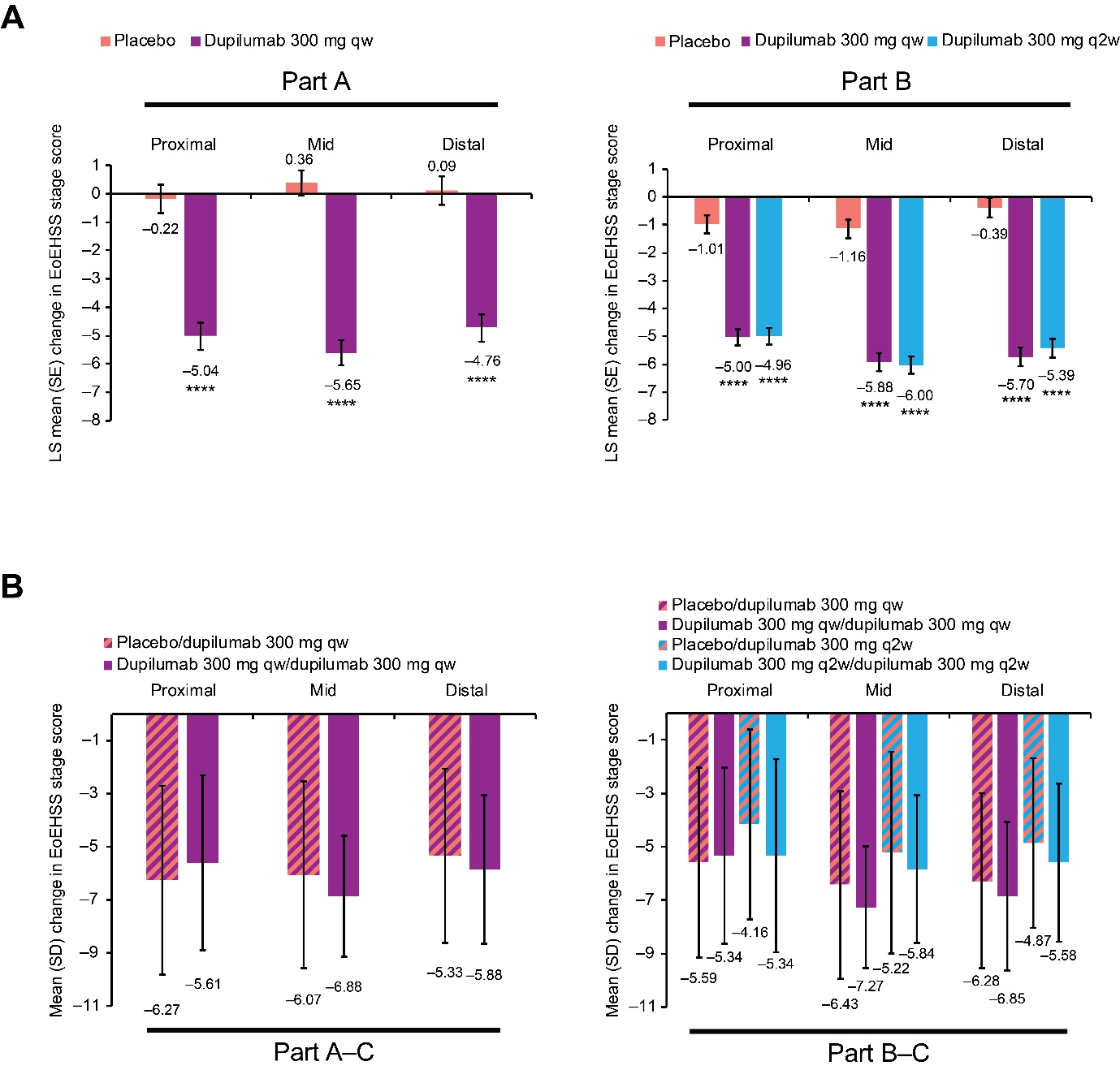


# Supplementary Figure 3. Change from baseline Part A or B in EoEHSS stage total score in proximal, mid, and distal regions at (A) week 24 and (B) week 52 (relative to baseline Part A or B).

*****P* < .0001.

EoEHSS, Eosinophilic Esophagitis Histology Scoring System; LS, least squares; q2w, every 2 weeks; qw, weekly; SD, standard deviation; SE, standard error.


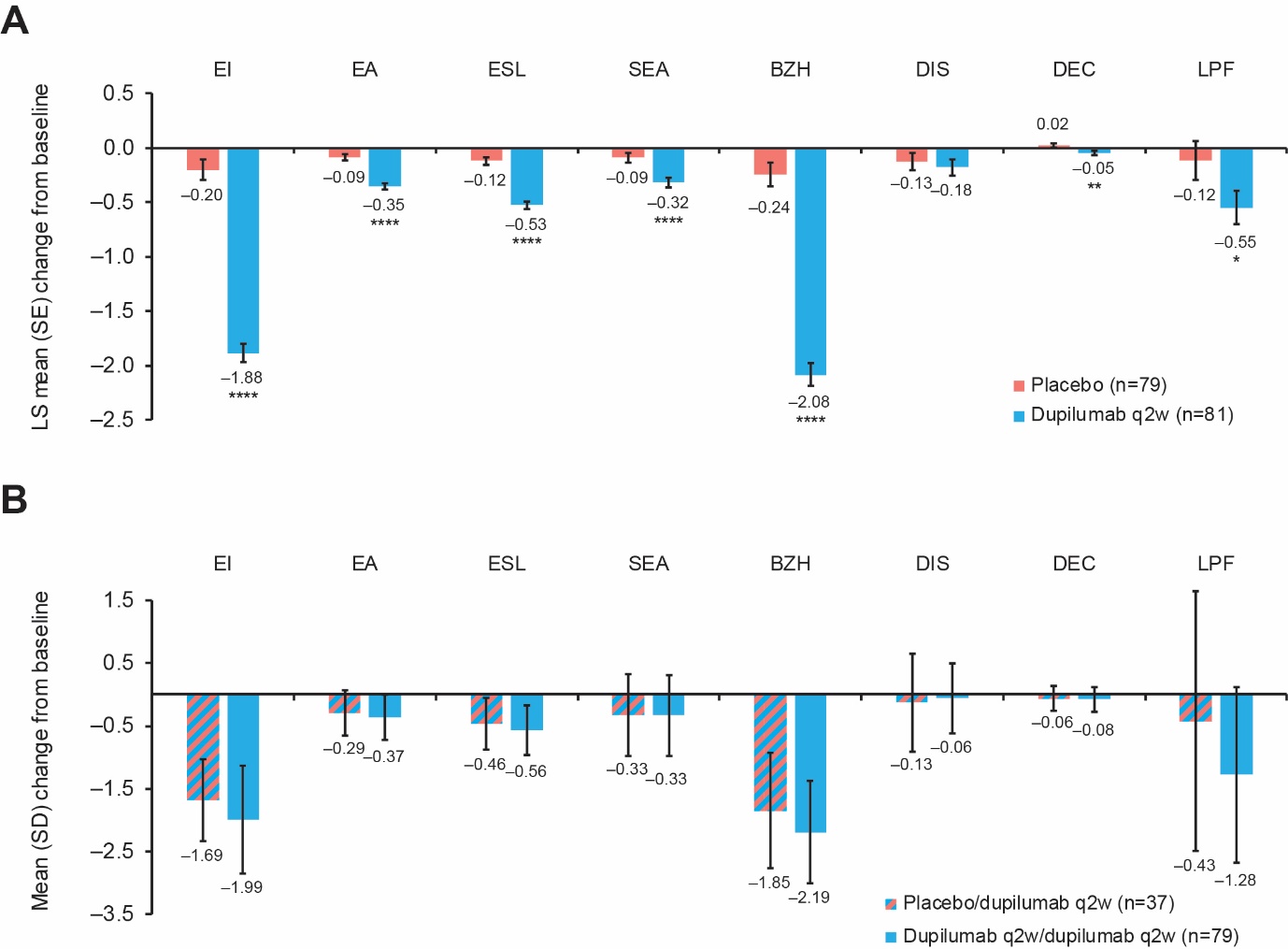


# Supplementary Figure 4. Change from baseline in EoEHSS stage component scores with dupilumab q2w at (A) week 24 and (B) week 52 (relative to baseline week 24).

**P* < .05; ***P* < .01; *****P* < .0001.

The EoEHSS grade and stage mean component scores were derived as the mean of non-missing component scores of the 3 esophageal regions (proximal, mid, and distal); absolute change from baseline also required a measurement at baseline of Part B.

BZH, basal zone hyperplasia; DEC, dyskeratotic epithelial cells; DIS, dilated intercellular spaces; EA, eosinophil abscesses; EI, eosinophil inflammation; EoEHSS, Eosinophilic Esophagitis Histology Scoring System; ESL, eosinophil surface layering; LPF, lamina propria fibrosis; LS, least squares; q2w, every 2 weeks; SD, standard deviation; SE, standard error; SEA, surface epithelial alteration.

**
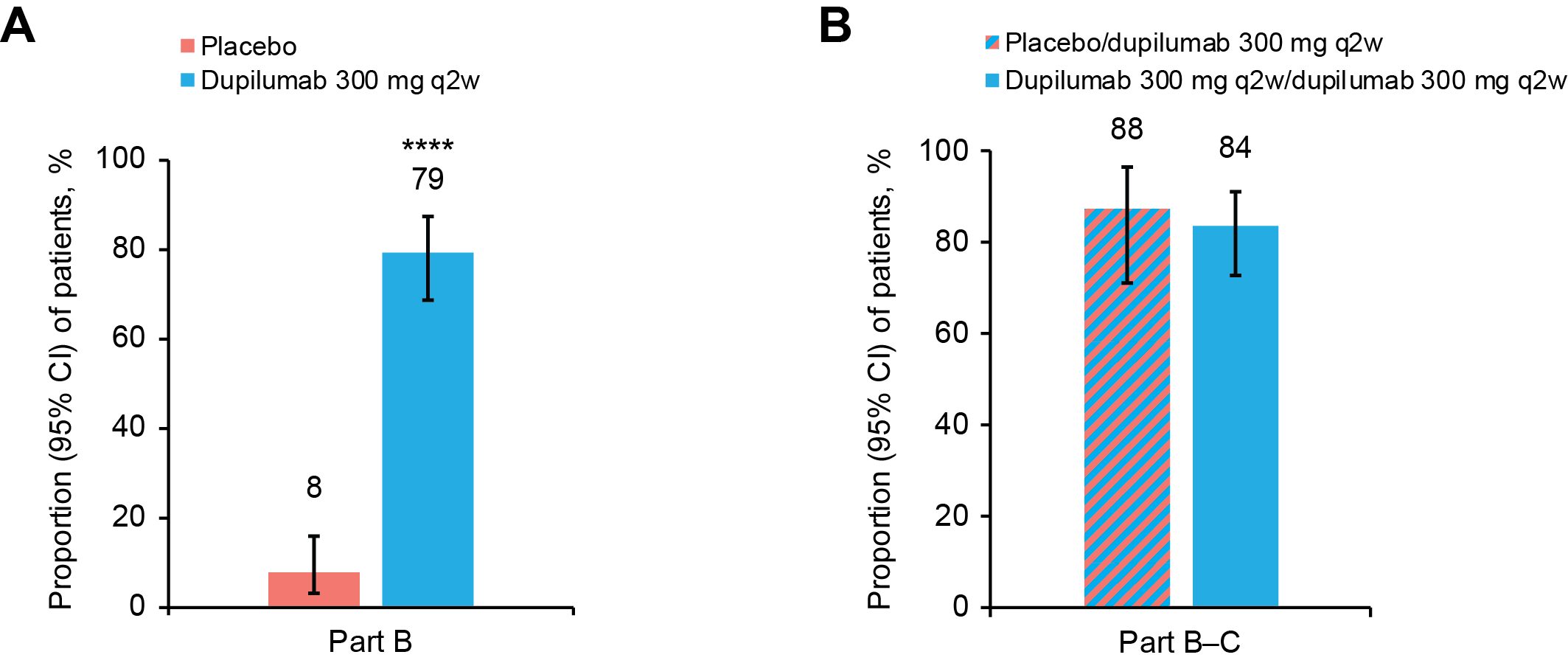
**

# Supplementary Figure 5. Proportion of patients in remission according to EoEHSS remission score with dupilumab q2w at (A) week 24 and (B) week 52.

*****P* < .0001.

CI, confidence interval; EoEHSS, Eosinophilic Esophagitis Histology Scoring System; q2w, every 2 weeks.


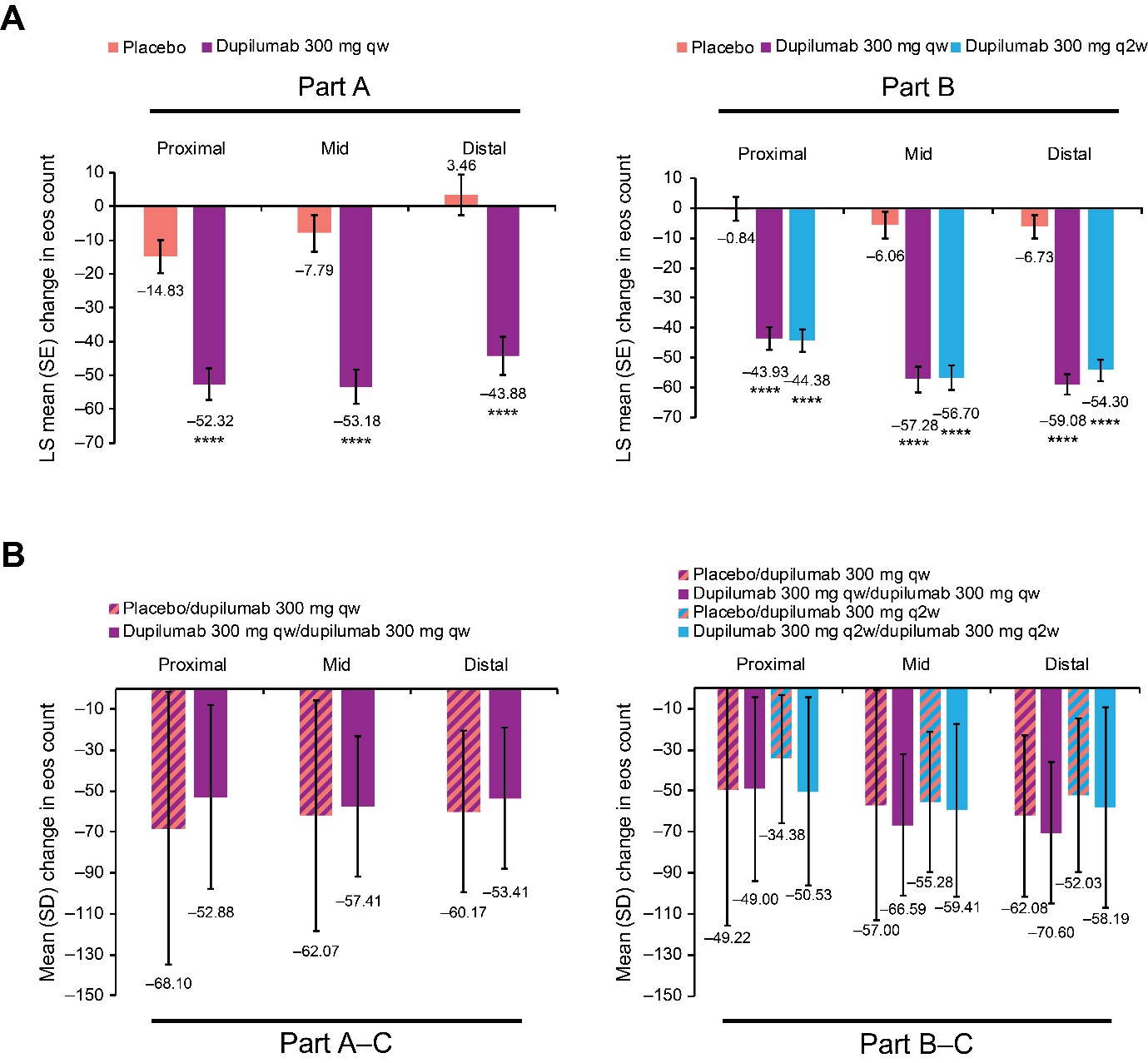


# Supplementary Figure 6. Change from baseline Part A or B in peak proximal, mid, and distal esophageal eosinophil count at (A) week 24 and (B) week 52 (relative to baseline Part A or B).

*****P* < .0001.

eos, eosinophils; LS, least squares; q2w, every 2 weeks; qw, weekly; SD, standard deviation; SE, standard error.


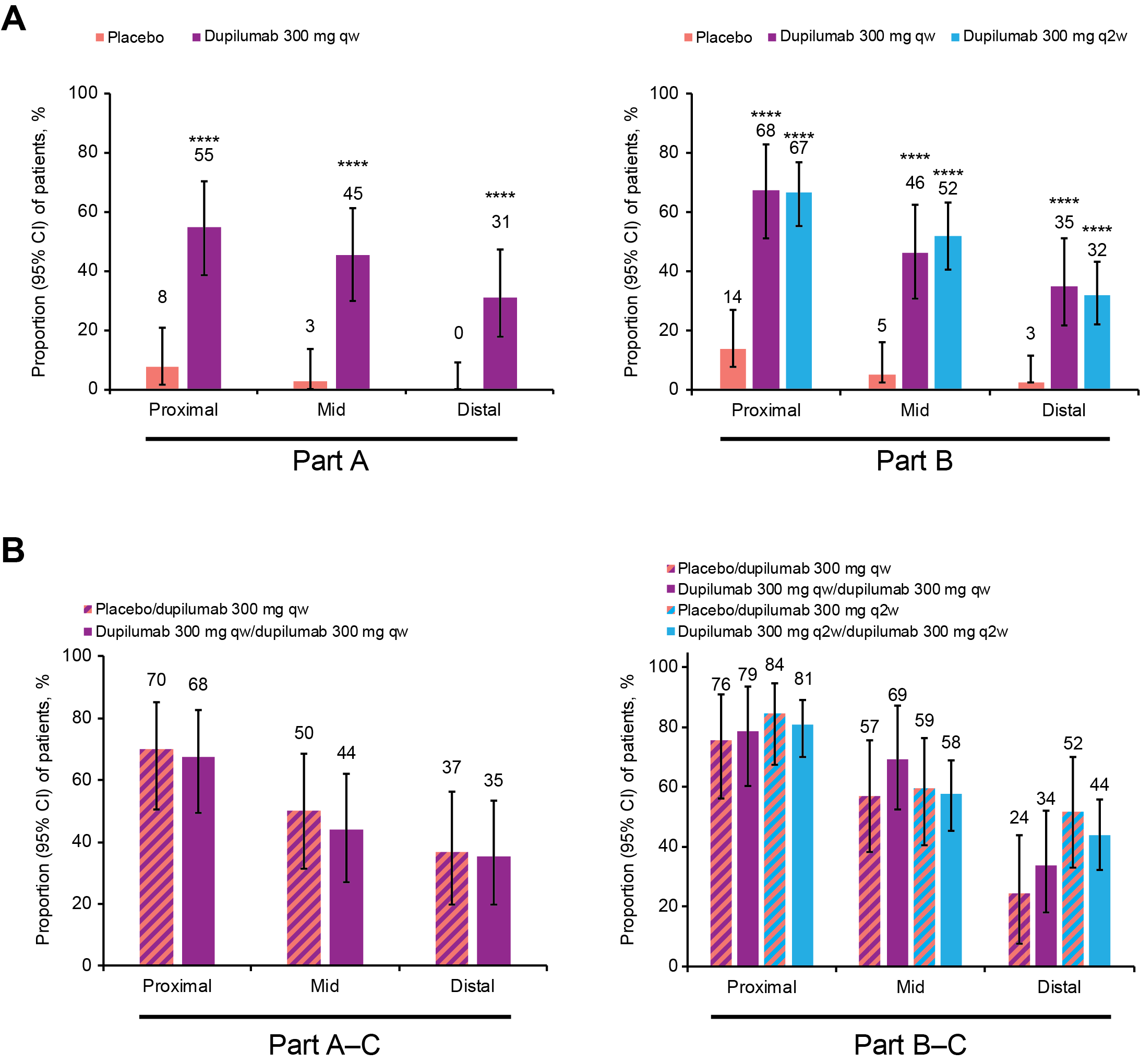


# Supplementary Figure 7. Proportion of patients with peak proximal, mid, and distal esophageal eosinophil count ≤ 1 eos/hpf at (A) week 24 and (B) week 52.

*****P* < .0001.

CI, confidence interval; eos/hpf, eosinophils per high-power field; q2w, every 2 weeks; qw, weekly.


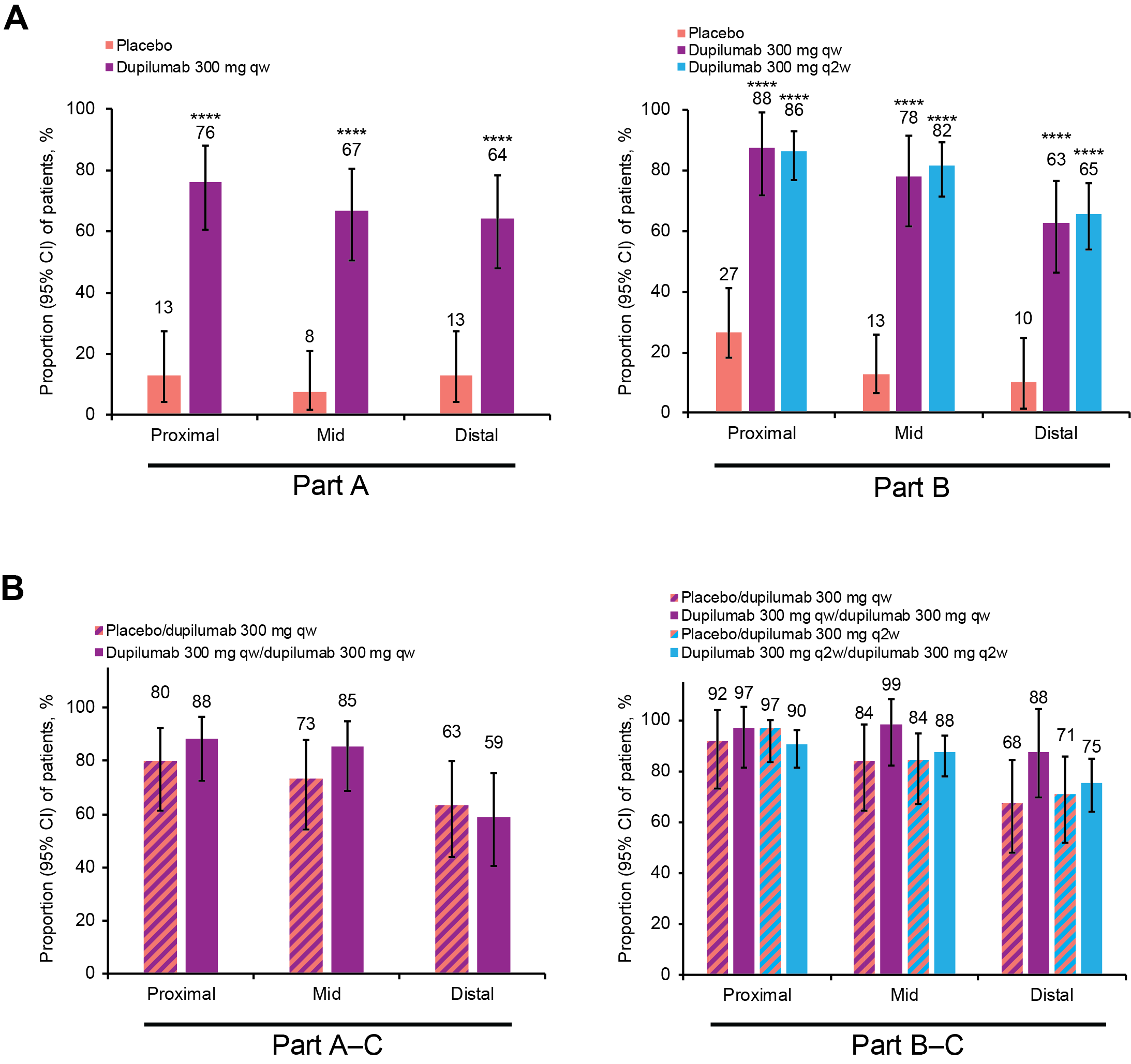


# Supplementary Figure 8. Proportion of patients with peak proximal, mid, and distal esophageal eosinophil count ≤ 6 eos/hpf at (A) week 24 and (B) week 52.

*****P* < .0001.

CI, confidence interval; eos/hpf, eosinophils per high-power field; q2w, every 2 weeks; qw, weekly.


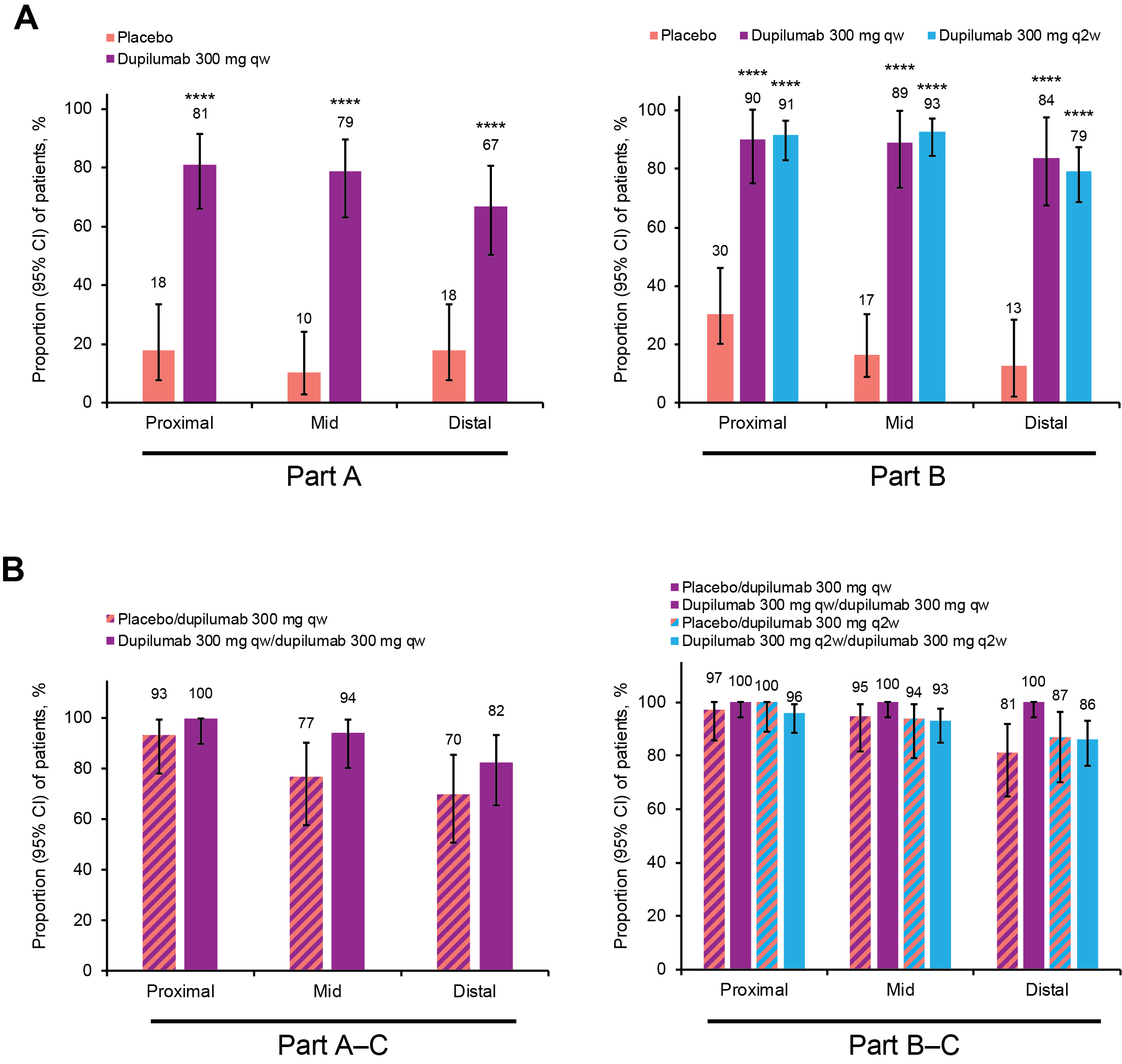


# Supplementary Figure 9. Proportion of patients with peak proximal, mid, and distal esophageal eosinophil count < 15 eos/hpf at (A) week 24 and (B) week 52.

*****P* < .0001.

CI, confidence interval; eos/hpf, eosinophils per high-power field; q2w, every 2 weeks; qw, weekly.

# Supplementary Material. Study protocol

The study protocol is available for download from this link:

<https://www.nejm.org/doi/pdf/10.1056/nejmoa2205982>

# References

1. Collins MH, Martin LJ, Alexander ES, et al. Newly developed and validated eosinophilic esophagitis histology scoring system and evidence that it outperforms peak eosinophil count for disease diagnosis and monitoring. Dis Esophagus 2017;30:1–8.
