# Supplementary material for: Dupilumab Improves Histopathologic Features in Patients With Eosinophilic Esophagitis: LIBERTY EoE TREET Study Results
Source: Gastro Hep Adv. 2025 Feb 24;4(6):100646. doi: 10.1016/j.gastha.2025.100646 (PMC12143628; doi:10.1016/j.gastha.2025.100646)
Supplement: Supplementary Materials [file mmc2.docx]

**Dupilumab Improves Histopathologic Features in Eosinophilic Esophagitis Patients: LIBERTY EoE TREET Study Results**

VIDEO SCRIPT presented by Margaret Collins

Target time ≤2.00 min; approximate timing: 2.00 min

| **Frame (time)** | **Natural language** | **Visual elements** | **Notes** |
| --- | --- | --- | --- |
| **1**  **(15s)** | Hello, I’m Dr Margaret Collins and on behalf of my coauthors I am excited to share the key findings from our analysis on the effect of dupilumab on the histologic features of eosinophilic esophagitis (EoE) from the LIBERTY EoE TREET phase 3 study | Talking head with title and affiliations on screen | eosinophilic esophagitis (EoE): mention abbreviations in brackets that it applies throughout the presentation  Affiliations: Division of Pathology and Laboratory Medicine, Department of Pediatrics, Cincinnati Children's Hospital Medical Center, University of Cincinnati College of Medicine, Cincinnati, OH, USA |
| **2**  **(35s)** | We used the EoE histology scoring system (EoEHSS) which assesses pathologic features beyond eosinophil infiltration  The EoEHSS includes 8 features, some directly related to eosinophil inflammation, and others not  The 8 components are scored separately for both grade (severity) and stage (extent) of pathology, each given a score for 0 to 3; the total observed score is then divided by the maximum possible score  The EoEHSS remission score incorporates the EoEHSS grade and stage scores, and the eosinophil count | Graphic plus text | Graphic: Icon of head and torso showing an esophagus that is inflamed  Text: The EoEHSS is a scoring system that can help recognize the presence of and evaluate both the severity and extent of EoE  Graphic (icon to represent each of the features to extract from the GA; icons order to match order of the mean change of the component scores presented in 4 and 5):  **Esophageal inflammation**   - Number of eosinophils present   **Basal zone hyperplasia**   - Thickness of the basal zone   **Eosinophil abscess**   - Presence of abscesses   **Eosinophil surface layering**   - Abnormal arrangement of eosinophils into lines   **Dilated intercellular spaces**   - Spaces between individual cells   **Surface epithelial alteration**   - Changes in how the cells pick up the dye used to stain resulting in the cells having pinker cytoplasm, with or without admixed eosinophils   **Dyskeratotic epithelial cells**   - Changes in how the inside of the cells pick up the dye, resulting in pinker cytoplasm in cells that are smaller than normal epithelial cells with smaller blue- to black-staining nuclei.   **Lamina propria fibrosis**   - Thickening of the connective tissue fibers in the deepest part of the biopsy, which can signify stricture formation and increased risk of food impaction |
| **3 (10s)** | The analysis included patients who received dupilumab 300 mg weekly or placebo for up to 52 weeks | Graphic – study design schematic | Take study design from GA |
| **4 (25s)** | In Part A, we found that dupilumab treatment for 24 weeks led to greater improvements in grade scores of several histopatholologic features compared with placebo  Improvements were maintained after 52 weeks in those continuing dupilumab and improved in those switching from placebo to dupilumab at Week 24  Similar findings were observed for Part B of the study | Graphic – schematic plots showing axes first then animate (wipe down) the bars of the plot | See Figure 1A & C in the accompanying paper and adapt to a schematic representation  Clear labelling of each:  **Week 24**  **Week 52**  Only Part A – C data shown due to time constraint; mention that Part B – C data show similar results. |
| **5 (5s)** | Observations were similar for the assessment of EoEHSS stage component scores | Graphic – schematic plots showing axes first then animate (wipe down) the bars of the plot | See Figure 2A & C in the accompanying paper and adapt to a schematic representation  Clear labelling of each:  **Week 24**  **Week 52** |
| **6 (10s)** | Patients were significantly more likely to achieve EoEHSS remission at Weeks 24 and 52 with dupilumab compared to placebo | Graphic – schematic plots showing axes first then animate (wipe up) the bars of the plot | See Figure 3A and B in the accompanying paper and adapt to a schematic representation  Clear labelling of each:  **Week 24**  **Week 52** |
| **7 (30s)** | We looked at how the changes in EoEHSS correlated with endoscopic and symptomatic measures of EoE. We used the EoE endoscopic reference score (EREFS), a validated diagnostic tool that survey inflammatory and remodelling signs of the esophagus, and the Dysphagia Symptom Questionnaire (DSQ), a patient-reported assessment that assesses the frequency and the severity of dysphagia.  We found that EoEHSS grade/stage scores correlated overall strongly with peak eosinophil count, moderately to strongly with EREFS, and overall weakly with DSQ score | Graphic – text boxes representing each of the scores assessed and an arrow linking them, the correlation strength represented by the thickness of the line; correlation to present for baseline, and Weeks 24 and 52 for each of the measures | Focus on correlations between EoEHSS grade/stage score and PEC/EREFS/DSQ total scores. |
| **8 (35s)** | In summary, weekly dupilumab 300 mg improved the extent and severity of histopathology in adolescents and adults with EoE at 24 weeks, sustained to 52 weeks. Also, dupilumab increased the proportion of patients in EoEHSS remission at Week 24 vs placebo, with further increases at Week 52  Overall, these data show us that using EoEHSS to systematically survey histopathologic features of EoE (beyond the eosinophil count) provides a comprehensive assessment of changes in the esophagus that may ultimately better guide patient care.  Thank you | Talking head with graphic and text | Graphic: Icon repeated from above of head and torso showing an esophagus that is inflamed  Text: Weekly dupilumab 300 mg improved the extent and severity of histologic components of disease in adolescents and adults with EoE at 24 weeks, with improvements sustained to 52 weeks  Displaying disclosures, acknowledgments, and study sponsorship |
