# Supplementary material for: Dupilumab Improves Histopathologic Features in Patients With Eosinophilic Esophagitis: LIBERTY EoE TREET Study Results
Source: Gastro Hep Adv. 2025 Feb 24;4(6):100646. doi: 10.1016/j.gastha.2025.100646 (PMC12143628; doi:10.1016/j.gastha.2025.100646)
Supplement: Supplementary Materials [file mmc3.pdf]

IND Number: 136142 Regeneron Pharmaceuticals, Inc.
EudraCT number: 2018-000844-25

Clinical Study Protocol

A PHASE 3, RANDOMIZED, 3-PART STUDY TO INVESTIGATE THE EFFICACY AND SAFETY OF DUPILUMAB IN ADULT AND ADOLESCENT PATIENTS WITH EOSINOPHILIC ESOPHAGITIS

**Compound**:	Dupilumab
**Clinical Phase:**	3
**Protocol Number:**	R668-EE-1774
**Protocol Version:**	R668-EE-1774 Amendment 5
**Amendment 5 Date of Issue:**	*See appended electronic signature page*
**Amendment 4 Date of Issue:**	16 April 2020
**Amendment 3 Date of Issue:**	10 March 2020
**Amendment 2 Date of Issue:**	18 April 2019
**Amendment 1 Date of Issue:**	04 October 2018
**Original Date of Issue:**	21 June 2018
**Medical /Study Director:**	[redacted]

[redacted]

AMENDMENT HISTORY

Amendment 5

The purpose of this protocol amendment is to adjust the sample size for Part B based on the results of Part A of the study, and to add an additional database lock after all patients in Part A complete study week 52 of Part C. Other changes were made for clarification and consistency. The following table outlines the changes made to the protocol and the rationale.

Description of Change	Rationale	Section Changed
Decreased the required sample size for Part B from 345 patients to 210 patients (70/arm) based on the sample size re-estimation for Part B, as previously outlined in Section 10.2 Justification of Sample Size.	This sample size re-estimation for Part B was based on results from Part A of the study in which a larger treatment effect on the Dysphagia Symptom Questionnaire (DSQ) was observed than in a phase 2 study with budesonide, previously used for sample size estimation.	Clinical Study Protocol Synopsis: Population, Statistical Plan<br>Section 5.1 Study Description and Duration<br>Figure 2 Study Design<br>Section 6.1 Number of Patients Planned<br>Section 7.5 Method of Treatment Assignment<br>Section 10.2 Justification of Sample Size
Added an additional database lock to occur after the last Part A patient has completed their week 52 visit of Part C, including patients who have terminated early in Part C.	To provide safety and efficacy data for analyses of durability of treatment effect	Section 10.4.9 Timing of Statistical Analysis
Added a subgroup analysis of the co-primary and secondary study variables in patients with previous swallowed topical corticosteroid use.	To assess the treatment effects of dupilumab in a subgroup who received topical steroid therapy.	Clinical Study Protocol Synopsis: Objective(s), Endpoint(s)<br>Section 2.2 Secondary Objectives<br>Section 4.2.2 Secondary Endpoints
Revised the adverse events of special interest (AESIs) definitions and adverse drug reactions (ADRs) for the dupilumab clinical development program.	For program consistency based on regulatory authority feedback and to align with the most recent Investigator's Brochure.	Section 3.2.3.2 Risks<br>Section 9.4.3 Other Events that Require Accelerated Reporting to Sponsor
Clarified that any additional analyses required to investigate the impact of COVID-19 on the safety evaluation will be specified in the SAP.	To address guidance from the US and EU regarding the conduct of clinical trials during the COVID-19 pandemic.	Section 10.4.4 Safety Analysis

VV-RIM-00127522-1.0 Approved - 05 Nov 2020 GMT-5:00

Description of Change	Rationale	Section Changed
Added the primary estimand for primary and key secondary endpoints and clarified the sensitivity analysis for co-primary endpoints including but not limited to the methods listed in protocol.	To implement a concept estimand in the primary analysis approaches for the primary and key secondary endpoints based on ICH E9 (R1).	Section 10.4.3.1 Primary Efficacy Analysis<br>Table 4 Summary of Primary Estimand for Co-primary Endpoints<br>Section 10.4.3.2.1 Analysis of Secondary Efficacy Endpoints in Part A and Part B<br>Table 5 Summary of Primary Estimand for Key Secondary Endpoints
Revised the Schedule of Events (SOE) to list TARC and Total IgE sample collection as separate rows, rather than listing in the same row with footnotes to clarify differences as before.	For clarification only; there are no changes to sample collection.	Table 1 Parts A and B Schedule of Events – Screening Period and Double-Blind Treatment Period<br>Section 8.1.1.1 Footnotes for Table 1, footnote #17 (removed)<br>Table 2 Table 2 Part C Schedule of Events – Extended Active Treatment Period<br>Section 8.1.1.2 Footnotes for Table 2, footnote #14 (removed)
Made minor clarifications and editorial corrections.	For clarification.	Throughout the document.

Amendment 4

The purpose of this protocol amendment is to protect patient safety and data integrity during the COVID-19 pandemic by allowing for certain study procedures to occur at delayed time points and/or outside of the clinic environment. All temporary mechanisms utilized, and deviations from planned study procedures are to be documented as being related to COVID-19 and will remain in effect only for the duration of the public health emergency. The following table outlines the changes made to the protocol and the rationale.

Description of Change	Rationale	Section Changed
Added provisions allowing study procedures to occur at delayed timepoints and/or outside of the clinic environment.<br>• Biopsy with endoscopies may be delayed for visit 11/week 24, visit 19/week 52, early termination, and before initiating rescue treatment<br>• At-home study drug dosing is permitted for all visits except for visit 3/day 1 and visit 11/week 24<br>• If the endoscopy with biopsies cannot occur due to COVID-19 restrictions, rescue treatment should be initiated without delay and these patients will be eligible to participate in Part C<br>• Safety and laboratory procedures (vital signs, weight, PK/ADA sample collection, hematology and chemistry urine sample collection) may be delayed or occur at-home when possible<br>• The patient-reported outcome questionnaires that are intended to be completed during clinic visits may be conducted via phone interviews after visit 3/day 1	To protect patient safety and data integrity in the context of COVID-19 restrictions.	Clinical Study Protocol Synopsis: Study Design<br>Section 3.2.3 Risk/Benefits of Participating in the Study<br>Section 3.2.3.2 Risks<br>Section 5.1 Study Description and Duration<br>Section 5.1.1.3 Placebo-Controlled Double-Blind Treatment Period (24 Weeks)<br>Section 6.2.3 Exclusion Criteria for Part C (the Extended Active Treatment Period), Criterion #5<br>Section 7.3 Rescue Treatments<br>Table 1 Parts A and B Schedule of Events – Screening Period and Double-Blind Treatment Period<br>Table 2 Part C Schedule of Events – Extended Active Treatment Period<br>Section 8.1 Schedule of Events<br>Section 8.1.1.1 Footnotes for Table 1, footnote #5, 9, 10, 11 (edited), footnote #6.a, 7.a, 13.a, 19 (added)<br>Section 8.1.1.2 Footnotes for Table 2, footnote #3, 6, 7, 8 (edited), footnote #2.a, 4.a, 15 (added)<br>Section 8.1.1.3 Footnotes for Table 3, footnote #3, 4, 5, 7.a (edited), footnote # 10 (added)<br>Section 8.2.2.3 EoE Impact Questionnaire (EoE-IQ)<br>Section 8.2.2.4 EoE Symptom Questionnaire<br>Section 8.2.2.5 Patient Global Impression of Change (PGIC)<br>Section 8.2.2.6 Patient Global Impression of Severity (PGIS)<br>Section 8.2.2.7 Total Nasal Symptom Score (TNSS)<br>Section 8.2.2.8 Standardized Rhinoconjunctivitis Quality of Life Questionnaire for ages 12+ [RQLQ(s)+12]<br>Section 8.2.2.9 Juniper Asthma Control Questionnaire (ACQ)<br>Section 8.2.2.10 Patient-Oriented Eczema Measure (POEM)<br>Section 10.4.3.1 Primary Efficacy Analyses

VV-RIM-00127522-1.0 Approved - 05 Nov 2020 GMT-5:00

**Description of Change**	**Rationale**	**Section Changed**
Added a provision allowing patients to extend their current assigned dose regimen of study drug (Parts A/B and/or C) until the post-baseline esophageal biopsy procedure(s) are performed.<br><br>This change is detailed in the Footnotes for the Schedule of Events. Additionally, the word "approximately" was added throughout the protocol for time points related to duration of treatment.	To protect patient safety and data integrity in the context of COVID-19 restrictions.	Section 3.2.1 Rationale for Study Design<br>Section 5.1 Study Description and Duration<br>Section 5.1.1.3 Placebo-Controlled Double-Blind Treatment Period (24 Weeks)<br>Section 5.1.2.3 Placebo-Controlled Double-Blind Treatment Period (24 Weeks)<br>Section 5.1.3 Part C (28-Week Extended Active Treatment Period<br>Table 1 Parts A and B Schedule of Events – Screening Period and Double-Blind Treatment Period<br>Table 2 Part C Schedule of Events – Extended Active Treatment Period<br>Section 8.1.1.1 Footnotes for Table 1, footnote #6a (added)<br>Section 8.1.1.2 Footnotes for Table 2, footnote #4a (added)<br>Section 10.4.3.1 Primary Efficacy Analyses

CONFIDENTIAL

VV-RIM-00127522-1.0 Approved - 05 Nov 2020 GMT-5:00

Amendment 3

The purpose of this amendment is to add transcriptome sequencing for analyzing RNA expression of eosinophilic esophagitis (EoE) and type 2 inflammation to the study secondary objectives and endpoints, and to add the European Quality of Life 5-dimension 3-level (EQ-5D-3L) Questionnaire to collect general health status of EoE patients. Additionally, the Eosinophilic Esophagitis-Endoscopic Reference Score (EoE-EREFS) procedure for Part B patients was revised to allow for centralized reading and scoring. Other minor changes were made to align with regulatory authority feedback and for general clarification. The following table outlines the changes made to the protocol and the rationale.

Study status as of Amendment 3: Part A enrollment complete and Part B enrollment ongoing.

**Change**	**Rationale**	**Section Changed**
Added endpoints for EoE and type 2 inflammation transcriptome signatures to the study secondary objectives and endpoints (from samples collected as a part of the original study procedures).	Transcriptome sequencing was added as an important indicator of disease activity.	Clinical Study Protocol Synopsis: Objective(s), Endpoint(s), Procedures and Assessments<br>Section 2.2 Secondary Objectives<br>Section 4.2.2 Secondary Endpoints<br>Section 8.2.6.1 EoE Diagnostic Panel and Type 2 Inflammation Transcriptomics (section added)<br>Section 10.4.3.2.1 Analysis of Secondary Efficacy Endpoints in Part A and Part B<br>Section 22 References
Added the EQ-5D-3L Questionnaire for newly screened patients at the screening or baseline visit only.	In order to collect general health status of EoE patients.	Table 1: Parts A and B Schedule of Events – Screening Period and Double-Blind Treatment Period<br>Section 8.1.1.1 Footnotes for Table 1, footnote #18 (footnote added)<br>Section 8.2.2.11 European Quality of Life 5-Dimensional Scale (EQ-5D) (section added)<br>Section 22 References
Revised the EoE-EREFS procedure for Part B enrolled patients to allow for centralized reading and scoring.	To minimize inter-rater variability for this endpoint.	Clinical Study Protocol Synopsis: Procedures and Assessments<br>Section 8.1.1.1 Footnotes for Table 1, footnote #12<br>Section 8.1.1.2 Footnotes for Table 2, footnote #9<br>Section 8.1.1.3 Footnotes for Table 3, footnote #6<br>Section 8.2.2.1 Endoscopy with EoE-EREFS and Biopsies, and Photographs<br>Section 10.4.3.2.1 Analysis of Secondary Efficacy Endpoints in Part A and Part B

CONFIDENTIAL

VV-RIM-00127522-1.0 Approved - 05 Nov 2020 GMT-5:00

Change	Rationale	Section Changed
Revised the adverse events of special interest (AESIs) definitions for the dupilumab clinical development program.	For program consistency based on regulatory authority feedback.	Section 9.4.3 Other Events that Require Accelerated Reporting to Sponsor
Revised the visit window for visit 11/week 24 and visit 19/week 52 to change ±7 days to +7 days.	For clarification to ensure the full 14 days of Dysphagia Symptom Questionnaire (DSQ) diary entries are collected just prior to these visits.	Table 1: Parts A and B Schedule of Events – Screening Period and Double-Blind Treatment Period<br>Table 2: Part C Schedule of Events – Extended Active Treatment Period
Revised an existing secondary endpoint to state: Proportion of patients achieving peak esophageal intraepithelial eosinophil count of <15 eos/hpf at week 24 (previously stated ≤15 eos/hpf).	To correct a typographical error and thus align with the inclusion criteria of ≥15 eos/hpf.	Clinical Study Protocol Synopsis: Endpoint(s)<br>Section 4.2.2 Secondary Endpoints
Clarified exclusion criterion #14 and prohibited medications to allow for one-time steroid use with the anesthetic preparation (not for EoE) used during the endoscopy procedures.	For clarification.	Section 6.2.2 Exclusion Criteria for Parts A and B, #14<br>Section 7.7.1 Prohibited Medications and Procedures
Clarified the requirement that all pre-dosing procedures (including endoscopy with biopsy) at the scheduled visit 11/week 24 must occur prior to dosing with Part C / Extended Active Treatment study drug.	For clarification.	Section 8.1.1.2 Footnotes for Table 2, footnotes #1 and #4
Clarified the assumption used for Part A sample size calculation.	For clarification.	Section 10.2 Justification of Sample Size
Minor language changes were made to clarify the definition of hospitalization.	For clarification.	Section 9.3.2 Serious Adverse Event
Section heading was updated to add to word circulating before biomarkers.	For clarification.	Section 8.2.6.2 Type 2 Inflammatory and Disease-Related Circulating Biomarkers

VV-RIM-00127522-1.0 Approved - 05 Nov 2020 GMT-5:00

Amendment 2

The following table outlines the changes made to the protocol and the affected sections:

Change	Section Changed
Per Health Authority request, changed Part C from open-label to a design with added placebo SC injections alternating with dupilumab 300 mg Q2W doses in order to mask the dosing regimen for Part B patients during this extended active treatment phase of the study.	Clinical Study Protocol Synopsis – Objectives, Study Design, Study Duration, Treatment(s), Endpoint(s), Statistical Plan<br>Section 3.2.1 Rationale for Study Design<br>Section 3.2.3.1 Benefits<br>Section 4.2.2 Secondary Endpoints<br>Section 5.1 Study Description and Duration<br>Figure 1 Study Flow Diagram<br>Figure 2 Study Design<br>Section 5.1.1.3 Placebo-Controlled, Double-Blind Treatment Period (24 Weeks)<br>Section 5.1.2.3 Placebo-Controlled, Double-Blind Treatment Period (24 Weeks)<br>Section 5.1.3 Part C (28-Week Extended Active Treatment Period)<br>Section 5.2 Planned Interim Analysis<br>Section 6.2.3 Exclusion Criteria for Part C (the Extended Active Treatment Period)<br>Section 7.1 Investigational and Reference Treatments<br>Section 7.3 Rescue Treatments<br>Section 7.4.2 Study Drug Discontinuation<br>Section 7.5 Method of Treatment Assignment<br>Section 7.5.1 Blinding<br>Section 7.6.1 Packaging, Labeling, and Storage<br>Section 8.1 Schedule of Events<br>Table 2 Part C Schedule of Events – Extended Active Treatment Period<br>Section 10.3.1 Efficacy Analysis Sets<br>Section 10.3.2 Safety Analysis Set<br>Section 10.4.3.2.2 Analysis of Secondary Efficacy Endpoints in Part C<br>Section 10.4.4.1 Adverse Events<br>Section 10.4.4.3 Treatment Exposure
Added a per protocol set to the defined efficacy analysis sets to assess the overall robustness of the analysis results. Added that any re-estimation of sample size for Part B will be documented in the Part B SAP before its database lock. If the re-estimated sample size requires an increase of the planned sample size in Part B by more than 86 total patients (25%), it will also be documented in a protocol amendment so as to inform Has, ECs, and investigators.	Clinical Study Protocol Synopsis – Statistical Plan<br>Section 6.1 Number of Patients Planned<br>Section 10.2 Justification of Sample Size<br>Section 10.3.1 Efficacy Analysis Sets<br>Section 10.3.2 Safety Analysis Set

VV-RIM-00127522-1.0 Approved - 05 Nov 2020 GMT-5:00

Change	Section Changed
Clarified safety analyses of the Part C extended active treatment period will only include patients who received at least 1 dose of Part C study drug.	
Added a substudy which may be performed at select sites and a secondary endpoint to the study for the endolumenal functional lumen imaging probe (EndoFLIP) procedure to measure esophageal distensibility during the esophagogastroscopy procedures at selected sites in approximately 150 adult patients.	Clinical Study Protocol Synopsis – Procedures and Assessments, Endpoint(s)<br>List of Abbreviations<br>Section 4.2.2 Secondary Endpoints<br>Table 1 Parts A and B Schedule of Events – Screening Period and Double-Blind Treatment Period<br>Table 2 Part C Schedule of Events – Extended Active Treatment Period<br>Table 3 Follow-up Period, Early Termination Visit, and Unscheduled Visit<br>Section 8.1.1.1 Footnotes for Table 1, footnote 2c<br>Section 8.1.1.2 Footnotes for Table 2, footnote 10<br>Section 8.1.1.3 Footnotes for Table 3, footnote 7<br>Section 8.2.2.1 Endoscopy with EoE-EREFS and Biopsies, and Photographs<br>Section 22 References
Added additional secondary endpoints for proportion of patients who receive rescue medications or procedures during the 24-week placebo-controlled treatment period and absolute change in EoE Stage Score from the EoEHSS from baseline to week 24.	Clinical Study Protocol Synopsis – Endpoint(s)<br>Section 4.2.2 Secondary Endpoints
Made revisions to sections related to patient screening and eligibility criteria to:<br>• clarify that baseline endoscopy with biopsy and EoE-EREFS do not need to be repeated for patients eligible for rescreening.<br>• clarify the requirement that all patients required to initiate high-dose PPI therapy prior to baseline may change to a different approved PPI medication during the study and to clarify the requirement for duration of treatment with high-dose PPI regimen prior to the baseline endoscopy to account for patients who present at the screening visit already using an acceptable PPI regimen.<br>• specify that exclusion of patients because of bleeding disorders or esophageal varices will be based on the opinion of the investigator if the condition would put the patient at undue risk for significant complications from an endoscopy procedure.	Clinical Study Protocol Synopsis – Treatment(s)<br>Section 5.1.1.1 Screening (Up to 12 Weeks)<br>Figure 3 Endoscopy/Biopsy Procedure Flow Diagram<br>Section 6.2.1 Inclusion Criteria for Parts A and B, #2<br>Section 6.2.2 Exclusion Criteria for Parts A and B, #8<br>Section 7.2 Background Treatment<br>Table 1 Parts A and B Schedule of Events – Screening Period and Double-Blind Treatment Period<br>Section 8.1.1.1 Footnotes for Table 1, footnote 4

Change	Section Changed
Made the following changes to the Schedule of Events:<br>• Added a column to the Schedule of Events specifying procedures for an Early Termination Visit that occurs during the 12-week safety follow-up period<br>• Changed collection of blood samples for FBR and DNA and RNA extraction from mandatory to optional.<br>• Changed the Visit Window for Visit 11 (from ±3 days to ±7 days) for consistency with all other study visits requiring an endoscopy procedure.<br>• Removed TARC analysis at Visit 8, Visit 12, and Visit 14 as well as removed Eotaxin-3 collection and analysis at Visit 5<br>• Added respiratory rate to the list of vital signs to be collected pre/post dose.<br>• Specified that the 21-day interval between visit 2 and visit 3 may be extended for patients whose DSQ compliance does not meet eligibility criteria (at least 11 daily entries during the 14 days immediately preceding the planned randomization/baseline date), as long as the total duration of the screening period does not exceed the 85-day maximum.<br>• Added clarification for the collection of TNSS, POEM, ACQ-5 and RQLQ(S)+12 PROs.<br>• Deleted reference to monthly visits from the Study Flow Diagram (Figure 1) for clarity.<br>• Clarified the visit window for Self-Administration of Study Drug at Home	Section 5.1.1.1 Screening (Up to 12 Weeks)<br>Figure 1 Study Flow Diagram<br>Section 5.1.1.3 Placebo-Controlled Double-Blind Treatment Period (24 Weeks)<br>Table 1 Parts A and B Schedule of Events – Screening Period and Double-Blind Treatment Period<br>Table 2 Part C Schedule of Events – Extended Active Treatment Period<br>Table 3 Follow-Up Period, Early Termination Visit, and Unscheduled Visit<br>Section 8.1.1.1 Footnotes for Table 1, footnotes 6, 11, 13, 17<br>Section 8.1.1.2 Footnotes for Table 2, footnotes 3, 4, 8, 11, 14<br>Section 8.1.1.3 Footnotes for Table 3, footnote 5<br>Section 8.2.3.1 Vital Signs<br>Section 8.2.7 Future Biomedical Research (FBR) Serum/Plasma (Optional)<br>Section 8.2.7.1 Genomics Substudy (Optional)<br>Section 10.4.4.2 Other Safety
Editorial changes were made throughout the protocol for further clarification, consistency, and completeness, and, if appropriate, correction.<br>All references to the "study reference manual" have been replaced with 'study regulatory binders.'	Clinical Study Protocol Synopsis – Endpoints, Procedures and Assessments<br>Section 4.2.2 Secondary Endpoints<br>Section 5.1 Study Description and Duration<br>Section 5.1.1.3 Placebo-Controlled Double-Blind Treatment Period (24 Weeks)<br>Section 6.2.1 Inclusion Criteria for Parts A and B<br>Section 7.5.1 Blinding<br>Section 8.1.1.1 Footnotes for Table 1, footnote 15<br>Section 8.2.2.1 Endoscopy with EoE-EREFS and Biopsies, and Photographs<br>Section 8.2.2.2 Dysphagia Symptom Questionnaire (DSQ) - Patient-reported Outcome (PRO)<br>Section 8.2.2.3 EoE Impact Questionnaire (EoE-IQ)<br>Section 8.2.3.4 Electrocardiogram<br>Section 8.2.3.5 Laboratory Testing

VV-RIM-00127522-1.0 Approved - 05 Nov 2020 GMT-5:00

**Change**	**Section Changed**
	Section 8.2.6 Pharmacodynamic and Exploratory Biomarker Procedures<br>Section 9.4.1 Adverse Events<br>Section 9.4.2 Serious Adverse Events<br>Section 9.4.4 Reporting Adverse Events Leading to Withdrawal from the Study<br>Section 10.4.4.1 Adverse Events<br>Section 10.4.4.3 Treatment Exposure<br>Section 10.4.6 Analysis of Anti-Drug Antibody Data

VV-RIM-00127522-1.0 Approved - 05 Nov 2020 GMT-5:00

Amendment 1

The following table outlines the changes made to the protocol and the affected sections:

Change	Section Changed
Added an exclusion criterion: known systemic hypersensitivity to dupilumab or the excipients of the drug product	Section 6.2.2 Exclusion Criteria for Parts A and B, #34 (added)<br>Section 6.2.3 Exclusion Criteria for Part C (the Open-Label Treatment Period), #6 (added)
Exclusion Criterion # 26: replaced serum creatinine threshold with estimated glomerular filtration rate (eGFR).	Section 6.2.2 Exclusion Criteria for Parts A and B, #26
Exclusion Criterion #27: replaced the example for severe renal conditions of "patients on dialysis" with "severe nephrotic syndrome".	Section 6.2.2 Exclusion Criteria for Parts A and B, #27
Exclusion Criterion #33: Added clarification that the reliability of sexual abstinence needs to be evaluated in relation to the duration of the clinical trial and the preferred and usual lifestyle of the patient based on Clinical Trial Facilitation Group guideline on contraception.	Section 6.2.2 Exclusion Criteria for Parts A and B, #33
Clarified criteria for resumption of treatment after study drug has been temporarily discontinued because of a severe laboratory abnormality.	Section 7.4.2.2 Reasons for Temporary Discontinuation of Study Drug

CONFIDENTIAL

VV-RIM-00127522-1.0 Approved - 05 Nov 2020 GMT-5:00

CLINICAL STUDY PROTOCOL SYNOPSIS

**Title**	A Phase 3, Randomized, 3-Part Study to Investigate the Efficacy and Safety of Dupilumab in Adult and Adolescent Patients with Eosinophilic Esophagitis (EoE)
**Site Location(s)**	Global
**Objective(s)**	**Primary Objectives**<br>This study consists of three parts: Part A and Part B are 24-week treatment, randomized, double-blind, placebo-controlled study phases and Part C is a 28-week, extended active treatment phase that will enroll patients from Part A and Part B.<br>The primary objectives by study part are:<br>• ***<u>Part A:</u>*** To determine the treatment effect of dupilumab compared with placebo in adult and adolescent patients with EoE after 24 weeks of treatment as assessed by histological and clinical measures and to inform/confirm the final sample size determination for Part B.<br>• *Note: Patients enrolled in Part A may not participate in Part B.*<br>• ***<u>Part B:</u>*** To demonstrate the efficacy of dupilumab treatment compared with placebo in adult and adolescent patients with EoE after 24 weeks of treatment as assessed by histological and clinical measures.<br>• ***<u>Part C:</u>*** To assess the safety and efficacy of dupilumab treatment in adult and adolescent patients with EoE after up to 52 weeks of treatment as assessed by histological and clinical measures.<br>**Secondary Objectives**<br>• To evaluate the safety, tolerability, and immunogenicity of dupilumab treatment for up to 52 weeks in adult and adolescent patients with EoE<br>• To explore the relationship between dupilumab concentration and responses in adult and adolescent patients with EoE, using descriptive analyses<br>• To evaluate the effects of dupilumab on transcriptomic signatures associated with EoE and type 2 inflammation<br>• To demonstrate the efficacy of dupilumab treatment compared to placebo after 24 weeks and 52 weeks of treatment in adult and adolescent patients with EoE who have previously received swallowed topical corticosteroids
**Study Design**	**Part A**<br>***<u>Screening (Up to 12 Weeks)</u>***<br>After obtaining informed consent/assent, patients will initially be assessed for study eligibility at visit 1.<br>Study participants are required to have a confirmed diagnosis of EoE which may be established either by a prior historical biopsy or by biopsies performed during the screening period.<br>All patients meeting clinical and laboratory eligibility criteria will undergo endoscopy with biopsies at visit 2 to establish a baseline reference measure. For patients without a historical biopsy, the visit 2 biopsies will serve as both confirmation of EoE diagnosis and the baseline reference measure. All biopsies

performed during this study will be evaluated by pathologists at a central pathology laboratory who will be blinded to treatment assignment.

After confirmation of EoE diagnosis, patients will be given an electronic diary (eDiary) to begin recording dysphagia symptoms on a daily basis.

Randomization

At the baseline visit (visit 3), patients who continue to meet eligibility criteria will enter the 24-week placebo-controlled, double-blind treatment period and will be randomized in a 1:1 ratio to dupilumab 300 mg once weekly (QW) or placebo administered subcutaneously (SC).

Placebo-Controlled Double-Blind Treatment Period (24 Weeks)

The co-primary endpoints will be assessed at week 24, one week after the last dose of study drug during the double-blind treatment period to inform/confirm the final sample size determination for Part B.

At the end of the double-blind treatment visit (week 24), eligible patients in Part A may enter a 28-week extended active treatment period (Part C). Patients not participating in Part C will enter a 12-week follow-up period.

Patients enrolled in Part A will not be eligible to participate in Part B.

Part B

Enrollment for Part B will begin immediately after the last patient is enrolled in Part A.

Screening (Up to 12 Weeks)

The screening procedures for Part B are identical to those described above for Part A.

Randomization

At the baseline visit (visit 3), patients who continue to meet eligibility criteria will enter the 24-week double-blind treatment period and will be randomized in a 1:1:1 ratio to dupilumab 300 mg QW, dupilumab 300 mg once every two weeks (Q2W), or placebo administered SC.

Placebo-controlled Double-Blind Treatment Period (24 Weeks)

Procedures for Part B are identical to those for Part A.

At the end of the double-blind treatment visit (week 24), eligible patients in Part B may enter a 28-week extended active treatment period (Part C). Patients not participating in Part C will enter a 12-week follow-up period.

Part C (28-Week Extended Active Treatment Period)

At the end of the double-blind treatment visit (week 24), eligible patients in Part A and Part B may enter a 28-week extended active treatment period where all patients will receive active treatment with dupilumab but only patients in Part B will be blinded to treatment regimen in Part C.

Patients from Part A who are randomized to placebo during the double-blind treatment period will receive dupilumab 300 mg QW during Part C. Patients from Part A who are randomized to dupilumab 300 mg QW during the double-blind treatment period will continue to receive dupilumab 300 mg QW during Part C.

Patients from Part B who are randomized to placebo during the double-blind treatment period will be re-randomized in a 1:1 ratio to dupilumab 300 mg QW or dupilumab 300 mg Q2W.

Patients randomized to dupilumab 300 mg Q2W will also receive matching placebo administered Q2W alternating with dupilumab doses so the injection frequency will match the other group for regimen-blinding purposes. All other

VV-RIM-00127522-1.0 Approved - 05 Nov 2020 GMT-5:00

	patients will remain on the same dupilumab dose regimen to which they are randomized during the double-blind treatment period.<br>**Follow-up Period (12 Weeks)**<br>All patients will be followed up for an additional 12 weeks after completing Part C or, if ineligible for Part C, immediately following Part A or B.<br>**Rescue Treatment**<br>If medically necessary, rescue medications (systemic and/or swallowed topical corticosteroids) or emergency esophageal dilation are allowed in study patients in Parts A, B and C. An endoscopy with biopsies will be performed prior to the initiation of rescue therapy unless COVID-19 restrictions prohibit this procedure. If the endoscopy with biopsies cannot occur due to COVID-19 restrictions, rescue treatment should not be delayed, and these patients will be eligible for Part C. Part C treatment will be initiated per the schedule of events and only at an in-clinic visit. Patients receiving rescue therapy may continue to receive study drug. They will remain blinded and will be asked to return to the clinic for all remaining study treatment visits and participate in all study assessments according to the schedule of events. However, they will not undergo any scheduled endoscopy/biopsies subsequent to the date of rescue.
**Study Duration**	The duration of the study for a patient (excluding the screening period) is approximately 64 weeks. This includes participation in the double-blind and extended active treatment part, and the follow-up period. For patients not entering Part C, the duration of the study is approximately 36 weeks.
**End of Study Definition**	The end of study is defined as the last visit of the last patient in the study.
**Population**	
**Sample Size:**	**Part A:** Approximately 80 patients (40 per treatment group). Patients may be adults ≥18 years of age or adolescents ≥12 and <18 years of age.<br>**Part B:** Approximately 210 patients (70 in each treatment group).
**Target Population:**	The target population includes adult males and females ≥18 years of age and adolescent males and females ≥12 to <18 years of age at the time of study entry with EoE. At least 10% of patients enrolled in Part B will be adolescents ≥12 to <18 years of age. At least 30% of patients enrolled in Part B must have a history of prior use of swallowed topical corticosteroids.
**Key Eligibility Criteria:**	**Key Inclusion Criteria**<br>• A documented diagnosis of EoE by endoscopic biopsy prior to screening, as demonstrated by intraepithelial eosinophilic infiltration (peak cell count ≥15 eosinophils/high power field [eos/hpf]) from at least one esophageal region and performed after at least 8 weeks of treatment with a high dose proton pump inhibitor (PPI) regimen. If the patient discontinued PPI therapy, the biopsy must have been performed within 2 weeks of the date of discontinuation.<br>If a prior (historical) endoscopic biopsy meeting these criteria is not available (or no prior biopsy is available), patients who meet other clinical and laboratory eligibility criteria will undergo treatment with a high dose PPI regimen for at least 8 weeks during the screening period before their baseline endoscopy/biopsies.<br>• Baseline endoscopic biopsies with a demonstration on central reading of intraepithelial eosinophilic infiltration (peak cell count ≥15 eos/hpf) in at least 2 of the 3 biopsied esophageal regions (proximal, mid, or distal)

	• History (by patient report) of an average of at least 2 episodes of dysphagia (with intake of solids) per week in the 4 weeks prior to screening<br>• At least 4 episodes of dysphagia in the 2 weeks prior to baseline, documented via eDiary, at least 2 of which require liquids, coughing or gagging, vomiting, or medical attention to obtain relief<br>• Completed at least 11 of 14 days of the Dysphagia Symptom Questionnaire (DSQ) eDiary data entry in the 2 weeks prior to the baseline visit (visit 3)<br>• Baseline DSQ score ≥10
	**Key Exclusion Criteria**<br>• Body weight ≤40 kg<br>• Prior participation in a dupilumab clinical trial, or past or current treatment with dupilumab<br>• Initiation or change of a food-elimination diet regimen or re-introduction of a previously eliminated food group in the 6 weeks prior to screening. Patients on a food-elimination diet must remain on the same diet throughout the study.<br>• Other causes of esophageal eosinophilia or the following conditions: hypereosinophilic syndrome and eosinophilic granulomatosis with polyangiitis (Churg-Strauss syndrome)<br>Note: Patients with eosinophilic gastroenteritis are eligible, provided they meet other eligibility criteria.<br>• Active Helicobacter pylori infection<br>• History of achalasia, Crohn's disease, ulcerative colitis, celiac disease, and prior esophageal surgery<br>• Any esophageal stricture unable to be passed with a standard, diagnostic, 9 to10 mm upper endoscope or any critical esophageal stricture that requires dilation at screening<br>• History of bleeding disorders or esophageal varices that, in the opinion of the investigator, would put the patient at undue risk for significant complications from an endoscopy procedure<br>• Treatment with swallowed topical corticosteroids within 8 weeks prior to baseline
**Treatment(s)**	
**Investigational Drug Dose/Route/Schedule:**	**Part A**<br>• Dupilumab 300 mg SC QW<br>**Part B**<br>• Dupilumab 300 mg SC QW<br>• Dupilumab 300 mg SC Q2W<br>**Part C**<br>• Dupilumab 300 mg SC QW<br>• Dupilumab 300 mg SC Q2W
**Placebo Dose/Route/Schedule:**	• Placebo SC QW (Part A and Part B only)<br>• Placebo SC Q2W alternating with Dupilumab Q2W for regimen-blinding purposes (Part B and Part C)

CONFIDENTIAL

VV-RIM-00127522-1.0 Approved - 05 Nov 2020 GMT-5:00

**Background Treatment Dose/Route/Schedule:**	For both Part A and Part B, patients who present at the initial screening visit with current use of PPIs initiated prior to screening or during the screening period must remain on the same or similar approved dosage regimen for the duration of their respective treatment period. Patients who initiate PPI therapy during the screening period must remain on the same or similar approved dosage regimen for the duration of the treatment period for their respective study part. Patients may change to a different approved PPI medication during the study. PPI therapy is prohibited for all other patients.
**Endpoint(s)**	
**Primary:**	The co-primary endpoints for both Part A and Part B are:<br>• Proportion of patients achieving peak esophageal intraepithelial eosinophil count of ≤6 eos/hpf at week 24<br>• Absolute change in DSQ score from baseline to week 24
**Key Secondary:**	The key secondary endpoints for both Part A and Part B of the study are:<br>• Absolute change in Eosinophilic Esophagitis-Endoscopic Reference Score (EoE-EREFS) from baseline to week 24<br>• Percent change in peak esophageal intraepithelial eosinophil count (eos/hpf) from baseline to week 24<br>• Absolute change in EoE Grade Score from the Histology Scoring System (EoEHSS) from baseline to week 24<br>• Absolute change in EoE Stage Score from the EoEHSS from baseline to week 24
**Other Secondary:**	Other secondary endpoints include the following:<br>• Proportion of patients achieving peak esophageal intraepithelial eosinophil count of <15 eos/hpf at week 24<br>• Proportion of patients achieving peak esophageal intraepithelial eosinophil count of ≤1 eos/hpf at week 24<br>• Percent change in DSQ from baseline to week 24<br>• Normalized Enrichment Scores (NES) for the relative change from baseline to week 24 in the EoE diagnostic panel (EDP) transcriptome signature<br>• NES for the relative change from baseline to week 24 in the type 2 inflammation transcriptome signature<br>• Absolute change from baseline to week 24 in health-related quality of life (QOL) as measured by EoE Impact Questionnaire (EoE-IQ)<br>• Absolute change from baseline to week 24 in severity and/or frequency of EoE symptoms other than dysphagia<br>• Proportion of patients who receive rescue medications or procedures during the 24-week placebo-controlled treatment period.<br>• Absolute change from baseline in esophageal distensibility plateau measured by functional lumen imaging, if collected, at week 24.<br>Note: All co-primary and secondary endpoints assessed at week 24 will be assessed at week 52 as secondary endpoints and summarized with descriptive statistics based on the treatment assignment in the double-blind treatment period as well as the extended active treatment assignment for patients previously in the placebo group.

VV-RIM-00127522-1.0 Approved - 05 Nov 2020 GMT-5:00

	Both co-primary and all secondary endpoints will be assessed at week 24 and week 52 in in adult and adolescent patients with EoE who have previously received swallowed topical corticosteroids.
**Procedures and Assessments**	**Efficacy assessments** include the following:<br>• Esophageal endoscopy with biopsies: Biopsies will be obtained for histologic assessments including intraepithelial eosinophil count and EoEHSS (EoE Grade Score and EoE Stage Score). Biopsies will be evaluated by pathologists at a central pathology laboratory who will be blinded to treatment assignment.<br>• For Part A enrolled patients, EoE-EREFS scoring will only be performed at the time of the endoscopies in Part A and Part C by the investigators who are performing the endoscopies (prior to biopsies). For Part B enrolled patients, EoE-EREFS scoring will be performed by both the investigators who perform the endoscopies in Part B and Part C as well as a centralized reading center. The centralized reading will be used for analysis, if available. If the centralized reading is not available, the EoE-EREFS performed by the investigator will be used for analysis.<br>• For all endoscopies, the investigators will assess minor esophageal features including mucosal inflammatory and remodeling features. DSQ will be completed daily by patients in the eDiary to assess the frequency and severity of dysphagia.<br>• The EoE-IQ, a disease-specific measure of health-related QOL in EoE patients, will be completed by patients at specified time points.<br>• The EoE Symptom Questionnaire, a questionnaire measuring the frequency and severity of symptoms other than dysphagia and swallowing pain, will be completed by patients at specified time points.<br>• The endolumenal functional lumen imaging probe (EndoFLIP) procedure to measure esophageal distensibility may be performed during the esophagogastroscopy procedures at selected North American sites in approximately 150 adult patients.<br>**Safety and tolerability** will be assessed by physical examinations, vital signs, electrocardiograms (ECGs), clinical laboratory tests, and clinical evaluations. Patients will be asked to report all adverse events (AEs) experienced from the time of informed consent until their last study visit.<br>**Pharmacokinetics (PK)**: Serum samples will be collected at specified time points for assay of dupilumab concentration.<br>**Anti-drug antibody (ADA)**: Serum samples will be collected at specified time points for assay of ADA and exploratory analyses.
**Statistical Plan**	**Sample Size**<br>**Part A:** The planned sample size for Part A is approximately 40 patients in each treatment group.<br>**Part B:** Based on the Part A study results, the planned sample size for Part B is approximately 70 patients in each treatment group such that for the comparison of each dupilumab dose regimen to placebo:<br>• This sample size will yield >99.9% power to detect a treatment difference of 55.4% in the proportion of patients achieving peak esophageal intraepithelial eosinophil count ≤6 eos/hpf at week 24 between placebo (5.1%) and the dupilumab treatment group (59.5%) at a 2-sided significance level of 5% using Fisher's exact test.

- This sample size will provide >99.9% power to detect a treatment difference of -12.3 points in the mean change from baseline in the total DSQ score to week 24 at a 2-sided significance level of 5% using a two-sample t-test, assuming a common standard deviation (SD) of 15.0~~13.0~~.

Therefore, the sample size of 70 patients/arm will provide an overall power of >99.9% (99.9% × 99.9%) for the co-primary endpoints, assuming no negative correlation between the two endpoints. In Part B, the same treatment effect for the two 300 mg dupilumab dose regimens (ie, QW and Q2W) is assumed.

Efficacy Analysis Sets

For Part A and Part B, the efficacy endpoints will be analyzed using the study-part-specific full analysis set (FAS) that includes all randomized patients in the corresponding study part.

The efficacy endpoints in Part C (the extended active treatment period) will be summarized for all patients who received any extended active treatment study drug in that part.

Safety Analysis Set

For Part A and Part B, the study-part-specific safety analysis set (SAF) includes all randomized patients who received any study drug in the corresponding study part; it is based on the treatment received (as treated). Treatment compliance/administration and all clinical safety variables will be analyzed using the SAF.

For safety analyses of the Part C extended treatment period, only a subset of SAF will be included, which is defined as those patients who received at least 1 dose of Part C study drug.

Statistical Analysis

All statistical analyses will be conducted separately for Part A and Part B.

Primary Efficacy Analysis (Part A and Part B):

The co-primary endpoint of proportion of patients achieving a histologic response of peak esophageal intraepithelial eosinophil count ≤6 eos/hpf at week 24 will be analyzed using the Cochran-Mantel-Haenszel (CMH) test to assess the difference in the proportion of responders in the FAS, adjusting for the randomization stratification factors.

The co-primary endpoint of absolute change from baseline in the DSQ total score at week 24 will be analyzed using an analysis of covariance (ANCOVA) model for the FAS with treatment group, randomization stratification factor, and relevant baseline measurement as covariates included in the model.

To account for use of rescue treatment in the primary analysis, for peak esophageal intraepithelial eosinophil count of ≤6 eos/hpf, patients will be considered as non-responders after the use of rescue treatment. For absolute change from baseline in the DSQ total score, data will be imputed using multiple imputation (MI) for all time points subsequent to the use of rescue treatment.

If a patient has missing value for the histological response (peak esophageal intraepithelial eosinophil count ≤6 eos/hpf) at week 24, the patient will be classified as a non-responder at week 24.

If a patient has missing value for the DSQ total score at week 24, the missing data will be imputed by MI based on similar patients who remained in the trial with observed values relevant to analysis. Similarity is based on randomized

CONFIDENTIAL

VV-RIM-00127522-1.0 Approved - 05 Nov 2020 GMT-5:00

	treatment group, baseline value, stratification factors, and available post-baseline values.<br>Sensitivity analyses will assess alternative methods to impute missing data. For the eosinophil responder analysis, sensitivity methods may include utilizing peak eosinophil values from imputations to determine responder status. Sensitivity analyses will also include an analysis without setting data post-rescue to missing.<br>***Secondary Efficacy Analysis (Part A and Part B):***<br>Secondary efficacy endpoints will be analyzed in the same fashion as the co-primary endpoints depending on the type of data, unless otherwise specified.<br>For continuous efficacy data that are scheduled to be measured only once post-baseline at week 24, missing values at week 24 will be imputed with patient's baseline value or the available post-baseline value, whichever is worse, ie, a worst observation carried forward (WOCF) approach.<br>Subgroup analysis (eg, by age group) will also be performed.<br>***Secondary Efficacy Analysis (Part C):***<br>Efficacy endpoints in Part C will be summarized with descriptive statistics by treatment received in Part C. Inferential statistics will only be provided as needed.<br>***Multiplicity Considerations:***<br>Part A and Part B will have separate and independent 2-sided alpha level of 0.05.<br>Part A: Statistical significance of the co-primary endpoints at the 2-sided Type I error rate of 5% will be required before drawing inferential conclusions about any secondary efficacy endpoints.<br>Part B: The co-primary endpoints will be tested for the comparison of dupilumab QW dose regimen to placebo first and if both endpoints are statistically significant, the testing will proceed to the co-primary endpoints of dupilumab Q2W, or the secondary endpoints of dupilumab QW, with the exact order to be specified in Part B statistical analysis plan (SAP).<br>***Safety Analysis:***<br>Safety analysis will be based on the SAF. This includes reported treatment-emergent AEs (TEAEs) and other safety data (ie, clinical laboratory evaluations, vital signs, and 12-lead ECG results). A descriptive summary of safety results will be presented by treatment group for each study part.

CONFIDENTIAL

VV-RIM-00127522-1.0 Approved - 05 Nov 2020 GMT-5:00

LIST OF ABBREVIATIONS AND DEFINITIONS OF TERMS

Ab	Antibody
ACQ-5	Asthma Control Questionnaire-5
AD	Atopic dermatitis
ADA	Anti-drug antibody
AE	Adverse event
AESI	Adverse event of special interest
ALT	Alanine aminotransferase
ANCOVA	Analysis of covariance
AST	Aspartate aminotransferase
BID	Twice (two times) a day
BUN	Blood urea nitrogen
CCL	Chemokine (C-C motif) ligand
CMH	Cochran-Mantel-Haenszel (test)
CPK	Creatine phosphokinase
CRF	Case report form
CRO	Contract research organization
CSR	Clinical study report
C_{trough}	Trough concentration
DMC	Data monitoring committee
DSQ	Dysphagia Symptom Questionnaire
EC	Ethics committee
ECG	Electrocardiogram
eCRF	Electronic case report form
EDC	Electronic data capture
EDP	EoE Diagnostic Panel
eGFR	Estimated glomerular filtration rate
EndoFLIP	Endolumenal functional lumen imaging probe
EoE	Eosinophilic esophagitis
EoE-EREFS	Eosinophilic Esophagitis-Endoscopic Reference Score
EoEHSS	EoE Histology Scoring System
EoE-IQ	EoE Impact Questionnaire
EOS	End of study (visit)
eos/hpf	Eosinophils/high power field
EOT	End of treatment
EQ-5D-3L	European Quality of Life 5-dimension (version 3L; 3-level)

ET	Early termination
EU	European Union
FAS	Full analysis set
FBR	Future biomedical research
FLG	Filaggrin
FSH	Follicle stimulating hormone
GCP	Good Clinical Practice
HBcAb	Hepatitis B core antibody
HBsAb	Hepatitis B surface antibody
HBsAg	Hepatitis B surface antigen
HCV	Hepatitis C virus
HDL	High-density lipoprotein
HIV	Human immunodeficiency virus
IA	Interim analysis
IAF	Informed assent form
ICF	Informed consent form
ICH	International Council for Harmonisation
IFN-γ	Interferon-gamma
IgE	Immunoglobulin E
IgG4	Immunoglobulin G4
IHC	Immunohistochemistry
IL	Interleukin
IL-4Rα	Interleukin-4 receptor alpha
IRB	Institutional Review Board
ISR	Injection site reaction
IVRS/IWRS	Interactive voice/web response system
LDH	Lactate dehydrogenase
LDL	Low-density lipoprotein
LOCF	Last observation carried forward
MedDRA	Medical Dictionary for Regulatory Activities
MI	Multiple imputation
MMRM	Mixed-effect model repeated measure
NES	Normalized Enrichment Scores
OIT	Oral immunotherapy

VV-RIM-00127522-1.0 Approved - 05 Nov 2020 GMT-5:00

PCSV	Potentially clinically significant value
PGIC	Patient Global Impression of Change of Dysphagia
PGIS	Patient Global Impression of Severity of Dysphagia
PK	Pharmacokinetic
POEM	Patient-Oriented Eczema Measure
PPI	Proton pump inhibitor
PPS	Per protocol set
PRO	Patient-reported outcome
PT	Preferred term (MedDRA)
PVRM	Pharmacovigilance and Risk Management
QD	Once a day
QOL	Quality of life
Q2W	Once every two weeks
QW	Once weekly
RBC	Red blood cell
RQLQ(s)+12	Rhinoconjunctivitis Quality of Life Questionnaires for 12 years and older
SAE	Serious adverse event
SAF	Safety analysis set
SAP	Statistical analysis plan
SAS	Statistical Analysis Software
SC	Subcutaneous
SCIT	Subcutaneous immunotherapy
SD	Standard deviation
SLIT	Sublingual immunotherapy
SOC	System organ class
SPRR3	Small proline-rich protein 3
SUSAR	Suspected unexpected serious adverse reaction
TARC	Thymus and activation-regulated chemokine
TB	Tuberculosis
TEAE	Treatment-emergent adverse event
Th2	Type 2 helper T cell
TNSS	Total Nasal Symptom Score
TSLP	Thymic stromal lymphopoietin
ULN	Upper limit of normal
WBC	White blood cell
WOCF	Worst observation carried forward

VV-RIM-00127522-1.0 Approved - 05 Nov 2020 GMT-5:00

TABLE OF CONTENTS

AMENDMENT HISTORY 2

CLINICAL STUDY PROTOCOL SYNOPSIS..........13

LIST OF ABBREVIATIONS AND DEFINITIONS OF TERMS..........21

1. INTRODUCTION 31

2. STUDY OBJECTIVES 33

2.1. Primary Objectives 33

2.2. Secondary Objectives 33

3. HYPOTHESIS AND RATIONALE 33

3.1. Hypothesis 33

3.2. Rationale 34

3.2.1. Rationale for Study Design..........34

3.2.2. Rationale for Dose Selection 35

3.2.3. Risk/Benefits of Participating in the Study 36

3.2.3.1. Benefits..........36

3.2.3.2. Risks 37

3.2.3.3. Benefit/Risk Conclusion..........37

4. STUDY VARIABLES..........37

4.1. Demographic and Baseline Characteristics 37

4.2. Primary and Secondary Endpoints..........38

4.2.1. Primary Endpoints 38

4.2.2. Secondary Endpoints 38

4.3. Pharmacokinetic Variables 39

4.4. Anti-Drug Antibody Variables 39

5. STUDY DESIGN 39

5.1. Study Description and Duration 39

5.1.1. Part A 42

5.1.1.1. Screening (Up to 12 Weeks)..........42

5.1.1.2. Randomization..........43

5.1.1.3. Placebo-Controlled Double-Blind Treatment Period (24 Weeks)..........43

5.1.2. Part B 44

5.1.2.1. Screening (Up to 12 Weeks)..........44

5.1.2.2. Randomization 44
5.1.2.3. Placebo-Controlled Double-Blind Treatment Period (24 Weeks) 44
5.1.3. Part C (28-Week Extended Active Treatment Period) 45
5.1.4. Follow-Up Period (12 Weeks) 45
5.1.5. End of Study Definition 45
5.2. Planned Interim Analysis 45
5.3. Study Committees 45
5.3.1. Independent Data Monitoring Committee 45
6. SELECTION, WITHDRAWAL, AND REPLACEMENT OF PATIENTS 46
6.1. Number of Patients Planned 46
6.2. Study Population 46
6.2.1. Inclusion Criteria for Parts A and B 46
6.2.2. Exclusion Criteria for Parts A and B 47
6.2.3. Exclusion Criteria for Part C (the Extended Active Treatment Period) 51
6.3. Premature Withdrawal from the Study 51
6.4. Replacement of Patient 51
7. STUDY TREATMENTS 52
7.1. Investigational and Reference Treatments 52
7.2. Background Treatment 52
7.3. Rescue Treatments 53
7.4. Dose Modification and Study Treatment Discontinuation Rules 53
7.4.1. Dose Modification 53
7.4.2. Study Drug Discontinuation 53
7.4.2.1. Reasons for Permanent Discontinuation of Study Drug 53
7.4.2.2. Reasons for Temporary Discontinuation of Study Drug 54
7.5. Method of Treatment Assignment 55
7.5.1. Blinding 55
7.5.2. Emergency Unblinding 56
7.5.3. Unblinding for Regulatory Reporting Purposes 56
7.6. Treatment Logistics and Accountability 56
7.6.1. Packaging, Labeling, and Storage 56
7.6.2. Supply and Disposition of Treatments 56

VV-RIM-00127522-1.0 Approved - 05 Nov 2020 GMT-5:00

7.6.3. Treatment Accountability 57

7.6.4. Treatment Compliance 57

7.7. Concomitant Medications and Procedures 57

7.7.1. Prohibited Medications and Procedures 57

7.7.2. Permitted Medications and Procedures 58

7.7.3. Restricted Medications and Procedures during the Follow-Up Period 58

8. STUDY SCHEDULE OF EVENTS AND PROCEDURES 59

8.1. Schedule of Events 59

8.1.1. Footnotes for the Schedule of Events Tables 66

8.1.1.1. Footnotes for Table 1 66

8.1.1.2. Footnotes for Table 2 68

8.1.1.3. Footnotes for Table 3 70

8.1.2. Early Termination Visit 71

8.1.3. Unscheduled Visits before Rescue Treatment 71

8.1.4. Unscheduled Visits for Other Reasons 71

8.2. Study Procedures 72

8.2.1. Procedures Performed Only at the Screening/Baseline Visit 72

8.2.2. Efficacy Procedures 72

8.2.2.1. Endoscopy with EoE-EREFS and Biopsies, and Photographs 72

8.2.2.2. Dysphagia Symptom Questionnaire (DSQ) - Patient-reported Outcome (PRO) 74

8.2.2.3. EoE Impact Questionnaire (EoE-IQ) 75

8.2.2.4. EoE Symptom Questionnaire 75

8.2.2.5. Patient Global Impression of Change (PGIC) of Dysphagia 75

8.2.2.6. Patient Global Impression of Severity (PGIS) of Dysphagia 76

8.2.2.7. Total Nasal Symptom Score (TNSS) 76

8.2.2.8. Standardized Rhinoconjunctivitis Quality of Life Questionnaire for ages 12+ [RQLQ(S)+12] 76

8.2.2.9. Juniper Asthma Control Questionnaire (ACQ) 77

8.2.2.10. Patient-Oriented Eczema Measure (POEM) 77

8.2.2.11. European Quality of Life 5-Dimensional Scale (EQ-5D) 78

8.2.3. Safety Procedures 78

8.2.3.1. Vital Signs 78

VV-RIM-00127522-1.0 Approved - 05 Nov 2020 GMT-5:00

8.2.3.2. Physical Examination 78
8.2.3.3. Body Weight and Height 78
8.2.3.4. Electrocardiogram 78
8.2.3.5. Laboratory Testing 78
8.2.4. Drug Concentration and Measurements 80
8.2.5. Anti-Drug Antibody Measurements and Samples 80
8.2.6. Pharmacodynamic and Exploratory Biomarker Procedures 80
8.2.6.1. EoE Diagnostic Panel and Type 2 Inflammation Transcriptomics 80
8.2.6.2. Type 2 Inflammatory and Disease-Related Circulating Biomarkers 81
8.2.7. Future Biomedical Research (FBR) Serum/Plasma (Optional) 82
8.2.7.1. Genomics Substudy (Optional) 82
9. SAFETY DEFINITIONS, REPORTING, AND MONITORING 83
9.1. Obligations of Investigator 83
9.2. Obligations of Sponsor 83
9.3. Definitions 83
9.3.1. Adverse Event 83
9.3.2. Serious Adverse Event 83
9.3.3. Adverse Events of Special Interest 84
9.4. Recording and Reporting Adverse Events 84
9.4.1. Adverse Events 84
9.4.2. Serious Adverse Events 84
9.4.3. Other Events that Require Accelerated Reporting to Sponsor 85
9.4.4. Reporting Adverse Events Leading to Withdrawal from the Study 86
9.4.5. Abnormal Laboratory, Vital Signs, or Electrocardiogram Results 86
9.4.6. Follow-up 86
9.5. Evaluation of Severity and Causality 86
9.5.1. Evaluation of Severity 86
9.5.2. Evaluation of Causality 87
9.6. Safety Monitoring 88
9.7. Investigator Alert Notification 89
10. STATISTICAL PLAN 89
10.1. Statistical Hypothesis 89

CONFIDENTIAL

VV-RIM-00127522-1.0 Approved - 05 Nov 2020 GMT-5:00

10.2. Justification of Sample Size....89
10.3. Analysis Sets....91
10.3.1. Efficacy Analysis Sets....91
10.3.2. Safety Analysis Set....91
10.3.3. Pharmacokinetic Analysis Sets....91
10.3.4. Anti-Drug Antibody Analysis Sets....91
10.4. Statistical Methods....91
10.4.1. Patient Disposition....92
10.4.2. Demography and Baseline Characteristics....92
10.4.3. Efficacy Analyses....92
10.4.3.1. Primary Efficacy Analysis....92
10.4.3.2. Secondary Efficacy Analysis....94
10.4.3.3. Multiplicity Considerations....96
10.4.4. Safety Analysis....97
10.4.4.1. Adverse Events....97
10.4.4.2. Other Safety....98
10.4.4.3. Treatment Exposure....98
10.4.4.4. Treatment Compliance....99
10.4.5. Pharmacokinetics....99
10.4.5.1. Analysis of Drug Concentration Data....99
10.4.6. Analysis of Anti-Drug Antibody Data....99
10.4.7. Analysis of Pharmacodynamic and Exploratory Biomarker Data....99
10.4.8. Analysis of Quality of Life Data....99
10.4.9. Timing of Statistical Analysis....99
10.5. Additional Statistical Data Handling Conventions....100
10.6. Statistical Considerations Surrounding the Premature Termination of a Study....100
11. DATA MANAGEMENT AND ELECTRONIC SYSTEMS....100
11.1. Data Management....100
11.2. Electronic Systems....101
12. STUDY MONITORING....101
12.1. Monitoring of Study Sites....101
12.2. Source Document Requirements....101

CONFIDENTIAL

VV-RIM-00127522-1.0 Approved - 05 Nov 2020 GMT-5:00

12.3. Case Report Form Requirements 102
13. AUDITS AND INSPECTIONS 102
14. ETHICAL AND REGULATORY CONSIDERATIONS 102
14.1. Good Clinical Practice Statement 102
14.2. Informed Consent 103
14.3. Patients Confidentiality and Data Protection 103
14.4. Institutional Review Board/Ethics Committee 104
15. PROTOCOL AMENDMENTS 104
16. PREMATURE TERMINATION OF THE STUDY OR CLOSE-OUT OF A SITE 104
16.1. Premature Termination of the Study 104
16.2. Close-out of a Site 105
17. STUDY DOCUMENTATION 105
17.1. Certification of Accuracy of Data 105
17.2. Retention of Records 105
18. DATA QUALITY ASSURANCE 106
19. CONFIDENTIALITY 106
20. FINANCING AND INSURANCE 106
21. PUBLICATION POLICY 107
22. REFERENCES 107
23. INVESTIGATOR’S AGREEMENT 112
SIGNATURE OF SPONSOR’S RESPONSIBLE OFFICERS 113

LIST OF TABLES

Table 1: Parts A and B Schedule of Events – Screening Period and Double-Blind Treatment Period 60
Table 2: Part C Schedule of Events – Extended Active Treatment Period 62
Table 3: Follow-up Period, Early Termination Visit, and Unscheduled Visit 64
Table 4: Summary of Primary Estimand for Co-primary Endpoints 93
Table 5: Summary of Primary Estimand for Key Secondary Endpoints 95

VV-RIM-00127522-1.0 Approved - 05 Nov 2020 GMT-5:00

LIST OF FIGURES

Figure 1: Study Flow Diagram 40
Figure 2: Study Design 41
Figure 3: Endoscopy/Biopsy Procedure Flow Diagram 43

VV-RIM-00127522-1.0 Approved - 05 Nov 2020 GMT-5:00

1. INTRODUCTION

Eosinophilic esophagitis (EoE) is a chronic, inflammatory, allergic/immune-mediated disease of the esophagus. The disease is characterized by local eosinophilic inflammation leading to symptoms of esophageal dysfunction (Furuta, 2017) (Liacouras, 2011) (DeBrosse, 2011) (Falk, 2014). Although considered a rare disease, the current prevalence is estimated at 22.7 per 100,000 worldwide (Arias, 2016) and appears to be on the increase (Dellon, 2014a). Eosinophilic esophagitis has been reported in all ages; however, most cases are in children and adults younger than 50 years (Dellon, 2014a) (Dellon, 2014b) (Dellon, 2007) (Kapel, 2008) (Liacouras, 2011) (Spergel, 2009). Gender differences in EoE have been consistently reported, with males affected 3 to 4 times more often than females (Dellon, 2014a), but there are no gender-related differences in the clinical symptoms (Kapel, 2008) (Prasad, 2009) (Veerappan, 2009) (Hruz, 2011) (Ally, 2015).

The primary clinical manifestations of EoE in both adults and children over 10 years of age are dysphagia and food impaction (Lucendo, 2017). These symptoms lead to substantially impaired quality of life (QOL) (DeBrosse, 2011) (Falk, 2014) (Straumann, 2008) (Straumann, 2003). Endoscopic findings are related to the inflammation in the esophagus and consist of fixed or transient concentric rings, longitudinal furrows, white plaques, reduced mucosal vascularity, fragile or crepe-like mucosa, and strictures. Rings and furrows are the most common, seen in nearly half of patients (Singla, 2016).

Growing evidence suggests that a Type 2 cytokine-mediated immune response plays an important role in the development of EoE. This is thought to occur by provoking chronic eosinophil, mast cell, T cell, and lymphocyte-induced inflammation via cytokines known to regulate eosinophilic accumulation in the esophagus, such as interleukin (IL)-4, IL-5, IL-13, and eotaxin-1, -2, and -3 (Abonia, 2010) (Abonia, 2012) (Blanchard, 2006) (Blanchard, 2008) (Blanchard, 2010) (Bullock, 2007) (Mishra, 2003) (Mishra, 2009`) (Straumann, 2001). Th2 cells are a subgroup of the T cells that regulate the activity of other immune cells by releasing Type 2 cytokines. IL-13 has been found to be upregulated in the esophageal epithelium of EoE patients (Blanchard, 2007). The induction of eotaxin-3, an eosinophil chemoattractant, is thought to be an important factor in EoE pathogenesis (Blanchard, 2006) (Blanchard, 2007). The 2 most up-regulated genes in esophageal biopsies from EoE patients (compared to normal controls) encode eotaxin-3 and periostin, another protein induced by Type 2 cytokines and thought to promote inflammation and remodeling (Blanchard, 2006) (Blanchard, 2008). Furthermore, esophageal biopsies and blood samples of patients with active EoE have increased levels of cytokines and chemokines associated with Type 2 inflammation, including IL-4 and IL-13, thymic and stromal lymphopoeitin (TSLP), and eotaxin-3 secreted by the typical cells involved in allergic inflammation: T cells, mast cells, basophils, invariant natural killer T cells (iNKTs), and esophageal epithelial cells. Mutations in the eotaxin-3 and TSLP genes, whose functions propagate Type 2 inflammation, have been associated with EoE risk (Cianferoni, 2016).

Impaired barrier function of the esophageal epithelium is thought to play a role in EoE pathogenesis. Several genes in the epidermal differentiation complex, known to be down-regulated by Type 2 cytokines with resultant barrier dysfunction of the skin, are also down-regulated in primary esophageal epithelial cells in vitro. Furthermore, decreased mRNA expression of the epidermal differentiation complex gene filaggrin (FLG), and the esophagus-specific esophagin

VV-RIM-00127522-1.0 Approved - 05 Nov 2020 GMT-5:00

(small proline-rich protein 3 [SPRR3]), was observed in esophageal biopsies from EoE patients compared to normal controls (Blanchard, 2010). The increased frequency at which FLG null alleles were found in EoE patients compared to controls provides further evidence of the barrier hypothesis in EoE (Blanchard, 2010). It is also known that FLG mRNA expression is directly suppressed by IL-4 and IL-13 in keratinocytes (Howell, 2007). The data suggest that Type 2 cytokines may be driving not only an inflammatory response, but also epithelial barrier dysfunction in the esophagus.

Consistent with the Type 2-mediated inflammation observed in esophageal tissue, patients with EoE have high rates of comorbid allergic diseases which are also associated with enhanced IL-4 and IL-13 signaling, especially food allergies, atopic dermatitis (AD), asthma, and allergic rhinitis (Arora, 2012) (Assa'ad, 2008) (Liacouras, 2011) (Moawad, 2010) (Roy-Ghanta, 2008) (Weinbrand-Goichberg, 2013).

The inflammatory damage to the esophageal epithelium results in symptoms of esophageal dysfunction, such as dysphagia. Chronic inflammation of the esophagus may also lead to remodeling, stricture formation, and fibrosis (Hirano, 2014). The fibrotic aspect of progressed disease is not well understood, and whether or not this can be reversed with treatment is unknown.

Current therapeutic approaches include chronic dietary elimination, swallowed topical formulation corticosteroids (not approved for the treatment of EoE outside the European Union [EU]), and esophageal dilation. Emergency endoscopy for prolonged and/or painful food impaction is associated with a risk of severe esophageal injury and does not alter the underlying pathogenesis or progression of the disease. Although swallowed topical corticosteroids have been reported in clinical trials to induce partial clinical responses and histologic remission, they are not uniformly effective and can be associated with fungal infections as well as disease recurrence after discontinuation.

Dupilumab is a human monoclonal immunoglobulin G4 (IgG4) antibody (Ab) that inhibits IL-4 and IL-13 signaling by specifically binding to the IL-4 receptor alpha (IL-4Rα) subunit shared by the IL-4 and IL-13 receptor complexes. Blocking IL-4Rα with dupilumab inhibits IL-4 and IL-13 Type 2 cytokine-induced responses, including the release of pro-inflammatory cytokines, chemokines, and IgE. Additionally, preclinical data demonstrate that treatment with dupilumab prevents infiltration of eosinophils into tissues. For these reasons, dupilumab was evaluated in adult patients with EoE in a phase 2, multicenter, double-blind, randomized, placebo-controlled study (R668-EE-1324), where it demonstrated substantial improvements in clinical, histologic, and endoscopic aspects of the disease. Dupilumab was well tolerated in the study, with safety data generally consistent with other dupilumab studies in AD patients and no particular safety signal associated with use in the EoE patient population. The results support the phase 3 evaluation of dupilumab in EoE.

Additional background information on the study drug and development program can be found in the Investigator's Brochure.

VV-RIM-00127522-1.0 Approved - 05 Nov 2020 GMT-5:00

2. STUDY OBJECTIVES

2.1. Primary Objectives

The primary objectives of the study by study part are:

- ***Part A:*** To determine the treatment effect of dupilumab compared with placebo in adult and adolescent patients with EoE after 24 weeks of treatment as assessed by histological and clinical measures, and to inform/confirm the final sample size determination for Part B.
- ***Part B:*** To demonstrate the efficacy of dupilumab treatment compared with placebo in adult and adolescent patients with EoE after 24 weeks of treatment as assessed by histological and clinical measures.
- ***Part C:*** To assess the safety and efficacy of dupilumab treatment in adult and adolescent patients with EoE after up to 52 weeks of treatment as assessed by histological and clinical measures.

2.2. Secondary Objectives

The secondary objectives of the study are:

- To evaluate the safety, tolerability, and immunogenicity of dupilumab treatment for up to 52 weeks in adult and adolescent patients with EoE
- To explore the relationship between dupilumab concentration and responses in adult and adolescent patients with EoE, using descriptive analyses
- To evaluate the effects of dupilumab on transcriptomic signatures associated with EoE and type 2 inflammation
- To demonstrate the efficacy of dupilumab treatment compared to placebo after 24 weeks and 52 weeks of treatment in adult and adolescent patients with EoE who have previously received swallowed topical corticosteroids

3. HYPOTHESIS AND RATIONALE

3.1. Hypothesis

In adult and adolescent patients ≥12 years of age with EoE, treatment with dupilumab will result in a statistically significant benefit compared to treatment with placebo, as measured by:

- Relief of dysphagia, assessed by change in the Dysphagia Symptom Questionnaire (DSQ) score.
- Reduction in eosinophilic esophageal infiltration, assessed by change in esophageal intraepithelial eosinophil count
- Improvement in endoscopically identified esophageal mucosal inflammatory and remodeling features, assessed by change in the Eosinophilic Esophagitis-Endoscopic Reference Score (EoE-EREFS)

VV-RIM-00127522-1.0 Approved - 05 Nov 2020 GMT-5:00

3.2. Rationale

3.2.1. Rationale for Study Design

The study is divided into 3 parts:

- Part A consists of a screening period of up to 12 weeks, followed by a randomized, double-blind, placebo-controlled, 2-arm, parallel-group, 24-week treatment period. Patients who complete Part A will either enter Part C or continue to a 12-week follow-up period (for patients who do not go on to Part C).
- Part B consists of a screening period of up to 12 weeks, followed by a randomized, double-blind, placebo-controlled, 3-arm, parallel-group, 24-week treatment period. Patients who complete Part B will either enter Part C or continue to a 12-week follow-up period (for patients who do not go on to Part C). Enrollment in Part B will begin immediately after the last patient has been randomized in Part A.
- Part C is an extended active treatment period and consists of a 2-arm, 28-week treatment period. Patients who complete Part C will continue to a 12-week follow-up period.

The co-primary endpoints selected for Parts A and B of the study (proportion of patients achieving peak esophageal intraepithelial eosinophil count of ≤6 eos/high power field [hpf] at week 24 and change in DSQ score from baseline to week 24) assess important clinical and histological measures of dysphagia symptoms and disease activity and progression. Eosinophilic infiltration of the esophageal mucosa is the hallmark of the disease and, in conjunction with characteristic clinical symptoms of esophageal dysfunction (solid food dysphagia, food impaction), is considered pathognomonic for EoE.

Solid food dysphagia is the most common presenting symptom of EoE and food impaction necessitating endoscopic bolus removal occurs in 33% to 54% of adult EoE patients (Dellon, 2013). The DSQ is a daily patient-reported outcome (PRO) measure that has been validated for the measurement of dysphagia frequency and severity in patients with EoE (Dellon, 2013) (Hudgens, 2017). The questions ask whether solid food has been eaten; whether food has gone down slowly or become stuck; and what, if any, measures have been taken to achieve relief.

Since the gross appearance of the esophageal mucosa assessed by endoscopy reflects both the inflammatory and fibrotic nature of the disease, EoE-EREFS is a key secondary endpoint. The EoE-EREFS is a validated classification and grading system designed to standardize nomenclature for the major endoscopically identified esophageal features of EoE (edema, rings, exudates, furrows, and strictures) (Hirano, 2013). Recent studies have demonstrated the clinical relevance of endoscopic severity assessment in EoE. The severity of each of the EoE-EREFS subscores has been shown to be associated with patient-reported global symptom activity (Schoepfer, 2014). The use of a visual endoscopy score is consistent with the emphasis on endoscopic mucosal healing as a primary endpoint of therapeutics in inflammatory bowel disease, peptide ulcer disease and GERD (Hirano, 2017).

CONFIDENTIAL

VV-RIM-00127522-1.0 Approved - 05 Nov 2020 GMT-5:00

The study will include adolescents ≥12 years of age. The primary clinical manifestation of EoE in children and adolescents ≥10 years of age is dysphagia and food impaction, similar to adult patients (Lucendo, 2017). In addition, the disease appears to have the same underlying pathophysiology regardless of age; similar high rates of atopy as well as a genetic predisposition have been demonstrated in both the pediatric and adult populations. A unique genetic signature for EoE has been identified that is conserved in all patients with the disease, suggesting the biological manifestations of disease are the same across all age groups (Blanchard, 2006). Additional work demonstrated that a transcriptome of 96 genes in EoE patients was similar between both adults and children (Wen, 2013).

Study treatment will be administered for approximately 24 weeks in the double-blind, placebo-controlled phase to adequately assess the risk/benefit profile of a drug intended for chronic use. In the phase 2, multicenter, double-blind, randomized, placebo-controlled study in patients with EoE (R668-EE-1324), a plateau in clinical effect had not been observed by week 12 with a dose of 300 mg once weekly (QW). An additional 28 weeks of extended active treatment in Part C for an overall exposure of approximately 52 weeks will provide data on the long-term efficacy and safety of dupilumab in patients with EoE.

There are several justifications for the inclusion of a placebo control group in this study. Although there is an approved swallowed topical steroid treatment for EoE in the EU, it is only indicated for short-term use in adults, not for chronic treatment or treatment of pediatric patients (EMA Jorveza Assessment Report, 2017). Furthermore, it is only approved in this region and thus considered inappropriate for use in a global study. Studies show that patients with EoE frequently relapse, even while maintained on long-term topical steroid therapy (Eluri, 2017). Furthermore, although studies have shown that a prolonged delay in the diagnosis and treatment of EoE increases the risk of stricture formation in a time-dependent manner, the median delay in EoE diagnosis in these studies was 6 years, and duration of follow-up was over 20 years (Schoepfer, 2013). It is unlikely that a 24-week period of untreated disease will lead to irreversible stricture formation. Hence, while allocation to a placebo arm may result in temporary continuation of symptoms, it should not impact a patient's long-term prognosis. In addition, rescue treatment (including swallowed steroids and dilation) is allowed for any patient who experiences intolerable symptoms during the study. Finally, study patients will be offered the opportunity to participate in the 28-week extended active treatment phase, thus offering some potential for direct benefit, even for patients initially enrolled in the placebo treatment arm.

3.2.2. Rationale for Dose Selection

Patients in Part A will receive either 300 mg dupilumab or placebo QW administered subcutaneously (SC). In the phase 2 study R668-EE-1324, dupilumab 300 mg QW was demonstrated to be efficacious with clinically significant improvements in histologic, endoscopic, and patient-reported symptoms after 12 weeks of treatment. Dupilumab was well tolerated with a favorable profile with no particular safety signal associated with use in the EoE patient population.

Patients in Part B will receive 300 mg dupilumab SC once every two weeks (Q2W), 300 mg dupilumab SC QW or placebo SC QW. The 300 mg Q2W was chosen to provide dose-ranging information. Based on pharmacokinetic (PK) considerations, this dose is expected to achieve target-saturating concentrations over the dosing interval, and hence provide clinical benefit. The approved dose regimen for treatment of AD is 300 mg Q2W. In addition, this dose regimen

VV-RIM-00127522-1.0 Approved - 05 Nov 2020 GMT-5:00

provided significant benefit in phase 3 studies of dupilumab for the treatment of adults and adolescents with moderate-to-severe asthma, and is currently being tested in adults with nasal polyposis. Dosing regimens with inter-dose intervals longer than Q2W have been suboptimal in AD and asthma and are not proposed for this trial, as the end of interval concentrations would be expected to be below those required for target saturation, and hence may not yield optimal benefit in patients with EoE.

Both dupilumab 300 mg QW and Q2W have a well understood and favorable safety profile. Approximately 2500 AD patients were exposed to dupilumab; 645 AD patients have been exposed to 300 mg QW for ≥364 days, and 58 have been exposed to 300 mg Q2W for ≥364 days as of the 27 April 2016 biologics license application cut-off. In completed/unblinded Phase 2/3 studies of dupilumab in asthma patients (as of 30 September 2017), 2649 asthma patients were exposed to dupilumab; 1035 of these patients have been exposed to 300 mg Q2W for ≥1 year, 662 have been exposed for ≥1.5 years, and 473 have been exposed for ≥2 years.

In both the AD and asthma programs, as is typical for monoclonal antibodies, body weight was found to be the most significant covariate affecting the PK of dupilumab, with patients of a lower body weight achieving higher concentrations of dupilumab. In the pivotal phase 3 studies in AD (R668-AD-1334, R668-AD-1416, and R668-AD-1224), adults with body weights of 41 kg or more received dupilumab doses of 300 mg Q2W and 300 mg QW, which were found to be well tolerated for 16 to 52 weeks. In Study EFC13579, 33 adolescent patients with asthma (median age of 14 years with a range of 12 to 17 years; median weight 55 kg) received dupilumab 300 mg Q2W; a similar PK profile was observed between adolescent and adult patients with asthma.

As dupilumab 300 mg QW has not been studied in lighter patients, subjects (adults or adolescents) with body weight <40 kg will be excluded from the study.

3.2.3. Risk/Benefits of Participating in the Study

Recognizing that the "Coronavirus Disease 2019" (COVID-19) pandemic will have an impact on the conduct of clinical trials, the sponsor does not intend to screen any new patients in this study unless the impact of the COVID-19 pandemic is deemed manageable and no longer interfering with the conduct of trials at individual sites, and patients can safely participate in this study. Until then, the sponsor plans to obtain approvals from health authorities/ethics committees to enable continuation of study sites for this study, as allowed by local laws and regulations.

3.2.3.1. Benefits

The efficacy and safety of dupilumab in adults with EoE were evaluated in a phase 2, randomized, double-blind, placebo-controlled study (R668-EE-1324). Treatment with weekly dupilumab 300 mg SC for 12 weeks in patients with EoE reduced the frequency and intensity of dysphagia, the cardinal symptom of EoE. Improvement in clinical symptoms was accompanied by endoscopic and biopsy histologic evidence of reduced esophageal intraepithelial eosinophil infiltration, esophageal mucosal inflammation, and improved esophageal distensibility. Study patients could potentially benefit from dupilumab treatment, including patients randomized to receive placebo in Parts A and B, as all patients have the opportunity to receive dupilumab in the extended active treatment Part C of the study.

3.2.3.2. Risks

As of 28 March 2020, over 8000 subjects have been enrolled into the dupilumab development program for the indications of AD, asthma, chromic rhinosinusitis with nasal polyps (CRSwNP), grass allergy, peanut allergy, bullous pemphigoid, and EoE (Investigator's Brochure Version 14).

Based upon currently available data, 1 important identified risk, “Systemic Hypersensitivity (including those events associated with immunogenicity),” has been established. One serious case each of serum sickness and serum sickness-like reaction (both associated with high anti-drug antibody [ADA] titers) were reported in adult AD studies, however none were seen in the asthma development program. Additionally, there has been one report of anaphylaxis considered related to study drug by the Investigator in an asthma study. Based on the review of post-marketing data, signals of anaphylactic reaction and angioedema were confirmed and considered as adverse drug reactions (ADRs).

A higher incidence of Injection Site reactions (ISRs) has been observed in the dupilumab-treated subjects, consistent with the SC injection of a protein biologic. Most ISRs were mild to moderate in intensity, and less than 2% were severe or led to treatment discontinuation. The proportion of patients experiencing ISRs diminished over time during the treatment period.

Conjunctivitis, Conjunctivitis Allergic, Conjunctivitis Bacterial, Blepharitis, Dry Eye, Eye Pruritus, Herpes Simplex (primarily mucocutaneous in nature), and Oral Herpes have been identified as adverse drug reactions for the AD indication. There was no increase in incidence of these events observed in the completed asthma study. Conjunctivitis was also observed in the completed CRSwNP studies but with lower incidence than in the AD studies and without keratitis or increase in other ophthalmic events. Most events were mild in intensity and transient in nature, and did not necessitate treatment discontinuation. Based on the post-marketing safety data, events of angioedema, arthralgia, keratitis and ulcerative keratitis are now considered as ADRs.

The purpose of protocol amendment 4 was to account for the COVID-19 pandemic and to minimize the risks to the patients in the trial as well as healthcare providers by allowing flexibility in the visit schedule while social distancing suggestions are in place. Allowing for this flexibility does not increase the risk of participating in this trial as there will be continued contact between the patients and study personnel despite postponement of in-person clinic visits.

3.2.3.3. Benefit/Risk Conclusion

The safety data available to date across multiple indications in conjunction with the clinical benefit of dupilumab demonstrated in the phase 2 EoE study support a favorable benefit-risk profile for dupilumab.

4. STUDY VARIABLES

4.1. Demographic and Baseline Characteristics

Baseline characteristics will include standard demography (eg, age, race, weight, height, etc.), disease characteristics including medical history, and medication history.

4.2. Primary and Secondary Endpoints

4.2.1. Primary Endpoints

The co-primary endpoints for both Parts A and B of the study are:

- Proportion of patients achieving peak esophageal intraepithelial eosinophil count of ≤6 eos/hpf at week 24
- Absolute change in DSQ score from baseline to week 24

4.2.2. Secondary Endpoints

The key secondary endpoints for both Parts A and B of the study are:

- Absolute change in EoE-EREFS from baseline to week 24
- Percent change in peak esophageal intraepithelial eosinophil count (eos/hpf) from baseline to week 24
- Absolute change in EoE Grade Score from the EoE Histology Scoring System (EoEHSS) from baseline to week 24
- Absolute change in EoE Stage Score from the EoEHSS from baseline to week 24

The other secondary endpoints are:

- Proportion of patients achieving peak esophageal intraepithelial eosinophil count of <15 eos/hpf at week 24
- Proportion of patients achieving peak esophageal intraepithelial eosinophil count of ≤1 eos/hpf at week 24
- Percent change in DSQ from baseline to week 24
- Normalized Enrichment Scores (NES) for the relative change from baseline to week 24 in the EoE diagnostic panel (EDP) transcriptome signature
- NES for the relative change from baseline to week 24 in the type 2 inflammation transcriptome signature
- Absolute change from baseline to week 24 in health-related QOL as measured by EoE Impact Questionnaire (EoE-IQ)
- Absolute change from baseline to week 24 in severity and/or frequency of EoE symptoms other than dysphagia
- Proportion of patients who receive rescue medications or procedures during the 24-week placebo-controlled treatment period
- Absolute change from baseline in esophageal distensibility plateau measured by functional lumen imaging, if collected, at week 24.

<u>Note</u>: All the above co-primary and secondary endpoints assessed at week 24 will be assessed at week 52 as secondary endpoints and summarized with descriptive statistics

VV-RIM-00127522-1.0 Approved - 05 Nov 2020 GMT-5:00

based on the treatment assignment in the double-blind treatment period as well as the extended active treatment assignment for patients previously in the placebo group.

Both co-primary and all secondary endpoints will be assessed at week 24 and week 52 in in adult and adolescent patients with EoE who have previously received swallowed topical corticosteroids.

4.3. Pharmacokinetic Variables

The pharmacokinetic variable is the trough concentration (C_{trough}) of functional dupilumab in serum at each time point. Samples in this study will be collected using a sparse sampling schedule, eg, only 1 blood sample for drug concentration measurement is collected at any single clinic visit. These sampling time points (prior to dosing at each time point) are specified in Table 1, Table 2, and Table 3.

4.4. Anti-Drug Antibody Variables

The ADA variables are ADA status, titer, and time point/visit. Samples in this study will be collected at the clinic visits specified in Table 1, Table 2, and Table 3.

5. STUDY DESIGN

5.1. Study Description and Duration

This study will investigate the efficacy and safety of dupilumab in adult and adolescent patients with EoE. The study will consist of 3 parts and a follow-up period: Part A and Part B (each consisting of a 24-week double-blind treatment period), Part C (a 28-week extended active treatment period), and a 12-week follow-up period following the end of the extended active treatment period for patients who enter into Part C or following the end of Parts A and B of the double-blind treatment period for patients who do not enter Part C.

Approximately 80 patients (40 per treatment group) are planned to be enrolled in Part A. Approximately 210 patients (70 per treatment group) are planned to be enrolled in Part B. At least 10% of patients enrolled in Part B will be adolescents ≥12 years of age and at least 30% of patients enrolled in Part B must have a history of prior use of swallowed topical corticosteroids. At the end of the double-blind treatment visit (week 24) of Part A or Part B, eligible patients may enter Part C.

The study flow diagram is provided in Figure 1.

VV-RIM-00127522-1.0 Approved - 05 Nov 2020 GMT-5:00

Figure 1: Study Flow Diagram

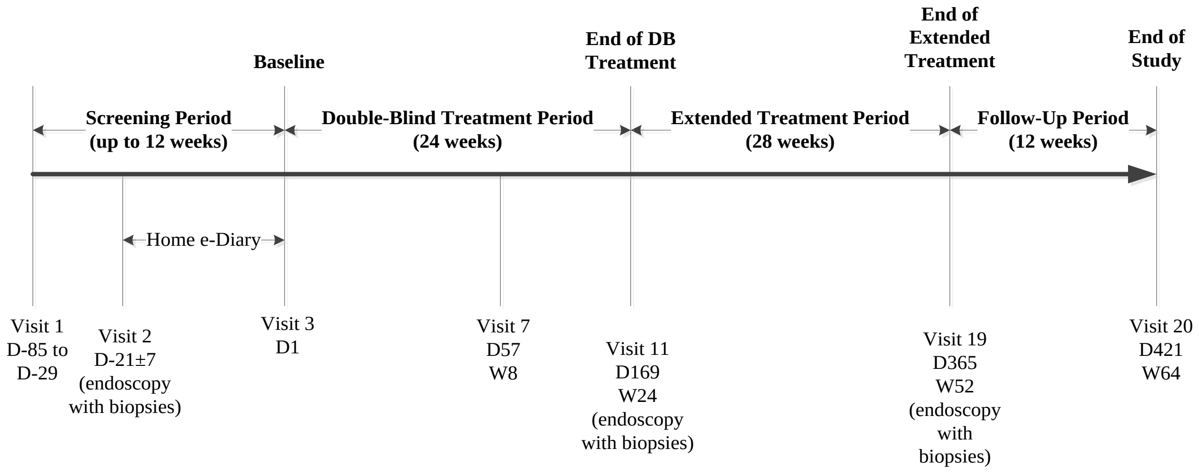

DB = double-blind.
Note: For patients who do not have at least 11 daily entries during the 14 days immediately preceding the planned randomization date (baseline), randomization should be postponed until this requirement is met, but without exceeding the 85-day maximum duration for screening.

This study consists of 3 parts: Part A and Part B are 24-week treatment, randomized, double-blind, placebo-controlled study phases, and Part C is a 28-week, extended active treatment phase that will enroll patients from Part A and Part B. A depiction of the randomization scheme for this study is below in Figure 2 .

VV-RIM-00127522-1.0 Approved - 05 Nov 2020 GMT-5:00

Figure 2: Study Design

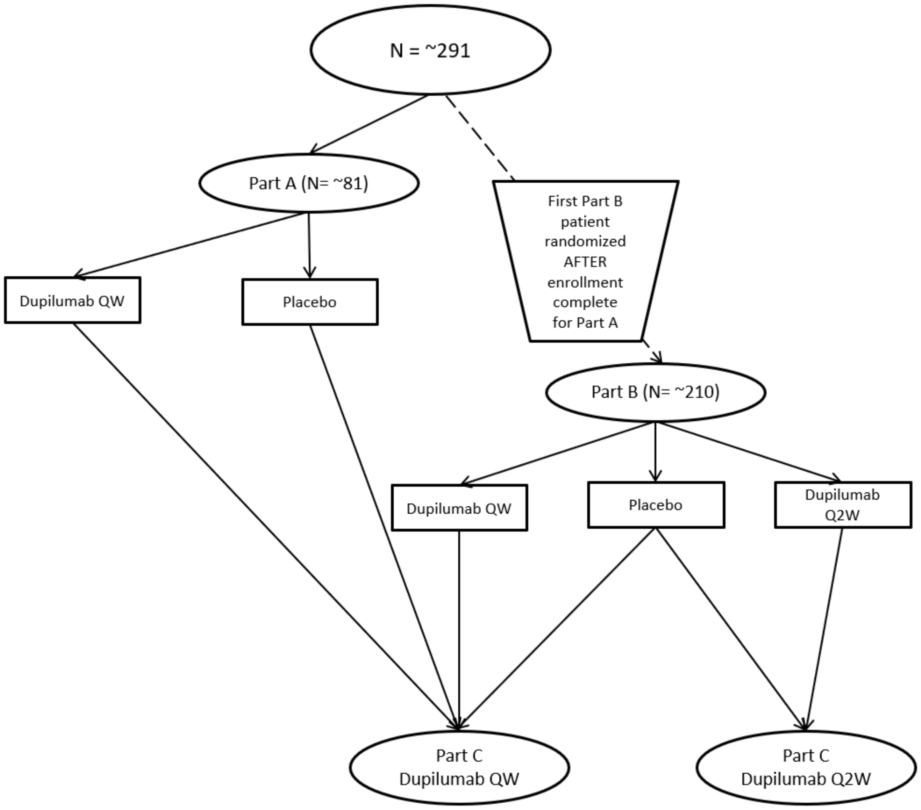

Note: If there are restrictions to the clinical study as a result of the COVID-19 pandemic, it may be necessary to adjust the visit schedule, convert in-person visits to telephone contacts, and postpone study procedures until the next available in clinic study visit. It is necessary that the randomization visit (V3) and the first visit of Part C (V11) occur in the clinic. Endoscopies with biopsies are required at approximately week 24 and week 52. If it is not possible to complete the endoscopies with biopsies due to COVID-19 restrictions and provided there are no specific safety concerns for the patient, patients may continue their current study medication regimen until the endoscopies with biopsies can be performed. All temporary mechanisms utilized, and deviations from planned study procedures are to be documented as being related to COVID-19 and will remain in effect only for the duration of the public health emergency. Once COVID-19 conditions resolve, all study visits and procedures should follow the schedule of events as specified in Table 1, Table 2, and Table 3.

VV-RIM-00127522-1.0 Approved - 05 Nov 2020 GMT-5:00

5.1.1. Part A

5.1.1.1. Screening (Up to 12 Weeks)

After adult patients provide informed consent and adolescent patients and/or their legal parents/legal guardians provide informed consent and informed assent (as appropriate), patients will be assessed for study eligibility at visit 1.

Study participants are required to have a confirmed diagnosis of EoE which may be established *either* by a prior historical biopsy showing ≥15 intraepithelial eosinophils per high-power field (eos/hpf) from at least one esophageal region after at least 8 weeks of treatment with a high-dose proton pump inhibitor (PPI) using any approved PPI *or* by biopsies performed after approximately 8 weeks of high-dose PPI treatment initiated prior to screening or during the screening period, which demonstrate ≥15 intraepithelial eos/hpf in at least 2 out of 3 esophageal regions (proximal, mid, and distal); see Figure 3 for endoscopy/biopsy procedure flow chart. Patients who are on PPIs during the screening period and are eligible to enroll in the study must continue a high-dose PPI regimen during the study (see details in Section 7.2). Patients are allowed to switch among the approved background therapy options for high-dose PPI use during the study.

All patients who meet the other clinical and laboratory eligibility criteria will undergo endoscopy with biopsies at visit 2 (day -21±7) to establish a baseline reference measure. For patients without a historical biopsy, the visit 2 biopsy will serve as both confirmation of EoE diagnosis and the baseline reference measure.

All biopsies performed during this study will be evaluated by pathologists at a central pathology laboratory who will be blinded to treatment assignment.

Note: Biopsy specimens from the stomach and/or duodenum will be obtained in all patients <18 years of age to rule out alternate etiologies of esophageal eosinophilia. Targeted, stomach, and/or duodenal biopsies will be obtained in adults only in the event of abnormal endoscopic findings (other than typical EoE findings) or clinical suspicion of alternate etiologies. All randomized patients found to have stomach and/or duodenal abnormalities at baseline will have follow-up stomach and/or duodenal biopsies at week 24 and week 52.

After confirmation of EoE diagnosis, patients will be given an electronic diary (eDiary) to record dysphagia symptoms on a daily basis during the 2 weeks prior to the baseline visit (visit 3).

Patients may be re-screened once if they fail the screening evaluation, unless the reason for screen failure is related to histologic or clinical disease severity inclusion criteria. The baseline endoscopy with biopsy and EoE-EREFS scoring will not be repeated for re-screened patients. These results will continue to be valid baseline data. Re-screening must occur within 6 months of the screen failure.

For patients whose DSQ diary compliance does not meet eligibility requirements within the 21-day interval between Visit 2 and Visit 3 (at least 11 daily entries during the 14 days immediately preceding the planned randomization date), randomization may be postponed as long as the total duration of the screening period does not exceed the 85-day maximum.

VV-RIM-00127522-1.0 Approved - 05 Nov 2020 GMT-5:00

Figure 3: Endoscopy/Biopsy Procedure Flow Diagram

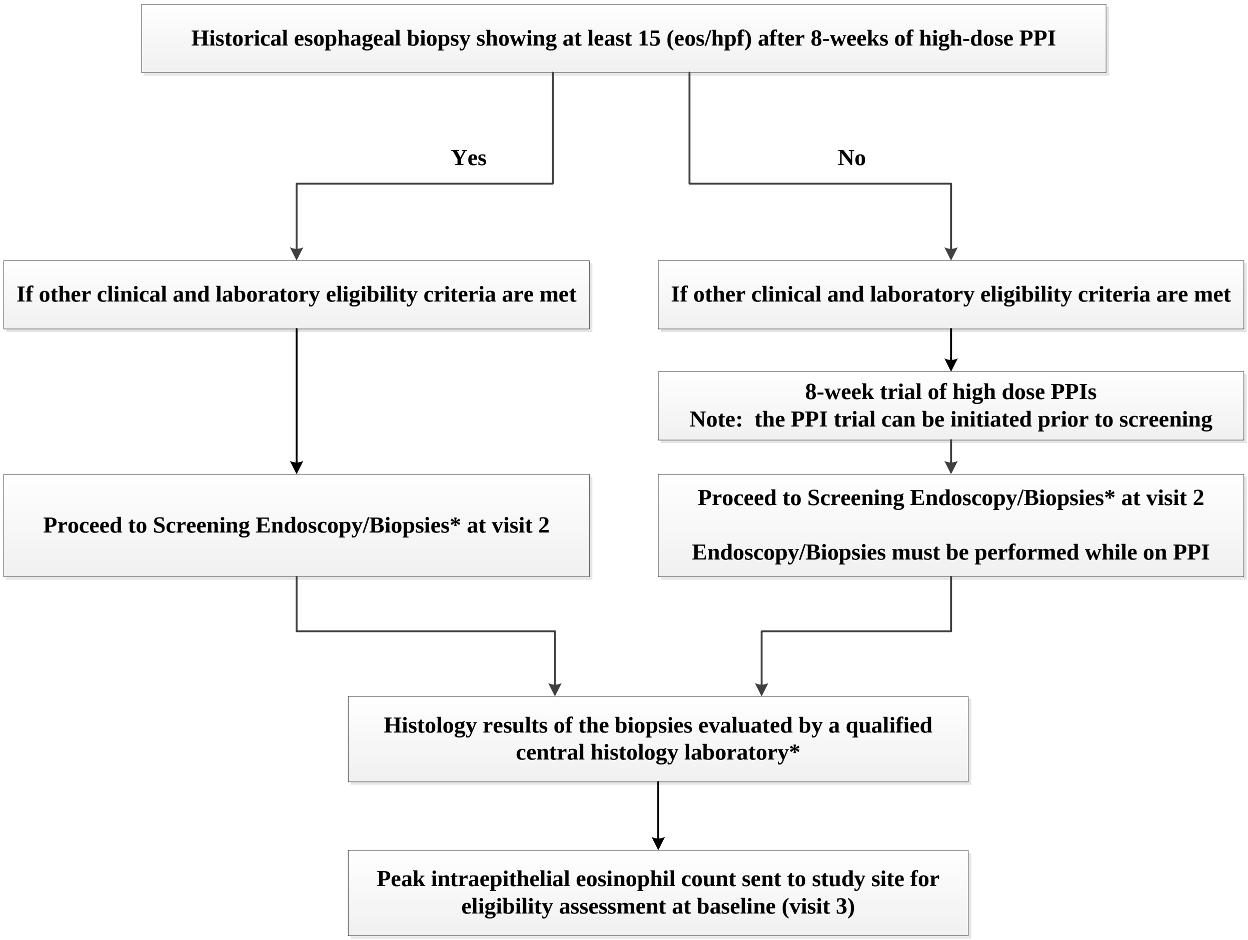

*Biopsy specimens from the antrum and/or duodenum will be obtained in all patients <18 years of age to rule out alternate etiologies. Antral and/or duodenal biopsies will be obtained in adults only in the event of abnormal endoscopic findings (other than typical EoE findings) or clinical suspicion of alternate etiologies. All randomized patients found to have stomach and/or duodenal abnormalities at baseline will have follow-up stomach and/or duodenal biopsies at week 24 and week 52.

5.1.1.2. Randomization

At the baseline visit (visit 3), patients who continue to meet eligibility criteria will enter the 24-week treatment period and be randomized in a 1:1 ratio to dupilumab 300 mg SC QW or placebo SC. Patients may be randomized as soon as their baseline endoscopy/biopsy results are available and DSQ eDiary entries are completed.

Randomization will be stratified by age (≥18 vs. ≥12 to <18 years of age) and use of PPI at randomization (yes vs. no).

5.1.1.3. Placebo-Controlled Double-Blind Treatment Period (24 Weeks)

During the approximately 24-week, placebo-controlled, double-blind treatment period, clinic visits are scheduled per the schedule of events (Table 1). Patients and parents/caregivers will be trained on injecting study drug at the first 3 visits during the double-blind treatment period. Patients will be closely monitored at the study site at visits 3 to 6 (baseline visit, study weeks 1, 2, and 4) for a

VV-RIM-00127522-1.0 Approved - 05 Nov 2020 GMT-5:00

minimum of 30 minutes after the administration of study drug. During weeks when no clinic visit is scheduled or if a scheduled visit cannot occur due to COVID-19 restrictions, the patient or parent/caregiver will administer study drug. Doses of study drug administered at home should be administered one week after the prior dose of study drug. Study drug administration that occurs in clinic should occur per the Schedule of Events in Tables 1 and 2. Patients and parents/caregivers who prefer to have clinic staff administer study drug may choose to have injections administered in the clinic. Study drug may be shipped from the clinical site to the patient's home if necessary.

The planned end of treatment visit for the double-blind treatment period is at approximately week 24. The co-primary endpoints will be assessed at week 24, one week after the last dose of study drug during the double-blind treatment period.

Patients who prematurely discontinue study treatment will be encouraged to remain in the study and attend all subsequent scheduled visits.

At the end of double-blind treatment visit (week 24), eligible patients in Part A may enter Part C, which consists of an approximately 28-week extended active treatment period. Eligibility requirements for the extended active treatment period are provided in Section 6.2.3. Patients who do not enter Part C may enter a 12-week follow-up period (Section 5.1.4).

Patients who participate in Part A are not eligible to participate in Part B.

5.1.2. Part B

Enrollment for Part B will begin immediately after the last patient is enrolled in Part A.

5.1.2.1. Screening (Up to 12 Weeks)

The screening procedures for Part B are identical to those described above for Part A.

5.1.2.2. Randomization

At the baseline visit (visit 3), patients who continue to meet eligibility criteria will enter the 24-week double-blind treatment period and will be randomized in a 1:1:1 ratio to dupilumab 300 mg SC QW, dupilumab 300 mg SC Q2W, or placebo SC. Eligible patients may be randomized as soon as their baseline endoscopy/biopsy results are available and DSQ eDiary entries are completed.

Randomization will be stratified by age (≥18 vs ≥12 to <18 years of age) and use of PPI at randomization (yes vs. no).

5.1.2.3. Placebo-Controlled Double-Blind Treatment Period (24 Weeks)

Procedures for Part B are identical to those described above for Part A.

Patients who prematurely discontinue study treatment will be encouraged to remain in the study and attend all subsequent scheduled visits.

At the end of double-blind treatment visit (week 24), eligible patients in Part B may enter Part C, which consists of an approximately 28-week extended active treatment period. Eligibility requirements for the extended active treatment period are provided in Section 6.2.3. Patients who do not enter Part C will enter a 12-week follow-up period (Section 5.1.4).

VV-RIM-00127522-1.0 Approved - 05 Nov 2020 GMT-5:00

5.1.3. Part C (28-Week Extended Active Treatment Period)

At the end of the double-blind treatment visit (week 24), eligible patients in Parts A and B may enter an approximately 28-week extended active treatment period where all patients will receive active treatment with dupilumab but only patients in Part B will be blinded to treatment regimen in Part C. The eligibility criteria for Part C are provided in Section 6.2.3.

Patients from Part A who are randomized to placebo during the double-blind treatment period will receive dupilumab 300 mg QW during Part C. Patients from Part A who are randomized to dupilumab 300 mg QW during the double-blind treatment period will continue to receive dupilumab 300 mg QW during Part C.

Patients from Part B who are randomized to placebo during the double-blind treatment period will be re-randomized in a 1:1 ratio to dupilumab 300 mg QW or dupilumab 300 mg Q2W. Patients randomized to dupilumab 300 mg Q2W will also receive matching placebo alternating with dupilumab doses so the injection frequency will be identical for both groups for regimen-blinding purposes. All other patients will remain on the same dupilumab dose regimen to which they are randomized during the double-blind treatment period. Treatment assignment in Part C will be managed by an interactive voice/web response system (IVRS/IWRS) to maintain blinding of treatment assignment in Part B.

5.1.4. Follow-Up Period (12 Weeks)

All patients will be followed up for an additional 12 weeks after completing Part C. Patients in Parts A and B who choose not to participate in or are ineligible for Part C will be followed for an additional 12 weeks immediately after their respective trial part.

5.1.5. End of Study Definition

The end of study is defined as the last visit of the last patient in the study.

5.2. Planned Interim Analysis

No interim analysis (IA) is planned.

Timing of the double-blind treatment period and extended active treatment period analyses are described in Section 10.4.9.

5.3. Study Committees

5.3.1. Independent Data Monitoring Committee

An independent Data Monitoring Committee (DMC), composed of members who are independent from the sponsor and the study investigators, will monitor patient safety by conducting formal reviews of accumulated safety data that will be blinded by treatment group; if requested, the DMC may have access to the treatment allocation code or any other requested data for the purposes of a risk-benefit assessment.

The DMC will provide the sponsor with appropriate recommendations on the conduct of the clinical study to ensure the protection and safety of the patients enrolled in the study.

VV-RIM-00127522-1.0 Approved - 05 Nov 2020 GMT-5:00

All activities and responsibilities of the DMC are described in the DMC charter.

6. SELECTION, WITHDRAWAL, AND REPLACEMENT OF PATIENTS

6.1. Number of Patients Planned

Approximately 80 patients (40 per treatment group) are planned to be enrolled in Part A. Approximately 210 patients (70 per treatment group) are planned to be enrolled in Part B at multiple global sites.

6.2. Study Population

Eligible patients for this study consist of adult males and females ≥18 years of age and adolescent males and females ≥12 to <18 years of age at the time of study entry with EoE. At least 10% of patients enrolled in Part B will be adolescents ≥12 to <18 years of age. At least 30% of patients enrolled in Part B must have a history of prior use of swallowed topical corticosteroids for the treatment of EoE.

6.2.1. Inclusion Criteria for Parts A and B

A patient must meet the following criteria to be eligible for inclusion in Part A and Part B of the study:

1. Male or female, ≥12 years of age
2. A documented diagnosis of EoE by endoscopic biopsy prior to screening, as demonstrated by intraepithelial eosinophilic infiltration (peak cell count ≥15 eos/hpf) from at least one esophageal region and performed after at least 8 weeks of treatment with a high-dose PPI regimen. If the patient discontinued PPI therapy, the biopsy must have been performed within 2 weeks of the date of discontinuation.

   If a prior (historical) endoscopic biopsy meeting these criteria is not available (or no prior biopsy is available), patients who meet other clinical and laboratory eligibility criteria will undergo treatment with a high-dose PPI regimen for at least 8 weeks before their baseline endoscopy/biopsies. (See Section 7.2 for acceptable high-dose PPI regimens).

   Note: If the patient is already using an acceptable high dose PPI regimen at the time of the screening visit, the baseline endoscopy may be scheduled at any point during the screening period after 8 weeks of treatment have been documented.
3. Baseline endoscopic biopsies with a demonstration on central reading of intraepithelial eosinophilic infiltration (peak cell count ≥15 eos/hpf) in at least 2 of the 3 biopsied esophageal regions (proximal, mid, or distal)
4. History (by patient report) of an average of at least 2 episodes of dysphagia (with intake of solids) per week in the 4 weeks prior to screening

VV-RIM-00127522-1.0 Approved - 05 Nov 2020 GMT-5:00

5. At least 4 episodes of dysphagia in the 2 weeks prior to baseline, documented via eDiary, at least 2 of which require liquids, coughing or gagging, vomiting, or medical attention to obtain relief
6. Completed at least 11 of 14 days of DSQ eDiary data entry in the 2 weeks prior to the baseline visit (visit 3)
7. Baseline DSQ score ≥10.
8. Able to understand and complete study-related questionnaires
9. Willing and able to comply with clinic visits and study-related procedures
10. Provide informed consent signed by study patient or legally acceptable representative. For adolescents, parent or legal guardian must provide signed informed consent (patients must also provide separate informed assent to enroll in the study, and the assent documented either in a separate informed assent form [IAF] or in the informed consent form [ICF] signed by the parent(s)/legal guardian [as appropriate based on local regulations and requirements]).

6.2.2. Exclusion Criteria for Parts A and B

A patient who meets any of the following criteria will be excluded from Part A and Part B of the study:

1. Body weight ≤40 kg
2. Prior participation in a dupilumab clinical trial, or past or current treatment with dupilumab
3. Initiation or change of a food-elimination diet regimen or re-introduction of a previously eliminated food group in the 6 weeks prior to screening. Patients on a food-elimination diet must remain on the same diet throughout the study.
4. Other causes of esophageal eosinophilia or the following conditions: hypereosinophilic syndrome and eosinophilic granulomatosis with polyangiitis (Churg-Strauss syndrome)

   Note: Patients with eosinophilic gastroenteritis are eligible, provided they meet other eligibility criteria.
5. Active Helicobacter pylori infection
6. History of achalasia, Crohn’s disease, ulcerative colitis, celiac disease, and prior esophageal surgery
7. Any esophageal stricture unable to be passed with a standard, diagnostic, 9 to10 mm upper endoscope or any critical esophageal stricture that requires dilation at screening
8. History of bleeding disorders or esophageal varices that, in the opinion of the investigator, would put the patient at undue risk for significant complications from an endoscopy procedure
9. Treatment with swallowed topical corticosteroids within 8 weeks prior to baseline

VV-RIM-00127522-1.0 Approved - 05 Nov 2020 GMT-5:00

10. Initiation, discontinuation, or change in the dosage regimen of the following medications within 8 weeks prior to the baseline endoscopy:
    - Proton pump inhibitors (except for patients who require a PPI trial prior to baseline endoscopy)
    - Leukotriene inhibitors
    - Nasal and/or inhaled corticosteroids
    - Patients on a stable dose of these medications for at least 8 weeks prior to the baseline endoscopy may be included in the study, but must not change the dose during the study.
11. Initiation, discontinuation, or change in the dosage regimen of SC immunotherapy (SCIT)
    - Patients on a stable dose of these medications for at least 1 year prior to visit 1 may be included in the study, but must not change the dose during the study.
12. Treatment with sublingual immunotherapy (SLIT)
13. Treatment with oral immunotherapy (OIT) within 6 months prior to visit 1
14. The following treatments within 3 months prior to screening, or any condition that, in the opinion of the investigator, is likely to require such treatment(s) during the study:
    - Systemic immunosuppressant/immunomodulating drugs, including but not limited to systemic corticosteroids, omalizumab, cyclosporine, mycophenolate-mofetil, interferon-gamma [IFN-γ], Janus kinase inhibitors, azathioprine, and methotrexate
      - Note: One-time use of a corticosteroid as a part of the anesthetic preparation used during each endoscopy procedure is allowed.
15. Treatment with an investigational drug within 2 months or within 5 half-lives (if known), whichever is longer, prior to visit 1
16. Planned or anticipated use of any prohibited medications and procedures during the study
17. Planned or anticipated major surgical procedure during the study
18. Treatment with a live (attenuated) vaccine within 4 weeks prior to the baseline visit
19. Active parasitic infection or suspected parasitic infection, unless clinical and (if necessary) laboratory assessments have ruled out active infection before randomization
20. Chronic or acute infection requiring treatment with systemic antibiotics, antivirals, or antifungals within 2 weeks before baseline visit.

    Note: A patient may be re-screened after the infection resolves.

21. Known or suspected immunodeficiency disorder, including history of invasive opportunistic infections (eg, tuberculosis [TB], non-tuberculous mycobacterial infections, histoplasmosis, listeriosis, coccidioidomycosis, pneumocystosis, aspergillosis) despite infection resolution, or otherwise recurrent infections of abnormal frequency, or prolonged infections suggesting an immune-compromised status, as judged by the investigator.

VV-RIM-00127522-1.0 Approved - 05 Nov 2020 GMT-5:00

Tuberculosis testing will be performed on a country-by-country basis according to local guidelines if required by regulatory authorities or ethics committees (ECs).

22. Known history of human immunodeficiency virus (HIV) infection
23. Established diagnosis of hepatitis B viral infection at the time of screening or positive for hepatitis B surface antigen (HBsAg) at the time of screening
    - Patients who have gained immunity for hepatitis B virus infection after vaccination (patients who are HBsAg negative, hepatitis B surface antibody [HBsAb] positive, and hepatitis B core antibody [HBcAb] negative are eligible for the study).
    - Patients with positive HBcAb are eligible for the study only if hepatitis B virus DNA level is undetectable.
24. Established diagnosis of hepatitis C viral (HCV) infection at the time of screening Patients positive for hepatitis C Ab are eligible for the study only if HCV RNA is negative.
25. On current treatment for hepatic disease including but not limited to acute or chronic hepatitis, cirrhosis, or hepatic failure, or has evidence of liver disease as indicated by persistent (confirmed by repeated tests ≥2 weeks apart) elevated transaminases (alanine aminotransferase [ALT] and/or aspartate aminotransferase [AST]) more than 3 times the upper limit of normal [ULN] during the screening period)
26. Any of the following abnormal lab values at screening:
    - Platelets <100 $\times 10^3/\mu L$
    - Neutrophils <1.5 $\times 10^3/\mu L$
    - Estimated glomerular filtration rate (eGFR) <30 mL/min/1.73 m^2

    Note: If an abnormal value is detected at screening, a repeat test should be performed to confirm the abnormality. Only if the repeat test confirms the abnormality would the patient be categorized as a screen failure. eGFR will be calculated using the Modification of Diet in Renal Disease (MDRD) equation in adult patients and using the Bedside Schwartz formula in patients <18 years of age.
27. Severe concomitant illness(es) that, in the investigator's judgment, would adversely affect the patient's participation in the study. Examples include but are not limited to short life expectancy, uncontrolled diabetes, cardiovascular conditions (eg, NYHA Class III or IV cardiac failure), severe renal conditions (eg, severe nephrotic syndrome), hepatobiliary conditions (eg, Child-Pugh class B or C), neurological conditions (eg, demyelinating diseases), active major autoimmune diseases (eg, lupus, inflammatory bowel disease, rheumatoid arthritis, etc.), other severe endocrinologic, gastrointestinal, metabolic, pulmonary, or lymphatic diseases. The specific justification for patients excluded under this criterion will be noted in study documents (chart notes, case report forms [CRF], etc).
28. History of malignancy within 5 years prior to screening, except completely treated in situ carcinoma of the cervix and completely treated non-metastatic squamous or basal cell carcinoma of the skin
29. History of alcohol or drug abuse within 6 months prior to screening

VV-RIM-00127522-1.0 Approved - 05 Nov 2020 GMT-5:00

30. Any other medical or psychological condition including relevant laboratory abnormalities at screening that, in the opinion of the investigator, suggest a new and/or insufficiently understood disease, may present an unreasonable risk to the study patient as a result of his/her participation in this clinical trial, may make patient's participation unreliable, or may interfere with study assessments. The specific justification for patients excluded under this criterion will be noted in study documents.
31. Patient or his/her immediate family is a member of the investigational team
32. Pregnant or breastfeeding women, or women planning to become pregnant or breastfeed during the study
33. Women of childbearing potential* who are unwilling to practice highly effective contraception prior to the initial dose/start of the first treatment, during the study, and for at least 12 weeks after the last dose. Highly effective contraceptive measures include:
    a. Stable use of combined (estrogen and progestogen containing) hormonal contraception (oral, intravaginal, transdermal) or progestogen-only hormonal contraception (oral, injectable, implantable) associated with inhibition of ovulation initiated 2 or more menstrual cycles prior to screening
    b. Intrauterine device; intrauterine hormone-releasing system
    c. Bilateral tubal ligation
    d. Vasectomized partner
    e. And/or sexual abstinence†, ‡.

    *Postmenopausal women must be amenorrheic for at least 12 months in order not to be considered of childbearing potential. Pregnancy testing and contraception are not required for women with documented hysterectomy or tubal ligation.

    †Sexual abstinence is considered a highly effective method only if defined as refraining from heterosexual intercourse during the entire period of risk associated with the study treatments. The reliability of sexual abstinence needs to be evaluated in relation to the duration of the clinical trial and the preferred and usual lifestyle of the patient.

    ‡Periodic abstinence (calendar, symptothermal, post-ovulation methods), withdrawal (coitus interruptus), spermicides only, and lactational amenorrhoea method are not acceptable methods of contraception. Female condom and male condom should not be used together.
34. Known systemic hypersensitivity to dupilumab or the excipients of the drug product

VV-RIM-00127522-1.0 Approved - 05 Nov 2020 GMT-5:00

6.2.3. Exclusion Criteria for Part C (the Extended Active Treatment Period)

A patient who meets any of the following criteria will not be permitted to enter Part C (the extended active treatment period):

1. Patients who, during the double-blind treatment period, developed a serious adverse event (SAE) and/or adverse event (AE) deemed related to study drug, which in the opinion of the investigator could indicate that continued treatment with study drug may present an unreasonable risk for the patient
2. Patients who, during the double-blind treatment period, were prematurely withdrawn because of a protocol violation, poor compliance, or inability to complete required study assessments
3. Patients who became pregnant during the double-blind treatment period
4. Patients who are prematurely discontinued from study drug due to an AE (patients who are prematurely discontinued from study drug due to lack of efficacy are eligible to enter Part C)
5. Patients who did not undergo endoscopy with biopsies prior to receiving rescue treatment

   Note: If the endoscopy with biopsies cannot occur due to COVID-19 restrictions, rescue treatment should be initiated without delay and these patients will be eligible to participate in Part C when the patient can next return to the clinic.
6. Systemic hypersensitivity to dupilumab or the excipients of the drug product based on participation in Part A or Part B

6.3. Premature Withdrawal from the Study

A patient has the right to withdraw from the study at any time, for any reason, and without repercussion.

The investigator and/or sponsor have the right to withdraw a patient from the study if it is no longer in the interest of the patient to continue in the study, or if the patient’s continuation in the study places the scientific outcome of the study at risk (eg, if a patient does not or cannot follow study procedures). An excessive rate of withdrawals would render the study uninterpretable; therefore, unnecessary withdrawal of patients should be avoided.

Patient who are withdrawn prematurely from the study will be asked to complete study assessments, as described in Section 8.1.2.

Rules for discontinuation of study treatment (permanent or temporary) are discussed in Section 7.4.2.

6.4. Replacement of Patient

Patient prematurely discontinued from study/study drug will not be replaced.

VV-RIM-00127522-1.0 Approved - 05 Nov 2020 GMT-5:00

7. STUDY TREATMENTS

7.1. Investigational and Reference Treatments

Dupilumab drug product is supplied for this study in the following concentration:

- Dupilumab 150 mg/mL: Each 2.0 mL single-use prefilled glass syringe with snap-off cap delivers 300 mg of study drug (2.0 mL of a 150 mg/mL solution)

Placebo matching dupilumab is prepared in the same formulation without the addition of protein (ie, active substance, anti-IL-4Rα monoclonal Ab).

In the double-blind placebo-controlled Parts A and B, as well as Part C, all patients will receive QW SC injections. For the dupilumab 300 mg SC Q2W group, in order to maintain the blind, there will be an SC injection of placebo in between dupilumab doses so the injection frequency will match the other 2 groups (dupilumab QW and placebo) (see randomization/treatment assignment in Section 5.1.1.2 for Part A, Section 5.1.2.2 for Part B, Section 5.1.3 for Part C, and Section 7.5). In the extended active treatment Part C, patients will receive dupilumab injections at the frequency (QW or Q2W with matching placebo alternating with dupilumab doses so the injection frequency will be identical for both groups for regimen-blinding purposes) per their treatment assignment (see Section 5.1.3 and Section 7.5).

Study drug will be administered by SC injections. Subcutaneous injection sites of study drug should be alternated among the different quadrants of the abdomen (avoiding navel and waist areas), upper thighs, and upper arms so that the same site is not injected for 2 consecutive administrations.

Instructions on dose preparation are provided in the pharmacy manual.

7.2. Background Treatment

Patients who undergo a trial of high-dose PPI therapy initiated prior to screening or during the screening period of Part A or Part B must remain on a dosage regimen listed below for the duration of the 52-week treatment period. A high-dose PPI regimen is defined as follows:

- omeprazole 40 mg once a day (QD) or 20 mg twice a day (BID)
- esomeprazole 40 mg QD or 20 mg BID
- lansoprazole 60 mg QD or 30 mg BID
- dexlansoprazole 60 mg QD
- rabeprazole 40 mg QD or 20 mg BID
- pantoprazole 80 mg QD or 40 mg BID

Patients who present at the initial screening visit with current use of PPIs must also remain on the same or similar approved dosage regimen for the duration of the 52-week treatment period. Patients may change to a different approved PPI medication during the study. PPI therapy is prohibited for all other patients.

VV-RIM-00127522-1.0 Approved - 05 Nov 2020 GMT-5:00

7.3. Rescue Treatments

If medically necessary (eg, for treatment of intolerable EoE symptoms), rescue medications (systemic and/or swallowed topical corticosteroids) or emergency esophageal dilation are allowed for study patients in Parts A, B, and C. An endoscopy with biopsy will be performed prior to the initiation of rescue therapy. Patients who undergo an endoscopy with biopsy due to the initiation of rescue therapy will not undergo the scheduled end of treatment visit endoscopy/biopsy at weeks 24 and 52. Patients who receive rescue treatment during the double-blind period of the study will not be eligible for the extended active treatment period unless an endoscopy with biopsy is performed prior to the initiation of rescue treatment. However, if the endoscopy with biopsies cannot occur due to COVID-19 restrictions, rescue treatment should not be delayed, and these patients will be eligible for Part C. Part C treatment will be initiated per the schedule of events and only at an in-clinic visit.

Patients receiving rescue therapy may continue to receive study drug. They will remain blinded and will be asked to return to the clinic for all remaining study visits for the double-blind treatment period and the follow-up period, and participate in all assessments for these visits according to the schedule of events specified in Table 1, Table 2, and Table 3, except for endoscopy/biopsy, as noted above. For the purpose of efficacy analyses, patients who receive rescue treatment during the study will be considered treatment failures.

7.4. Dose Modification and Study Treatment Discontinuation Rules

7.4.1. Dose Modification

Dose modification for an individual patient is not allowed.

7.4.2. Study Drug Discontinuation

Patients who permanently discontinue from study drug during the double-blind treatment period will be asked to return to the clinic for all remaining study visits for the double-blind treatment period and follow-up period per the visit schedule.

Patients who permanently discontinue from study drug during the extended active treatment period will be asked to return to the clinic for all remaining study visits per the visit schedule.

Patients who permanently discontinue from study drug and who opt to withdraw from the study will be asked to complete an early termination (ET) visit, per Section 8.1.2.

7.4.2.1. Reasons for Permanent Discontinuation of Study Drug

Study drug dosing will be permanently stopped in the event of:

- Evidence of pregnancy
- Anaphylactic reaction or other severe systemic reaction deemed related to study drug
- Diagnosis of a malignancy during study, excluding carcinoma in situ of the cervix or squamous or basal cell carcinoma of the skin. Patient must be withdrawn for these latter malignancies if they cannot be adequately treated by local resection.

VV-RIM-00127522-1.0 Approved - 05 Nov 2020 GMT-5:00

- Any infection that is opportunistic, such as TB and other infections whose nature or course may suggest an immunocompromised status
- Severe laboratory abnormalities assessed as related to study drug:
  – Neutrophil count ≤0.5 × 10^3/µL
  – Platelet count ≤50 × 10^3/µL
  – ALT and/or AST values >3 × ULN with total bilirubin >2 × ULN, excluding confirmed Gilbert's Syndrome
  – Confirmed AST and/or ALT >5 × ULN (for more than 2 weeks)

7.4.2.2. Reasons for Temporary Discontinuation of Study Drug

Study drug dosing may be temporarily discontinued in the event of:

- Severe laboratory abnormalities (as noted in Section 7.4.2.1) where a causal relationship to study drug can be reasonably excluded, (ie, an alternative cause is evident): study drug will be discontinued but may be resumed when the laboratory abnormality is sufficiently normalized. At minimum, the laboratory value(s) must return to a level that no longer meets the specified criteria for discontinuation, as defined in Section 7.4.2.1. A decision to resume study treatment will be made jointly by the investigator and medical monitor (medical monitor's written approval is required).
- An infection that requires parenteral treatment with antibiotic, antifungal, antiviral, antiparasitic, or antiprotozoal agents, or requires oral treatment with such agents for longer than 2 weeks
- Other intercurrent illnesses or major surgery which could, in the opinion of the investigator, present an unreasonable risk to the patient as a result of his/her continued participation in the study

After the condition leading to suspension of dosing normalizes sufficiently, study treatment may resume at the discretion of the principal investigator in consultation with the medical monitor.

A decision to temporarily discontinue study drug and/or to reinstitute study treatment should be discussed with the medical monitor. The investigator may suspend study treatment at any time, even without consultation with the medical monitor if the urgency of the situation requires immediate action and if this is determined to be in the patient's best interest. However, the medical monitor should be contacted as soon as possible in any case of study drug discontinuation. Resumption of study treatment after temporary discontinuation requires consultation and agreement between the investigator and the medical monitor.

VV-RIM-00127522-1.0 Approved - 05 Nov 2020 GMT-5:00

7.5. Method of Treatment Assignment

In Part A, approximately 80 patients (40 per group) will be randomized in a 1:1 ratio to receive dupilumab 300 mg QW or placebo according to a central randomization scheme provided by an IVRS/IWRS to the designated study pharmacist (or qualified designee).

In Part B, approximately 210 patients will be randomized in a 1:1:1 ratio to receive dupilumab 300 mg QW, dupilumab 300 mg Q2W, or placebo (approximately 70 patients per group) according to a central randomization scheme provided by IVRS/IWRS to the designated study pharmacist (or qualified designee).

Randomization for both Part A and Part B will be stratified according to age at the time of the screening visit (≥12 to <18 years of age vs. ≥18 years of age) and use of PPI at randomization (yes vs. no).

For patients entering Part C from Part B, those who are randomized to placebo during Part B will be re-randomized in a 1:1 ratio to receive dupilumab 300 mg QW or dupilumab 300 mg Q2W (with matching placebo alternating with dupilumab doses so the injection frequency will be identical for both groups for regimen-blinding purposes) in Part C, and those who are randomized to one of the dupilumab dose regimens in Part B will remain on the same dupilumab dose regimen in Part C. Patients who received 300 mg Q2W in Part B will also continue with matching placebo alternating with dupilumab doses so the injection frequency will be identical for both groups for regimen-blinding purposes. Treatment assignment in Part C for all patients from Part B, regardless of their previously assigned treatment in Part B, will be managed by IVRS/IWRS to maintain blinding.

7.5.1. Blinding

Study patients, the principal investigators, and study site personnel will remain blinded to all randomization assignments throughout the study. The blinded Regeneron Medical/Study Director, Study Monitor, and any other Regeneron and contract research organization (CRO) personnel who are in regular contact with the study site will remain blinded to all patient randomization assignments.

All study personnel will be blinded to data during each study part until the respective database lock. Part A will be unblinded while Part B is ongoing.

Blinded study drug kits coded with a medication numbering system will be used. In order to maintain the blind, lists linking these codes with product lot numbers will not be accessible to individuals involved in study conduct.

Anti-drug antibody, drug concentration results, and any post-treatment tissue eosinophil counts and histologic results, and biomarker results (eotaxin-3, thymus and activation-regulated chemokine [TARC], total IgE and allergen-specific IgEs) will not be communicated to the sites, and the sponsor’s blinded operational team will not have access to results associated with patient identification until after the database is locked for the respective study part.

VV-RIM-00127522-1.0 Approved - 05 Nov 2020 GMT-5:00

7.5.2. Emergency Unblinding

Unblinding of treatment assignment for a patient may be necessary due to a medical emergency or any other significant medical event (eg, pregnancy).

- If unblinding is required:
  - Only the investigator will make the decision to unblind the treatment assignment.
  - Only the affected patients will be unblinded.
  - The designated study pharmacist(s)/designee at the study site will provide the treatment assignment to the investigator. If there is no study pharmacist, the investigator for the site will unblind the patient.
  - The investigator will notify Regeneron and/or designee as soon as possible after unblinding the patient.

Treatment assignment is not to be provided to site personnel, other than the unblinded study pharmacist (when applicable), at any time during the conduct of the study, except in the case of a true emergency. In the event that there is no study pharmacist, the individual at the site fulfilling that role will be the only unblinded member of the site personnel.

7.5.3. Unblinding for Regulatory Reporting Purposes

Treatment assignments for certain patients may be unblinded to Pharmacovigilance personnel for the purpose of regulatory reporting of suspected unexpected serious adverse reactions (SUSARs) to health authorities in accordance with local regulations.

7.6. Treatment Logistics and Accountability

7.6.1. Packaging, Labeling, and Storage

A medication numbering system will be used to label blinded investigational study drug. Lists linking medication numbers with product lot numbers will be maintained by the groups (or companies) responsible for study drug packaging. In order to maintain the blind, these lists will not be accessible to individuals involved in study conduct.

Study drug will be stored at the site at a temperature of 2ºC to 8ºC; storage instructions will be provided in the pharmacy manual.

7.6.2. Supply and Disposition of Treatments

Study drug will be shipped at a temperature of 2ºC to 8ºC to the investigator or designee at regular intervals or as needed during the study. At specified time points during the study (eg, interim site monitoring visits), at the site close-out visit, and following drug reconciliation and documentation by the site monitor, all opened and unopened study drug will be returned to the sponsor or designee for destruction, unless it’s specified that the site may perform their own destruction.

7.6.3. Treatment Accountability

All drug accountability records must be kept current.

The investigator must be able to account for all opened and unopened study drug. These records should contain the dates, quantity, and study drug that is:

- dispensed to each patient,
- returned from each patient (if applicable), or
- disposed of at the site or returned to the sponsor or designee.

All accountability records must be made available for inspection by the sponsor and regulatory agency inspectors; photocopies must be provided to the sponsor at the conclusion of the study.

7.6.4. Treatment Compliance

All drug compliance records must be kept current and made available for inspection by the sponsor and regulatory agency inspectors.

7.7. Concomitant Medications and Procedures

Any treatment administered from the time of informed consent to the final study visit will be recorded. Any treatment administered from the time of first dose of study drug to the final study visit will be considered concomitant medication; this includes medications that were started before the study and are ongoing during the study.

7.7.1. Prohibited Medications and Procedures

Treatment with the following concomitant medications is prohibited through week 52:

- Swallowed topical corticosteroids (may be used as rescue treatment for EoE)
- Systemic corticosteroids (may be used as rescue treatment for EoE)
    - Note: One-time use of a corticosteroid as a part of the anesthetic preparation used during each endoscopy procedure is allowed.
- Systemic immunosuppressive/immunomodulating drugs (including, but not limited to, omalizumab, cyclosporine, mycophenolate-mofetil, azathioprine, methotrexate, IFN-γ, or other biologics)
- Treatment with an investigational drug (other than dupilumab)
- Initiation, discontinuation, or change in dosage regimen of the following medications within 8 weeks prior to the baseline endoscopy (stable doses of these medications are allowed)
    - Proton pump inhibitors, unless used for a required PPI trial during the screening period or in patients who present at the initial screening visit with current use of PPIs
    - Systemic leukotriene inhibitors

VV-RIM-00127522-1.0 Approved - 05 Nov 2020 GMT-5:00

  - Nasal and/or inhaled corticosteroids
- Initiation of SCIT, or change in dose for those patients on a stable dose of SCIT within 1 year prior to screening
- SLIT
- OIT
- Treatment with an investigational drug (other than dupilumab)
- Treatment with a live (attenuated) vaccine

Chickenpox (varicella)	Oral typhoid
FluMist-Influenza	Rubella
Intranasal influenza	Smallpox (vaccinia)
Measles (rubeola)	Yellow fever
Measles-mumps-rubella combination	Bacille Calmette-Guerin
Measles-mumps-rubella-varicella combination	Rotavirus
Mumps	Varicella zoster (shingles)
Oral polio (Sabin)	

The following concomitant procedures are prohibited during study treatment (through week 52):

- Major elective surgical procedures
- Esophageal dilation (may be used as rescue procedure)
- Initiation or change of food-elimination diet regimen

7.7.2. Permitted Medications and Procedures

Other than the prohibited medications listed in Section 7.7.1, treatment with concomitant medications are permitted during the study. Medications used to treat chronic disease such as diabetes and hypertension are also permitted. If there is any question regarding whether a concomitant medication may be used during the study, the study site should contact the medical monitor.

7.7.3. Restricted Medications and Procedures during the Follow-Up Period

Patients may receive the prohibited medications/procedures listed in Section 7.7.1 as needed during the follow-up period, with the exception of live (attenuated) vaccine, which should not be used within 3 months after the last dose of study drug. Investigators are advised to prescribe prohibited medications/procedures judiciously, only when they are absolutely required for the appropriate management of study patients.

8. STUDY SCHEDULE OF EVENTS AND PROCEDURES

8.1. Schedule of Events

Study assessments and procedures are presented by study period and visit in Table 1 for the screening period and double-blind treatment period in Parts A and B, in Table 2 for the extended active treatment period in Part C, and in Table 3 for the follow-up period, ET visit, and unscheduled visit.

In light of the public health emergency related to COVID-19, the continuity of clinical study conduct and oversight may require implementation of temporary or alternative mechanisms. Examples of such mechanisms may include, but are not limited to, any of the following: phone contact, virtual visits, telemedicine visits, online meetings, non-invasive remote monitoring devices, use of local clinic or laboratory locations, and home visits by skilled staff. Additionally, no waivers to deviate from protocol enrollment criteria due to COVID-19 will be granted. All temporary mechanisms utilized, and deviations from planned study procedures are to be documented as being related to COVID-19 and will remain in effect only for the duration of the public health emergency.

VV-RIM-00127522-1.0 Approved - 05 Nov 2020 GMT-5:00

Table 1: Parts A and B Schedule of Events – Screening Period and Double-Blind Treatment Period

Study Procedure	Screening Period		24-Week Double-Blind Treatment Period[18]								
	Screening[1] V1	Screening Endoscopy/ Biopsy[2] V2	Baseline V3	V4	V5	V6	V7	V8	V9	V10	DB EOT V11[3]
**Week (W)**				**W1**	**W2**	**W4**	**W8**	**W12**	**W16**	**W20**	**W24[3]**
**Day (D)**	**D-85 to D-29[1]**	**D-21[2]**	**D1**	**D8**	**D15**	**D29**	**D57**	**D85**	**D113**	**D141**	**D169**
**Visit Window (Days [d])**		**±7 d**		**±3 d**	**±3 d**	**±3 d**	**±3 d**	**±3 d**	**±3 d**	**±3 d**	**+7 d**
**Screening[4]/Baseline:**											
Informed consent and assent	X										
Inclusion/Exclusion criteria	X	X	X								
Med. history, Demographics	X										
Randomization[2a]			X[2a]								
**Treatment:**											
Training for self-injection[5]			X	X[6a]	X[6a]						
Administer study drug[6, 6a]			X	X	X	X	X	X	X	X	
Study drug dispensation[6, 6a]				X	X	X	X	X	X	X	
Study drug accountability				X[6a]	X[6a]	X	X	X	X	X	X
Concomitant medications/procedures	X	X	X	X	X	X	X	X	X	X	X
**Efficacy:[7]**											
DSQ PRO (daily)[8]	assessed by patient daily using eDiary										
PGIC[9]								X		X	X
PGIS[9]			X					X		X	X
EoE Impact Questionnaire [10]			X					X			X
EoE Symptom Questionnaire[10]			X					X			X
TNSS[11]			X					X			X
RQLQ(S)+12[11]			X					X			X
ACQ-5[11]			X					X			X
POEM[11]			X					X			X
EQ-5D-3L	X[17]		X[17]								
EoE-EREFS[2,12]		X[2, 2a, 4]									X[2, 2b]
Endoscopy with biopsies (histology, IHC, RNA, EndoFLIP)[2]		X[2, 2a, 4]									X[2, 2b]

Study Procedure	Screening Period		24-Week Double-Blind Treatment Period[18]								
	**Screening[1]** **V1**	**Screening Endoscopy/ Biopsy[2]** **V2**	**Baseline** **V3**	**V4**	**V5**	**V6**	**V7**	**V8**	**V9**	**V10**	**DB EOT** **V11[3]**
**Week (W)**				**W1**	**W2**	**W4**	**W8**	**W12**	**W16**	**W20**	**W24[3]**
**Day (D)**	**D-85 to D-29[1]**	**D-21[2]**	**D1**	**D8**	**D15**	**D29**	**D57**	**D85**	**D113**	**D141**	**D169**
**Visit Window (Days [d])**		**±7 d**		**±3 d**	**±3 d**	**±3 d**	**±3 d**	**±3 d**	**±3 d**	**±3 d**	**+7 d**
**Safety: [7, 7a, 18]**											
Vital signs[13]	X	X	X[13]	X[13, 13a]	X[13, 13a]	X[13, 13a]	X[13a]	X[13a]	X[13a]	X[13a]	X
Physical examination	X										X
ECG	X										X
Height	X										X[14]
Weight	X	X	X		X	X	X	X	X	X	X
Adverse events	X	X	X	x	X	X	X	X	X	X	X
**Laboratory Testing:[7, 7a]**											
Hematology, Chemistry	X		X					X			X
Serology tests [15]	X										
Serum FSH	X										
Pregnancy test[16]	Serum	Urine	Urine			Urine	Urine	Urine	Urine	Urine	Urine
Urinalysis	X		X					X			X
**PK and ADA:[7, 7a]**											
PK sample			X					X			X
ADA sample			X					X			X
**Biomarkers and Genomics:[7, 7a]**											
Whole blood DNA (optional)			X								
Whole blood RNA (optional)	X		X								X
Plasma Eotaxin-3	X		X			X		X			X
Serum TARC	X		X			X					X
Serum Total IgE	X		X			X		X			X
Allergen-specific IgE, IgG4	X		X			X		X			X
Future Biomarker Serum/Plasma	X		X			X		X			X

ADA = anti-drug antibody; DB EOT = end of double-blind treatment period; DSQ = Dysphagia Symptom Questionnaire; ECG = electrocardiogram; EoE-EREFS = Eosinophilic Esophagitis-Endoscopic Reference Score; EQ-5D-3L = European Quality of Life 5-dimension (version 3L; 3-level); IHC = immunohistochemistry; PGIC = Patient Global Impression of Change of Dysphagia; PGIS = Patient Global Impression of Severity of Dysphagia; POEM =

CONFIDENTIAL

VV-RIM-00127522-1.0 Approved - 05 Nov 2020 GMT-5:00

Patient-Oriented Eczema Measure; PRO = patient-reported outcome; PK = pharmacokinetic; RQLQ(S)+12 = Rhinoconjunctivitis Quality of Life Questionnaire for patients aged 12+ years; TNSS = Total Nasal Symptom Score; V = visit

Table 2: Part C Schedule of Events – Extended Active Treatment Period

	28-Week Extended Active Treatment Period [14]								
**Study Procedure**	**V11[1]**	**V12**	**V13**	**V14**	**V15**	**V16**	**V17**	**V18**	**EOT V19**
**Week (W)**	**W24[1]**	**W26**	**W28**	**W32**	**W36**	**W40**	**W44**	**W48**	**W52**
**Day (D)**	**D169**	**D183**	**D197**	**D225**	**D253**	**D281**	**D309**	**D337**	**D365**
**Visit Window (Days [d])**	**+7 d**	**±7 d**	**±7 d**	**±7 d**	**±7 d**	**±7 d**	**±7 d**	**±7 d**	**+7 d**
**Treatment:[2]**									
Administer study drug [3, 4, 4a]	X	X	X	X	X	X	X	X	
Study drug dispensation [4, 4a]	X	X	X	X	X	X	X	X	
Study drug accountability		X	X	X	X	X	X	X	X
Concomitant medications/procedures		X	X	X	X	X	X	X	X
**Efficacy:[2]**									
DSQ eDiary[5]	←--------------------------- completed daily by patient --------------------------------→								
PGIC[6]					X				X
PGIS[6]					X				X
EoE Impact Questionnaire[7]					X				X
EoE Symptom Questionnaire [7]					X				X
TNSS [8]					X				X
RQLQ(S)+12 [8]					X				X
POEM [8]					X				X
ACQ-5 [8]					X				X
EoE-EREFS [9, 10]									X[10, 10a]
Endoscopy with biopsies (histology, IHC, RNA, EndoFLIP)[10]									X[10, 10a]
**Safety: [2, 2a, 14]**									
Vital signs [11]	X[11]	X[11]	X	X	X	X	X	X	X
Height [12]									X
Weight		X	X	X	X				X
Physical examination									X
ECG									X
Adverse events		X	X	X	X	X	X	X	X

	28-Week Extended Active Treatment Period [14]								
**Study Procedure**	**V11[1]**	**V12**	**V13**	**V14**	**V15**	**V16**	**V17**	**V18**	**EOT V19**
**Week (W)**	**W24[1]**	**W26**	**W28**	**W32**	**W36**	**W40**	**W44**	**W48**	**W52**
**Day (D)**	**D169**	**D183**	**D197**	**D225**	**D253**	**D281**	**D309**	**D337**	**D365**
**Visit Window (Days [d])**	**+7 d**	**±7 d**	**±7 d**	**±7 d**	**±7 d**	**±7 d**	**±7 d**	**±7 d**	**+7 d**
**Laboratory Testing:[2, 2a]**									
Hematology, Chemistry					X				X
Pregnancy test [13]		Urine		Urine	Urine	Urine	Urine	Urine	Urine
Urinalysis					X				X
**PK and ADA:[2, 2a]**									
PK Sample				X					X
ADA sample				X					X
**Biomarkers:[2, 2a]**									
Whole blood RNA (optional)									X
Plasma Eotaxin-3		X		X					X
Serum TARC									X
Serum Total IgE		X		X					X
Allergen-specific IgE, IgG4		X		X					X
Future Biomarker Serum/Plasma		X		X					X

ADA = anti-drug antibody; DSQ = Dysphagia Symptom Questionnaire; ECG = electrocardiogram; EoE-EREFS = Eosinophilic Esophagitis-Endoscopic Reference Score; EOT = end of extended active treatment period; IHC = immunohistochemistry; POEM = Patient-Oriented Eczema Measure; PRO = patient reported outcome; PGA = physician global assessment; PGIC = Patient Global Impression of Change of Dysphagia; PGIS = Patient Global Impression of Severity of Dysphagia; PK = pharmacokinetic; RQLQ(S)+12 = Rhinoconjunctivitis Quality of Life Questionnaire for patients aged 12+ years; TNSS = Total Nasal Symptom Score; V = visit

CONFIDENTIAL

VV-RIM-00127522-1.0 Approved - 05 Nov 2020 GMT-5:00

Table 3: Follow-up Period, Early Termination Visit, and Unscheduled Visit

Study Procedure	12-Week Follow-Up EOS Visit	Early Termination during 12-Week Follow-Up[1]	Early Termination during Parts A, B, or C[1]	Unscheduled Visit before Rescue Treatment	Unscheduled Visit for Other Reasons
**Week (W)**	**12 weeks after EOT visit**				
**Day (D)**	**84 days after EOT visit**				
**Visit Window (Days [d])**	**±7 d**				
Concomitant medications/procedures[10]	X	X	X	X	X
**Efficacy:**					
DSQ eDiary[2]	←completed daily by patient→		X	X	
PGIC[3]			X	X	
PGIS[3]			X	X	
EoE Impact Questionnaire [4]			X	X	
EoE Symptom Questionnaire [4]			X	X	
TNSS [5]			X	X	
RQLQ(S)+12 [5]			X	X	
ACQ-5 [5]			X	X	
POEM [5]			X	X	
EoE-EREFS [6, 7]			X [7, 7a]	X [8]	
Endoscopy with biopsies (histology, IHC, RNA)[7]			X [7, 7a]	X [8]	
**Safety[10]:**					
Vital signs	X	X	X	X	
Height [9]			X		
Weight	X	X	X		
Physical examination			X		
ECG			X		
Adverse events	X	X	X	X	X
**Laboratory Testing[10]:**					
Hematology, Chemistry	X	X	X		
Pregnancy test	Urine	Urine	Urine		
Urinalysis	X	X	X		

Study Procedure	12-Week Follow-Up EOS Visit	Early Termination during 12-Week Follow-Up[1]	Early Termination during Parts A, B, or C[1]	Unscheduled Visit before Rescue Treatment	Unscheduled Visit for Other Reasons
**Week (W)**	**12 weeks after EOT visit**				
**Day (D)**	**84 days after EOT visit**				
**Visit Window (Days [d])**	**±7 d**				
**PK and ADA[10]:**					
PK Sample	X	X	X	X	
ADA sample	X	X	X	X	X
**Biomarkers:**					
Future Biomarker Serum/Plasma (optional)			X		

ADA = anti-drug antibody; DSQ = Dysphagia Symptom Questionnaire; ECG = electrocardiogram; EoE-EREFS = Eosinophilic Esophagitis-Endoscopic Reference Score; EOS = end of study (visit); EOT = end of treatment period; POEM = Patient-Oriented Eczema Measure; PRO = patient-reported outcome; PGA = physician global assessment; PGIC = Patient Global Impression of Change of Dysphagia; PGIS = Patient Global Impression of Severity of Dysphagia; PK = pharmacokinetic; RQLQ(S)+12 = Rhinoconjunctivitis Quality of Life Questionnaire for patients aged 12+ years; TNSS = Total Nasal Symptom Score

CONFIDENTIAL

VV-RIM-00127522-1.0 Approved - 05 Nov 2020 GMT-5:00

8.1.1. Footnotes for the Schedule of Events Tables

8.1.1.1. Footnotes for Table 1

1. For patients without a satisfactory prior endoscopy/biopsy (eg, histological criteria were not met, or the biopsy was not performed while patient was on at least 8 weeks of high-dose PPI treatment), the screening period will be extended for up to 12 weeks (day -85) to allow for at least 8 weeks of high-dose PPI treatment prior to the screening endoscopy/biopsies. For all other patients, the screening period will be shorter, with sufficient time to allow screening assessments and laboratory test results to be available prior to the baseline endoscopy/biopsies.
2. The endoscopy/EoE-EREFS/biopsy procedures should be performed after all other efficacy and safety assessments.
   a. The baseline endoscopy with biopsies should be performed at approximately day -21±7 days to allow for availability of the intraepithelial eosinophil count result from the central pathology laboratory prior to day 1. For patients without a satisfactory prior historical endoscopy/biopsy, the baseline endoscopy/biopsies must be performed after at least 8 weeks of high-dose PPI. Patients may be randomized as soon as their endoscopy/biopsy results are available and DSQ eDiary entries are completed.

      Note: Biopsy specimens from the stomach and/or duodenum will be obtained in all patients <18 years of age to rule out alternate etiologies. Stomach and/or duodenal biopsies will be obtained in adults only in the event of abnormal endoscopic findings (other than typical EoE findings) or clinical suspicion of alternate etiologies.

   b. For patients who receive rescue treatment during the double-blind treatment period, the endoscopy/EoE-EREFS/biopsy procedures will be performed prior to the initiation of rescue treatment. Patients undergoing endoscopy/biopsy prior to rescue will not undergo the scheduled endoscopy/biopsy at week 24

      Note: All randomized patients found to have stomach and/or duodenal abnormalities at baseline will have follow-up stomach and/or duodenal biopsies at week 24 and week 52.

   c. The EndoFLIP procedure to measure esophageal distensibility may be performed during the esophagogastroscopy procedures at selected North American sites in approximately 150 adult patients.
3. Assessments indicated for this week 24 (end of treatment) visit should be performed for all patients in Parts A and B. For patients who will enter Part C, there are additional events listed in week 24 visit in Table 2 for Part C.
4. Patients may be re-screened once if they fail the screening evaluation, unless the reason for screen failure is related to histologic or clinical disease severity inclusion criteria. The baseline endoscopy with biopsy and EoE-EREFS scoring will not be repeated for re-screened patients. These results will continue to be valid baseline data. Re-screening must occur within 6 months of the screen failure.

CONFIDENTIAL

VV-RIM-00127522-1.0 Approved - 05 Nov 2020 GMT-5:00

5. Patients and/or caregivers will be trained on administration of study drug at a minimum at visit 3/day 1
6. On scheduled in-clinic study visit days, study drug will be administered in the clinic (by the patient, site staff, or caregiver). Study drug will be provided for those doses scheduled to be administered at home before the next in-clinic visit. Doses of study drug administered at home should be administered 1 week after the prior dose of study drug. Study drug administration that occurs in clinic should occur per the Schedule of Events in Table 1 and Table 2. Patients will be closely monitored at the study site at visits 3 to 6 for a minimum of 30 minutes after the administration of study drug. In addition to the predose assessments, AEs and vital signs (body temperature, blood pressure, respiratory rate and heart rate) will be assessed at 30 minutes (±10 minutes) post-dose.
    a. If COVID-19 restrictions limit the availability of staff or the patient to have in-clinic visits, after visit 3/day 1 patients/caregivers may be dispensed study drug for at-home dosing. Additionally, if the visit 11/week 24 endoscopy with biopsies is delayed due to COVID-19 restrictions, extended blinded dosing of study drug is allowed until this visit can occur. If extended dosing of the study drug occurs beyond week 23, the clinic staff must contact the patient every 4 weeks (at a minimum) to collect any AE (including ISRs), any change in concomitant medications and/or procedures, and monthly pregnancy test results, if required.
7. Assessments will be performed and blood samples will be collected before the administration of study drug.
    a. If COVID-19 restrictions limit the availability of staff or the patient to have in-clinic visits, procedures/sample collection should occur at the next available in-clinic visit. Pregnancy testing must be performed as indicated (in-clinic or at-home) monthly (at a minimum) and results reported in a timely manner.
8. DSQ eDiary will be completed once daily by the patients after their last meal of the day but before they go to bed. Site personnel should conduct regular checks of patient eDiary compliance.
9. Patient Global Impression of Change (PGIC) of Dysphagia and Patient Global Impression of Severity (PGIS) of Dysphagia will be completed by the patient via electronic questionnaire at the indicated site visits. See Section 8.2.2.5 and Section 8.2.2.6.
10. EoE Impact Questionnaire and EoE Symptom Questionnaire will be completed by the patient via electronic questionnaire at the indicated site visits. See Section 8.2.2.3 and Section 8.2.2.4.
11. Total Nasal Symptom Score (TNSS) and Rhinoconjunctivitis Quality of Life Questionnaire for patients aged 12+ years [RQLQ(S)+12] will be administered only to patients with a documented history of allergic rhinitis; Asthma Control Questionnaire-5 (ACQ-5) will be administered only to patients with a documented history of asthma; Patient-Oriented Eczema Measure (POEM) will be administered only to patients with a documented history of AD. TNSS, POEM, ACQ-5 and RQLQ(S)+12 will be completed via electronic questionnaire and at the indicated site visits. The questionnaires will be administered only to the subset of patients who fluently speak the language in which the questionnaire is

presented (based on the availability of validated translations in participating countries). See Section 8.2.2.7, Section 8.2.2.8, Section 8.2.2.9, and Section 8.2.2.10.

12. EoE-EREFS should be completed by the investigator before biopsies for all patients are performed. For Part B enrolled patients, EoE-EREFS will be performed by both the investigators who perform the endoscopies as well as a centralized reading center, that will provide EoE-EREFS analysis and scoring from endoscopy imaging. For all endoscopies, the investigators will assess minor esophageal features including mucosal inflammatory and remodeling features.
13. At visits 3 through 6, vital signs (body temperature, blood pressure, respiratory rate, heart rate) should be taken predose and 30 minutes (±10 minutes) post-dose. Vital signs should be taken predose at all other indicated visits.
    a. If study drug administration is not possible in-clinic due to staff or patient availability due to COVID-19 restrictions, vital signs pre- and post-dose may not be performed.
14. For adolescent patients only.
15. Includes HIV Ab, HBsAg, HBsAb, HBcAb, HCV Ab, and TB. Tuberculosis testing will be performed on a country-by-country basis according to local guidelines if required by regulatory authorities or ECs.
16. Not required if post-menopausal status confirmed at screening. A negative result must be obtained prior to the randomization visit. In case of a positive urine test, the study treatment will be withheld and a serum pregnancy test to confirm the pregnancy should be performed as soon as possible. A confirmed pregnancy will lead to study drug discontinuation in all cases.
17. The EQ-5D-3L should be administered one time at either visit 1 or visit 3 for newly screened patients.
18. If COVID-19 restrictions limit the availability of staff or the patient to have in-clinic visits, the clinic staff should make every effort to contact the patient at the timepoint of the expected in-clinic visit to collect any AEs (including ISRs), any change in concomitant medications and/or procedures, and monthly pregnancy test results, if required.

8.1.1.2. Footnotes for Table 2

1. This visit is the same as the week 24 visit for Parts A and B (Table 1), and all other assessments indicated for week 24 of Parts A and B (Table 1) should be performed. All pre-dosing procedures (including endoscopy with biopsy) at visit 11/week 24 must be completed prior to administration of study drug for Part C extended active treatment.
2. Study assessments will be performed and blood samples will be collected prior to administration of study drug.
    a. If COVID-19 restrictions limit the availability of staff or the patient to have in-clinic visits, procedures/sample collection should occur at the next available in-clinic visit. Pregnancy testing must be performed as indicated (in-clinic or at-home) monthly (at a minimum) and results reported in a timely manner.

VV-RIM-00127522-1.0 Approved - 05 Nov 2020 GMT-5:00

3. Patients will be closely monitored at the study site at visits 11 (at a minimum) and 12, if possible, for a minimum of 30 minutes after the administration of study drug. In addition to predose assessments, AEs and vital signs (body temperature, blood pressure, respiratory rate, and heart rate) will be assessed at 30 minutes (±10 minutes) post-dose.
4. All pre-dosing procedures (including endoscopy with biopsy) at the scheduled visit 11/week 24 must occur prior to dosing with Part C / extended active treatment study drug. On scheduled in-clinic study visit days, study drug will be administered in the clinic (by the patient, site staff, or caregiver). Study drug will be provided for those scheduled doses to be administered at home before the next in-clinic visit. Doses of study drug administered at home should be administered 1 week after the prior dose of study drug. Study drug administration that occurs in clinic should occur per the Schedule of Events in Table 1 and Table 2.
    a. If COVID-19 restrictions limit the availability of staff or the patient to have in-clinic visits, after visit 11/week 24 patients/caregivers may be dispensed study drug for at-home dosing. Additionally, if the visit 19/ week 52 endoscopy with biopsies is delayed due to COVID-19 restrictions, extended dosing of study drug is allowed until this visit can occur. If extended dosing of the study drug occurs beyond week 51, the clinic staff must contact the patient every 4 weeks (at a minimum) to collect any AEs (including ISRs any change in concomitant medications and/or procedures, and monthly pregnancy test results, if required.
5. DSQ eDiary will be completed once daily by the patients after their last meal of the day but before they go to bed. Site personnel should conduct regular checks of patient eDiary compliance.
6. PGIC and PGIS will be completed by the patient via electronic questionnaire at the indicated site visits. See Section 8.2.2.5 and Section 8.2.2.6.
7. EoE Impact Questionnaire and EoE Symptom Questionnaire will be completed by the patient via electronic questionnaire at the indicated site visits. See Section 8.2.2.3 and Section 8.2.2.4.
8. TNSS and RQLQ(S)+12 will be administered only to patients with a documented history of allergic rhinitis; ACQ-5 will be administered only to patients with a documented history of asthma, and POEM will be administered only to patients with a documented history of AD. TNSS, POEM, ACQ-5 and RQLQ(S)+12 will be completed via electronic questionnaire and at the indicated site visits. The questionnaires will be administered only to the subset of patients who fluently speak the language in which the questionnaire is presented (based on the availability of validated translations in participating countries). See Section 8.2.2.7, Section 8.2.2.8, Section 8.2.2.9, and Section 8.2.2.10.
9. EoE-EREFS should be completed by the investigator before biopsies for all patients are performed. For Part B enrolled patients, EoE-EREFS will be performed by both the investigators who perform the endoscopies as well as a centralized reading center who will provide EoE-EREFS analysis and scoring from endoscopy imaging. For all endoscopies, the investigators will assess minor esophageal features including mucosal inflammatory and remodeling features.

VV-RIM-00127522-1.0 Approved - 05 Nov 2020 GMT-5:00

10. Endoscopy/EoE-EREFS/biopsy procedures should be performed after all other efficacy and safety assessments. The EndoFLIP procedure to measure esophageal distensibility may be performed during the esophagogastroscopy procedures at selected North American sites in approximately 150 adult patients.

    Note: All randomized patients found to have stomach and/or duodenal abnormalities at baseline will have follow-up stomach and/or duodenal biopsies at weeks 24 and 52.

    a. For patients who receive rescue treatment, endoscopy/EoE-EREFS/biopsy procedures will be performed prior to initiation of rescue treatment. Patients undergoing endoscopy/biopsy prior to rescue will not undergo the scheduled endoscopy/biopsy at weeks 24 and 52.
11. At visits 11 and 12, vital signs (body temperature, blood pressure, respiratory rate, heart rate) should be taken predose and 30 minutes (±10 minutes) post-dose. Only predose vital signs are required at subsequent visits.
12. For adolescents only
13. In case of a positive urine pregnancy test, the study treatment will be withheld and a serum pregnancy test to confirm the pregnancy should be performed as soon as possible. A confirmed pregnancy will lead to study drug discontinuation in all cases.
14. If COVID-19 restrictions limit the availability of staff or the patient to have in-clinic visits, the clinic staff should make every effort to contact the patient at the timepoint of the expected in-clinic visit to collect any AEs (including ISRs), any change in concomitant medications and/or procedures, and monthly pregnancy test results, if required.

8.1.1.3. Footnotes for Table 3

1. Patients who are withdrawn from study drug will be asked to complete the 12-week follow-up period and the end of study visit.
2. DSQ eDiary will be completed once daily by the patients after their last meal of the day but before they go to bed. Site personnel should conduct regular checks of patient eDiary compliance.
3. PGIC and PGIS will be completed by the patient via electronic questionnaire at the indicated site visits. See Section 8.2.2.5 and Section 8.2.2.6.
4. EoE Impact Questionnaire and EoE Symptom Questionnaire will be completed by the patient via electronic questionnaire at the indicated site visits. See Section 8.2.2.3 and Section 8.2.2.4.
5. TNSS and RQLQ(S)+12 will be administered only to patients with a documented history of allergic rhinitis; ACQ-5 will be administered only to patients with a documented history of asthma, and only in countries in which a valid translation is available; POEM will be administered only to patients with a documented history of AD. TNSS, POEM, ACQ-5 and RQLQ(S)+12 will be completed via electronic questionnaire and at the indicated site visits. See Section 8.2.2.7, Section 8.2.2.8, Section 8.2.2.9, and Section 8.2.2.10.

CONFIDENTIAL

VV-RIM-00127522-1.0 Approved - 05 Nov 2020 GMT-5:00

6. EoE-EREFS should be completed by the investigator before biopsies for all patients are performed. For Part B enrolled patients, EoE-EREFS will be performed by both the investigators who perform the endoscopies as well as a centralized reading center who will provide EoE-EREFS analysis and scoring from endoscopy imaging. For all endoscopies, the investigators will assess minor esophageal features including mucosal inflammatory and remodeling features.
7. Endoscopy/EoE-EREFS/biopsy procedures should be performed after all other efficacy and safety assessments. The EndoFLIP procedure to measure esophageal distensibility may be performed during the esophagogastroscopy procedures at selected North American sites in approximately 150 adult patients.

   Note: All randomized patients found to have stomach and/or duodenal abnormalities at baseline will have follow-up stomach and/or duodenal biopsies at weeks 24 and 52 or ET visit.

   a. For patients who receive rescue treatment during the double-blind treatment period, endoscopy/EoE-EREFS/biopsy procedures will be performed prior to initiation of rescue treatment. Rescue treatment should not be delayed if an endoscopy with biopsies cannot occur due to COVID-19 restrictions. Patients undergoing endoscopy/biopsy prior to rescue will not undergo the scheduled endoscopy/biopsy at weeks 24 and 52.
8. Endoscopy/EoE-EREFS/biopsy will be performed only if the Unscheduled Visit is for the purpose of administering rescue therapy.
9. For adolescents only.
10. If COVID-19 restrictions limit the availability of staff or the patient to have in-clinic visits, the clinic staff should make every effort to contact the patient at the timepoint of the expected in-clinic visit to collect any AEs (including ISRs), any change in concomitant medications and/or procedures, and monthly pregnancy test results, if required.

8.1.2. Early Termination Visit

Patients who are withdrawn from the study will be asked to return to the clinic for an ET visit consisting of the assessments described in Table 3.

8.1.3. Unscheduled Visits before Rescue Treatment

Before patients receive rescue treatment, efficacy assessments, PK and ADA sampling specified in Table 3 should be performed.

8.1.4. Unscheduled Visits for Other Reasons

All attempts should be made to keep patients on the study schedule. Unscheduled visits may be necessary to repeat testing following abnormal laboratory results, for follow-up of AEs, or for any other reason, as warranted.

VV-RIM-00127522-1.0 Approved - 05 Nov 2020 GMT-5:00

8.2. Study Procedures

8.2.1. Procedures Performed Only at the Screening/Baseline Visit

The following procedures will be performed for the sole purpose of determining study eligibility or characterizing the baseline population: serum follicle stimulating hormone (FSH) (for confirmation of menopausal status) and serology tests (HIV Ab, HBsAg, HBcAb, HCV Ab, and TB). Tuberculosis testing will be performed on a country-by-country basis according to local guidelines if required by regulatory authorities or ECs.

8.2.2. Efficacy Procedures

8.2.2.1. Endoscopy with EoE-EREFS and Biopsies, and Photographs

During the endoscopy procedure, EoE-EREFS and biopsies will be performed in the listed order. Training will be provided to study sites on performing this procedure. All biopsies performed during this study will be evaluated by a qualified central histology laboratory. Photographs and/or video may be taken as part of the endoscopic procedure and biopsy collection. For Part B enrolled patients, video imaging should be collected for EoE-EREFS analysis and scoring by a centralized reading center.

The endoscopy/EoE-EREFS/biopsy procedure will be performed per the Schedule of Events in Table 1, Table 2, and Table 3.

EoE-EREFS

The EoE esophageal characteristics will be analyzed based on the EoE-EREFS, a validated scoring system for inflammatory and remodeling features of disease using both overall scores and scores for each individual characteristic (Hirano, 2013). The proximal and distal esophageal regions will be scored separately; the score for each region ranges from 0 to 9 and the overall score ranges from 0 to 18. The major esophageal features include:

- Edema (absent, present)
- Rings (absent, mild, moderate, severe)
- Exudates (absent, mild, severe)
- Furrows (absent, mild, severe)
- Stricture (absent, present)

EoE-EREFS should be completed by the investigator before biopsies for all patients are performed. For Part B enrolled patients, EoE-EREFS will be performed by both the investigators who perform the endoscopies as well as a centralized reading center who will provide EoE-EREFS analysis and scoring from endoscopy imaging. The centralized reading will be used for analysis, if available. If the centralized reading is not available, the EoE-EREFS performed by the investigator will be used for analysis. A copy of the EoE-EREFS is provided in the study regulatory binders.

VV-RIM-00127522-1.0 Approved - 05 Nov 2020 GMT-5:00

In addition to the major features above, data for the following minor features will also be captured by the physician performing the endoscopy procedure:

- Crepe paper esophagus (mucosal fragility or laceration upon passage of diagnostic endoscope): absent, present
- Narrow caliber esophagus (reduced luminal diameter of the majority of the tubular esophagus): absent, present
- Stricture diameter

Mucosal changes associated with gastroesophageal reflux disease will also be recorded using the Los Angeles classification system for erosions (No Erosions or Grade A, B, C, or D).

Biopsies

Biopsies will be obtained by endoscopy at the second screening visit (visit 2, day -21 ± 7), week 24 and week 52 visits, and immediately prior to start of rescue medication or procedures during the double-blind treatment period. The screening endoscopy should be performed at a time during the screening period that will allow results to be available prior to day -1 for assessment of eligibility. A total of 9 mucosal pinch biopsies will be collected at each time point from 3 esophageal regions: 3 proximal, 3 mid, and 3 distal. Two samples from each region will be used for the histology (needed for study inclusion criteria, as well as endpoint assessment) and other tissue analyses (may include but not limited, to immunohistochemistry [IHC], RNA scope (in situ hybridization), and RNA sequencing). To participate in the study, patients must have a peak intraepithelial eosinophil count ≥15 eos/hpf (400×) in at least 2 of the 3 esophageal regions sampled. Any remaining tissue blocks will be banked for exploratory research. The third sample from each region will be processed for RNA analyses.

In addition, biopsy specimens from the stomach and/or duodenum will be obtained at visit 2 in all patients <18 years of age to rule out alternate etiologies of esophageal eosinophilia. Targeted, stomach and/or duodenal biopsies will be obtained in adults only in the event of abnormal endoscopic findings (other than typical EoE findings) or clinical suspicion of alternate etiologies. Gastric biopsy samples should include 2 samples from the antrum and 2 samples from the body. Duodenal biopsy samples should include 2 bulb samples and 2 from another portion of the duodenum. All randomized patients found to have stomach and/or duodenal abnormalities at baseline will have follow-up stomach and/or duodenal biopsies at week 24 and week 52.

Biopsy samples will be sent to a central pathology laboratory for processing and analysis. If required by the investigator institution, biopsy samples will be processed and analyzed by the local laboratory, and the processed specimen will be sent to the central pathology laboratory for central reading. These samples will be assessed for peak eos per hpf and EoE Grade Scores and Stage Scores will be assigned. EoE Grade and Stage Scores evaluate eight features: eosinophil density, basal zone hyperplasia, eosinophil abscesses, eosinophil surface layering, dilated intercellular spaces, surface epithelial alteration, dyskeratotic epithelial cells, and lamina propria fibrosis (absent/present). Additional analyses on these data may also be performed.

Assessment of lamina propria fibrosis may not be possible if the esophageal biopsy specimens do not contain adequate amounts of subepithelium lamina propria and then will not be included in the overall EoE Grade and Stage Scores. Severity (grade) and extent (stage) of abnormalities will be

VV-RIM-00127522-1.0 Approved - 05 Nov 2020 GMT-5:00

scored using a 4-point scale (0 normal; 3 maximum change). The endoscopy with biopsies procedure will be performed the same way at all scheduled visits.

Histology results will be interpreted by a pathologist at a central pathology reading center who will be blinded to the treatment assignment. Detailed instructions for biopsy sample collection and handling will be provided in the study regulatory binders.

EndoFLIP

A substudy of esopohageal distensibility utilizing the endolumenal functional lumen imaging probe (EndoFLIP, Medtronic, USA) may also be performed at selected North American sites in approximately 150 adult patients, with measurements taken as part of the esophagogastroscopy procedure performed at screening, week 24 and week 52. The EndoFLIP procedure must be performed before biopsies are collected. Procedural order should be: EREFS/imaging, EndoFLIP, then biopsies. The EndoFLIP device is a catheter-based procedure that measures the cross-sectional area at multiple sites along the esophagus with simultaneous intraluminal pressure recordings during volumetric distension of the esophagus. The analyses of cross sectional area versus pressure relationships of the esophagus allow for determination of esophageal compliance as well as the distensibility plateau. The distensibility plateau has been shown to be significantly reduced in adult patients with EoE compared to healthy controls (Kwiatek, 2011). Moreover, the esophageal distensibility has been associated with outcomes of both food impaction and need for esophageal dilation (Nicodème, 2013). Details of the EndoFLIP procedure will be provided in the study regulatory binders.

Photographs and/or Video

Photographs and/or video may be taken by the site as part of the endoscopic procedure and biopsy collection. A copy of these photographs and/or video will be requested from the study sites. Details for collecting and sending these photographs and/or video will be provided in the study regulatory binders. For Part B enrolled patients, video imaging should be collected for EoE-EREFS analysis and scoring by a centralized reading center.

8.2.2.2. Dysphagia Symptom Questionnaire (DSQ) - Patient-reported Outcome (PRO)

The DSQ is a validated PRO that has been used in clinical studies to measure the frequency and intensity of dysphagia (Hudgens, 2017). For patients who respond "No" to Question 1 ("Since you woke up this morning, did you eat solid food?"), a modification was made to the DSQ by asking a follow-up question to probe if patients avoided solid food due to their problems with swallowing solid food. This modified DSQ will be completed by the patient daily using an eDiary from screening through end of study or ET visit. A copy of the DSQ is provided in the study regulatory binders.

The DSQ uses a daily recall period and comprises 3 questions on the presence and severity of EoE dysphagia. All patients respond to questions 1 and 2 and are required to have eaten solid foods ('Yes' to question 1: "Since you woke up this morning, did you eat solid food?") in order to proceed with the questionnaire. If a patient answers "No" to question 1, the remaining items on the DSQ are not scored. Patients who respond "No" to question 2 ("Since you woke up this morning, has food gone down slowly or been stuck in your throat?") are given a score of zero, and do not go on to answer question 3 (the diary is recorded as completed for that day). Those who

respond ‘Yes’ to questions 1 and 2 move on to question 3, which is scored on a 5-point scale that infers severity of dysphagia based on the patient’s action to alleviate symptoms, ranging from no action to seeking medical attention. The DSQ scoring algorithm is therefore constructed from the responses to questions 2 and 3, to ensure that the final score is driven by the frequency and severity of dysphagia. To calculate the DSQ score, a minimum of 8 diary entries is required for each 14-day period to derive a standardized total score based on the cumulative scores through 14 days. DSQ scores can theoretically range from 0 to 84, with a lower score indicating less-frequent or less-severe dysphagia.

Although Question 4 related to pain was included in the DSQ, it is considered as a standalone item for exploratory analysis.

8.2.2.3. EoE Impact Questionnaire (EoE-IQ)

The EoE-IQ is a disease-specific measure of health-related QOL in EoE patients developed by the sponsor. The EoE-IQ measures EoE impact on emotional, social, work and school, and sleep aspect of a patient.

This assessment will be recorded by the patient using electronic questionnaire per the Schedule of Events in Table 1, Table 2, and Table 3. A copy of the EoE-IQ is provided in the study regulatory binders. If COVID-19 restrictions limit the availability of staff or the patient to have in-clinic visits, after visit 3/day 1, site staff should make every effort to conduct telephone interviews to complete these questionnaires. Site staff should conduct the telephone interviews on the date of scheduled site visit by following an interview guide provided by the sponsor. Patient responses from the interviewer administered questionnaires will be captured by the site staff on a paper-copy print-out of the questionnaire screenshots.

8.2.2.4. EoE Symptom Questionnaire

The EOE Symptom Questionnaire is a questionnaire measuring the frequency and severity of symptoms other than dysphagia and pain with swallowing. It is developed by the sponsor. The EoE Symptom Questionnaire will be completed by patients using electronic questionnaire per the Schedule of Events in Table 1, Table 2, and Table 3.

If COVID-19 restrictions limit the availability of staff or the patient to have in-clinic visits, after visit 3/day 1, site staff should make every effort to conduct telephone interviews to complete these questionnaires. Site staff should conduct the telephone interviews on the date of scheduled site visit by following an interview guide provided by the sponsor. Patient responses from the interviewer administered questionnaires will be captured by the site staff on a paper-copy print-out of the questionnaire screenshots.

8.2.2.5. Patient Global Impression of Change (PGIC) of Dysphagia

The PGIC is a one-item questionnaire that asks patients to provide the overall self-assessment of change of difficulty swallowing food. The PGIC will be completed by patients using electronic questionnaire per the Schedule of Events in Table 1, Table 2, and Table 3.

If COVID-19 restrictions limit the availability of staff or the patient to have in-clinic visits, after visit 3/day 1, site staff should make every effort to conduct telephone interviews to complete these questionnaires. Site staff should conduct the telephone interviews on the date of scheduled site

visit by following an interview guide provided by the sponsor. Patient responses from the interviewer administered questionnaires will be captured by the site staff on a paper-copy print-out of the questionnaire screenshots.

8.2.2.6. Patient Global Impression of Severity (PGIS) of Dysphagia

The PGIS is a one-item questionnaire that asks patients to provide the overall self-assessment of difficulty of swallowing food. The PGIS will be completed by patients using electronic questionnaire per the Schedule of Events in Table 1, Table 2, and Table 3.

If COVID-19 restrictions limit the availability of staff or the patient to have in-clinic visits, after visit 3/day 1, site staff should make every effort to conduct telephone interviews to complete these questionnaires. Site staff should conduct the telephone interviews on the date of scheduled site visit by following an interview guide provided by the sponsor. Patient responses from the interviewer administered questionnaires will be captured by the site staff on a paper-copy print-out of the questionnaire screenshots.

8.2.2.7. Total Nasal Symptom Score (TNSS)

The Total Nasal Symptom Score (TNSS), measured on a 0-9 scale, is a composite symptom assessment of congestion, itching/sneezing, and rhinorrhea (each graded on a 0-3 scale, 3 being severe). The TNSS will be administered only to patients with a documented history of allergic rhinitis and who fluently speak a language in which the questionnaire is presented (based on availability of translations in participating countries). The TNSS will be completed by patients per the Schedule of Events in Table 1, Table 2, and Table 3.

If COVID-19 restrictions limit the availability of staff or the patient to have in-clinic visits, after visit 3/day 1, site staff should make every effort to conduct telephone interviews to complete these questionnaires. Site staff should conduct the telephone interviews on the date of scheduled site visit by following an interview guide provided by the sponsor. Patient responses from the interviewer administered questionnaires will be captured by the site staff on a paper-copy print-out of the questionnaire screenshots.

8.2.2.8. Standardized Rhinoconjunctivitis Quality of Life Questionnaire for ages 12+ [RQLQ(S)+12]

Standardized Rhinoconjunctivitis Quality of Life Questionnaire for ages 12+ [RQLQ(S)+12] is a self-administered questionnaire to measure health-related QOL in those 12 years of age and above, as a result of perennial or seasonal allergic rhinitis. There are 28 items on the RQLQ(S) in 7 domains: activity limitation, sleep problems, nasal symptoms, eye symptoms, non-nasal/eye symptoms, practical problems, and emotional function. The RQLQ(S)+12 responses are based on a 7-point Likert scale with responses ranging from 0 (not troubled) to 6 (extremely troubled). The overall RQLQ(S)+12 score is the mean of all 28 responses and the individual domain scores are the means of the items in those domains. Higher scores indicated more health-related QOL impairment (lower scores were better). A change of 0.5 point or more in total score is considered to be clinically meaningful.

The RQLQ(S)+12 will be performed per the Schedule of Events in Table 1, Table 2, and Table 3.

VV-RIM-00127522-1.0 Approved - 05 Nov 2020 GMT-5:00

If COVID-19 restrictions limit the availability of staff or the patient to have in-clinic visits, after visit 3/day 1, site staff should make every effort to conduct telephone interviews to complete these questionnaires. Site staff should conduct the telephone interviews on the date of scheduled site visit by following an interview guide provided by the sponsor. Patient responses from the interviewer administered questionnaires will be captured by the site staff on a paper-copy print-out of the questionnaire screenshots.

8.2.2.9. Juniper Asthma Control Questionnaire (ACQ)

The 5-question version of the Juniper ACQ (ACQ-5) is a validated questionnaire to evaluate asthma control. The ACQ-5 score is the mean of the scores of the 5 items and ranges from 0 (totally controlled) to 6 (severely uncontrolled). Scores less than 1.0 reflect adequately controlled asthma, and scores 1.0 or greater reflect inadequately controlled asthma. Higher score indicates lower asthma control. The recommended change of 0.50 is a reasonable threshold to define a meaningful individual-level change. The ACQ-5 will be administered only to patients with a documented history of asthma and who fluently speak a language in which the questionnaire is presented (based on availability of validated translations in participating countries). The ACQ-5 will be completed by patients using electronic questionnaire per the Schedule of Events in Table 1, Table 2, and Table 3.

If COVID-19 restrictions limit the availability of staff or the patient to have in-clinic visits, after visit 3/day 1, site staff should make every effort to conduct telephone interviews to complete these questionnaires. Site staff should conduct the telephone interviews on the date of scheduled site visit by following an interview guide provided by the sponsor. Patient responses from the interviewer administered questionnaires will be captured by the site staff on a paper-copy print-out of the questionnaire screenshots.

8.2.2.10. Patient-Oriented Eczema Measure (POEM)

The POEM is a 7-item, validated questionnaire used in clinical practice and clinical trials to assess disease symptoms in children and adults with AD (Charman, 2004). The format is a response to 7 items (dryness, itching, flaking, cracking, sleep loss, bleeding, and weeping) using a 5-point scale, based on frequency of occurrence during the past week. The possible scores for each question were: 0 (no days), 1 (1 to 2 days), 2 (3 to 4 days), 3 (5 to 6 days), and 4 (every day), with a composite scoring system of 0 to 28; a higher score is indicative of more severe AD.

The following POEM banding scores have been established (Charman, 2004): 0 to 2=clear or almost clear; 3 to 7=mild eczema; 8 to 16=moderate eczema; 17 to 24=severe eczema; and 25 to 28=very severe eczema.

The POEM will be administered only to patients with a documented history of AD and who fluently speak a language in which the questionnaire is presented (based on availability of validated translations in participating countries). The POEM will be completed by patients per the Schedule of Events in Table 1, Table 2, and Table 3.

If COVID-19 restrictions limit the availability of staff or the patient to have in-clinic visits, after visit 3/day 1, site staff should make every effort to conduct telephone interviews to complete these questionnaires. Site staff should conduct the telephone interviews on the date of scheduled site visit by following an interview guide provided by the sponsor. Patient responses from the

interviewer administered questionnaires will be captured by the site staff on a paper-copy print-out of the questionnaire screenshots.

8.2.2.11. European Quality of Life 5-Dimensional Scale (EQ-5D)

The European Quality of Life 5-dimension (EQ-5D) scale is a standardized questionnaire used to assess health status (Rabin, 2014) (Brooks, 1996). It consists of a descriptive system and the EQ visual analogue scale (EQ VAS). The EQ-5D-3L (3-level) descriptive system comprises of the following 5 dimensions: mobility, self-care, usual activities, pain/discomfort, and anxiety/depression. For each dimension, patients select one of 3 levels: no problems, some problems, and extreme problems. The EQ VAS records the patient's self-rated health on a vertical visual analogue scale where the endpoints are labelled 'Best imaginable health state' and 'Worst imaginable health state'. The EQ-5D-3L will be completed by patients per the Schedule of Events in Table 1.

8.2.3. Safety Procedures

8.2.3.1. Vital Signs

Vital signs, including heart rate, blood pressure, respiratory rate, and body temperature will be collected predose and 30 minutes post-dose at time points listed in Table 1, Table 2, and Table 3. Heart rate and blood pressure will be measured with the patient in a sitting position, after the patient has rested comfortably for at least 5 minutes.

8.2.3.2. Physical Examination

A thorough and complete physical examination will be performed at time points according to Table 1, Table 2, and Table 3. Care should be taken to examine and assess any abnormalities that may be present, as indicated by the patient's medical history.

8.2.3.3. Body Weight and Height

Body weight and height will be measured at time points according to Table 1, Table 2, and Table 3.

8.2.3.4. Electrocardiogram

Electrocardiograms (ECG) will be performed before blood is drawn during visits requiring blood draws. A standard 12-lead ECG will be performed predose at time points listed in Table 1, Table 2, and Table 3. Heart rate will be recorded from the ventricular rate and the PR, QRS, and QT (identify QTcB or QTcF) intervals will be recorded. The ECG strips or reports will be retained with the source documentation, and the results will be documented in the electronic case report form (eCRF).

Electrocardiogram results will be interpreted by a central reading center. Instructions for performing the assessment and transmitting ECG data are provided in the study regulatory binders.

8.2.3.5. Laboratory Testing

Hematology, chemistry, urinalysis, and pregnancy testing samples will be analyzed by a central laboratory. Samples will be collected predose at time points listed in Table 1, Table 2, and Table 3.

CONFIDENTIAL

VV-RIM-00127522-1.0 Approved - 05 Nov 2020 GMT-5:00

Detailed instructions for blood sample collection are in the laboratory manual provided to study sites. Tests will include:

Blood Chemistry

Sodium	Total protein, serum	Total and indirect bilirubin
Potassium	Creatinine	Total cholesterol
Chloride	Blood urea nitrogen (BUN)	Low-density lipoprotein (LDL)
Carbon dioxide	AST	High-density lipoprotein (HDL)
Calcium	ALT	Triglycerides
Glucose	Alkaline phosphatase	Uric acid
Albumin	Lactate dehydrogenase (LDH)	Creatine phosphokinase (CPK)[1]
	Estimated glomerular filtration rate (eGFR)	

[1] CPK isoenzymes will be measured when CPK >5 × ULN

Hematology

Hemoglobin	Differential:
Hematocrit	Neutrophils
Red blood cells (RBCs)	Lymphocytes
White blood cells (WBCs)	Monocytes
Red cell indices	Basophils
Platelet count	Eosinophils

Urinalysis

Microscopic analysis will only be done in the event of abnormal dipstick results.

Color	Glucose	RBC
Clarity	Blood	Hyaline and other casts
pH	Bilirubin	Bacteria
Specific gravity	Leukocyte esterase	Epithelial cells
Ketones	Nitrite	Crystals
Protein	WBC	Yeast

Other Laboratory Tests

- Pregnancy testing will be performed for all women of childbearing potential. Serum or urine pregnancy testing will be performed at time points listed in Table 1, Table 2, and Table 3. A serum FSH test will be performed if menopausal status is in question.
- The following tests will be performed at screening: HIV, HBsAg, HBsAb, HBcAb, hepatitis C Ab, TB (will be performed on a country-by-country basis according to local guidelines if required by regulatory authorities or ethics boards), and alcohol and drug screen test.

Abnormal Laboratory Values and Laboratory Adverse Events

All laboratory values must be reviewed by the investigator or authorized designee.

Significantly abnormal test results that occur after start of treatment must be repeated to confirm the nature and degree of the abnormality. When necessary, appropriate ancillary investigations should be initiated. If the abnormality fails to resolve or cannot be explained by events or conditions unrelated to the study medication or its administration, the Medical/Study Director must be consulted.

The clinical significance of an abnormal test value, within the context of the disease under study, must be determined by the investigator.

Criteria for reporting laboratory values as an AE are provided in Section 9.4.5.

8.2.4. Drug Concentration and Measurements

Samples for functional dupilumab concentration in serum will be collected predose at visits listed in Table 1, Table 2, and Table 3. Detailed instructions for blood sample collection are included in the laboratory manual provided to study sites

Any unused samples may be used for exploratory biomarker research.

8.2.5. Anti-Drug Antibody Measurements and Samples

Samples for ADA assessment will be collected predose at time points listed in Table 1, Table 2, and Table 3. Detailed instructions for blood sample collection are included in the laboratory manual provided to study sites.

Any unused samples may be used for exploratory biomarker research.

8.2.6. Pharmacodynamic and Exploratory Biomarker Procedures

In this study, research assessments will be performed to explore EoE, how dupilumab may modify the underlying disease process in EoE, type 2 inflammation, and predictors of dupilumab safety and efficacy.

Samples for eotaxin-3 (heparinized plasma); serum TARC, total IgE, and allergen-specific IgE and IgG4s will be collected at time points according to Table 1 and Table 2. The biomarkers studied are believed to be relevant to the pathophysiology of EoE, response to treatment (ie, assessment of type 2 inflammation) and baseline predictors of response and dupilumab mechanism of action.

8.2.6.1. EoE Diagnostic Panel and Type 2 Inflammation Transcriptomics

The differential gene expression profiles of esophageal biopsies of EoE patients compared to healthy controls is the EoE disease transcriptome (Sherrill, 2014). This disease gene expression signature was further refined to a smaller gene set to be used as an EoE diagnostic panel (EDP) (Dellon, 2017). A gene signature representing type 2 inflammation has been curated from the literature, preclinical experiments performed at Regeneron, and dupilumab response signatures from atopic dermatitis and a phase 2 study of EoE (Regeneron unpublished data). The gene lists comprising the EDP and type 2 transcriptomes can be found in the statistical analysis plan (SAP).

VV-RIM-00127522-1.0 Approved - 05 Nov 2020 GMT-5:00

In a phase 2 study of EoE (R668-EE-1324), dupilumab significantly decreased the disease, EDP, and type 2 transcriptome signatures (Regeneron unpublished data).

Normalized Enrichment Score (NES) reflects the degree to which the activity level of a set of transcripts is overrepresented at the extremes (top or bottom) of the entire ranked list of transcripts within a sample and is normalized by accounting for the number of transcripts in the set (Subramanian, 2005) (Barbie, 2009). NES scores will be calculated for each transcriptome signature for each sample for statistical analyses, collected as part of procedures in Table 1, Table 2, and Table 3.

8.2.6.2. Type 2 Inflammatory and Disease-Related Circulating Biomarkers

Eotaxin-3, Thymus and Activation Regulated Chemokine (TARC), and IgE (total and specific) are measures of type 2 inflammation, and are known pharmacodynamic markers for dupilumab in AD, asthma, and nasal polyposis.

8.2.6.2.1. Eotaxin-3

Eotaxin-3 (also known as CCL26), an eosinophil chemokine, is up-regulated in esophageal mucosa of EoE patients relative to controls (Blanchard, 2006), and variants in the gene have been associated with disease risk. Eotaxin-3 mRNA expression in skin is down-regulated by dupilumab in AD (Hamilton, 2014). Circulating concentrations have been shown to decrease in asthma and nasal polyposis patients treated with dupilumab. Eotaxin-3 will be measured in heparinized plasma from samples collected at time points indicated in Table 1 and Table 2. Modulation of eotaxin-3 is an exploratory measure. Data analysis will be described in the SAP and the results will be provided in the clinical study report (CSR).

8.2.6.2.2. Thymus and Activation Regulated Chemokine (TARC)

TARC is a type 2 chemokine for Th2 cells and other inflammatory cells expressing the CCR4 receptor. TARC expression is regulated by IL4 and IL13, and rapidly decreases upon dupilumab treatment in AD, asthma, and nasal polyps. Serum TARC will be measured at time points indicated in Table 1 and Table 2. Modulation of TARC is an exploratory measure. Data analysis will be described in the SAP and the results will be provided in the CSR.

8.2.6.2.3. Total and Allergen-Specific IgEs

IL4 and IL13 regulate B cell class switching to, as well as production of, IgE. Dupilumab has been shown to suppress IgE (total and allergen-specific) in AD, asthma, and nasal polyposis patients. Serum concentrations of IgE (total and a panel of allergen-specific) will be measured at time points indicated in Table 1. Modulation of IgE is an exploratory measure. Data analysis will be described in the SAP and the results will be provided in the CSR.

8.2.6.2.4. Allergen-Specific IgG4s

Allergen-specific IgG4s have been found to be elevated in esophageal tissue and in circulation of EoE patients. Because the factors driving histological abnormalities and symptoms are unclear, IgG4 has been proposed as a pathological driver in the disease (Clayton, 2014) (Wright, 2016). To evaluate the potential role of IgG4 in EoE pathogenesis, as well as whether or not dupilumab

VV-RIM-00127522-1.0 Approved - 05 Nov 2020 GMT-5:00

modulates these markers, a panel of serum allergen-specific IgG4s will be measured in samples collected at times indicated in Table 1 and Table 2. Modulation of allergen-specific IgG4s is an exploratory measure. Data analysis will be described in the SAP and results will be provided in the CSR.

8.2.7. Future Biomedical Research (FBR) Serum/Plasma (Optional)

Patients/parents or legal guardians who agree to participate in the future biomedical research (FBR) substudy will be required to consent to this optional substudy before FBR samples are collected and banked in long-term storage. The unused biomarker samples for study-related research, as well as unused PK and ADA samples, will be stored for up to 15 years after the final date of the database lock. The FBR samples may be utilized for future biomedical research of EoE and related diseases, and the effects of dupilumab on target pathway modulation. Additional samples will be collected for future biomedical research according to Schedule of Events in Table 1, Table 2, and Table 3. After 15 years, any residual samples will be destroyed. The results of these future biomedical research analyses will not be presented in the CSR. Patients are not required to participate in the FBR substudy in order to enroll in the primary study.

8.2.7.1. Genomics Substudy (Optional)

Patients/parent or legal guardians who agree to participate in the genomics substudy will be required to consent to this optional substudy before collection of the samples. Patients are not required to participate in the genomics substudy in order to enroll in the primary study.

Whole blood samples for DNA extraction should be collected on day 1/baseline (predose), but can be collected at a later study visit. Whole blood samples for RNA extraction will be collected at time points according to Table 1.

DNA and blood RNA samples will be collected for the pharmacogenomics analyses to understand the genetic determinants of efficacy and safety associated with the treatments in this study, and the molecular basis of EoE and related diseases. These samples will be single-coded as defined by the International Council on Harmonisation (ICH) guideline E15. Samples will be stored for up to 15 years after the final date of the database lock.

The purpose of the pharmacogenomic analyses is to identify genomic associations with clinical or biomarker response to dupilumab, other EoE clinical outcome measures, and possible AEs. In addition, associations between genomic variants and prognosis or progression of EoE as well as related allergic/atopic diseases may also be studied. These data may be used or combined with data collected from other studies to identify and validate genomic markers related to the study drug or EoE and related diseases.

Analyses may include sequence determination or single nucleotide polymorphism studies of candidate genes and surrounding genomic regions. Other methods, including whole-exome sequencing, whole-genome sequencing, DNA copy number variation, and transcriptome sequencing (or other methods for quantitating RNA expression) may also be performed. The list of methods may be expanded to include novel methodology that may be developed during the course of this study or sample storage period. Results from the genomic analyses will not be reported in the CSR.

CONFIDENTIAL

VV-RIM-00127522-1.0 Approved - 05 Nov 2020 GMT-5:00

9. SAFETY DEFINITIONS, REPORTING, AND MONITORING

9.1. Obligations of Investigator

The investigator must promptly report to the Institutional Review Board (IRB)/EC all unanticipated problems involving risks to patients/subjects, according to local regulations. This may include death from any cause and all SAEs related to the use of the study drug. It is recommended that all SAEs be reported to the IRB/EC, according to local regulations.

9.2. Obligations of Sponsor

During the course of the study, the sponsor will report in an expedited manner all SAEs that are both unexpected and at least reasonably related to the study drug (SUSAR), to the health authorities, ECs/IRBs as appropriate, and to the investigators (in a blinded manner).

Any AE not listed as an expected event in the Reference Safety Information section of the Investigator's Brochure will be considered as unexpected. Any worsening of or new onset of symptoms related to EoE during the screening period prior to study drug administration will be considered expected.

At the completion of the study, the sponsor will report all safety observations made during the conduct of the trial in the CSR to health authorities and ECs/IRBs as appropriate.

9.3. Definitions

9.3.1. Adverse Event

An AE is any untoward medical occurrence in a patient administered a study drug which may or may not have a causal relationship with the study drug. Therefore, an AE is any unfavorable and unintended sign (including abnormal laboratory finding), symptom, or disease which is temporally associated with the use of a study drug, whether or not considered related to the study drug.

An AE also includes any worsening (ie, any clinically significant change in frequency and/or intensity) of a pre-existing condition that is temporally associated with the use of the study drug.

9.3.2. Serious Adverse Event

An SAE is any untoward medical occurrence that at any dose:

- Results in **death** – includes all deaths, even those that appear to be completely unrelated to study drug (eg, a car accident in which a patient is a passenger).
- Is **life-threatening** – in the view of the investigator, the patient is at immediate risk of death at the time of the event. This does not include an AE that had it occurred in a more severe form, might have caused death.
- Requires in-patient **hospitalization** or **prolongation of existing hospitalization**. In-patient hospitalization is defined as admission to a hospital (any duration) or an emergency room visit for longer than 24 hours. Prolongation of existing hospitalization is defined as a hospital stay that is longer than was originally anticipated for the event,

VV-RIM-00127522-1.0 Approved - 05 Nov 2020 GMT-5:00

or is prolonged due to the development of a new AE as determined by the investigator or treating physician.

- Results in persistent or significant **disability/incapacity** (substantial disruption of one's ability to conduct normal life functions).
- Is a **congenital anomaly/birth defect**
- Is an **important medical event** – Important medical events may not be immediately life-threatening or result in death or hospitalization, but may jeopardize the patient or may require intervention to prevent one of the other serious outcomes listed above (eg, intensive treatment in an emergency room or at home for allergic bronchospasm; blood dyscrasias or convulsions that do not result in hospitalization; or development of drug dependency or drug abuse).

Criteria for reporting SAEs must be followed for these events. See Section 9.4 for more information on recording and reporting SAEs.

9.3.3. Adverse Events of Special Interest

An adverse event of special interest (AESI; serious or non-serious) is one of scientific and medical concern specific to the sponsor's product or program, for which ongoing monitoring and rapid communication by the investigator to the sponsor can be appropriate. Such an event might warrant further investigation in order to characterize and understand it. Depending on the nature of the event, rapid communication by the trial sponsor to other parties (eg, regulators) might also be warranted (Section 9.4.3).

9.4. Recording and Reporting Adverse Events

9.4.1. Adverse Events

The investigator (or designee) will record all AEs that occur from the time the informed consent is signed until the end of study. Refer to the study regulatory binders for the procedures to be followed.

Information on follow-up for AEs is provided in Section 9.4.6. Laboratory, vital signs, or ECG abnormalities are to be recorded as AEs as outlined in Section 9.4.5.

9.4.2. Serious Adverse Events

All SAEs, regardless of assessment of causal relationship to study drug, must be reported to the sponsor (or designee) within 24 hours. Refer to the study regulatory binders for the procedure to be followed.

Information not available at the time of the initial report must be documented in a follow-up report. Substantiating data such as relevant hospital or medical records and diagnostic test reports may also be requested.

VV-RIM-00127522-1.0 Approved - 05 Nov 2020 GMT-5:00

In the event the investigator is informed of an SAE after the patient completes the study, the following will apply:

- SAE with an onset within 30 days of the end of study or within 84 days (12 weeks) of last study drug administration if the patient early terminated from the study – the SAE will be reported to the sponsor. The investigator should make every effort to obtain follow-up information on the outcome until the event is considered chronic and/or stable.
- SAE with an onset day greater than 30 days from the end of study or ET visit – only fatal SAEs and those deemed by the investigator to be drug-related SAEs will be reported to the sponsor. The investigator should make every effort to obtain follow-up information on the outcome of a drug-related SAE until the event is considered chronic and/or stable.

9.4.3. Other Events that Require Accelerated Reporting to Sponsor

The following events also require reporting to the sponsor (or designee) within 24 hours of learning of the event:

Symptomatic Overdose of Study Drug: Accidental or intentional overdose of at least 2 times the intended dose of study drug within the intended therapeutic window, if associated with an AE.

Pregnancy: Although pregnancy is not considered an AE, it is the responsibility of the investigator to report to the sponsor (or designee), within 24 hours of identification, any pregnancy occurring in a female study patient, during the study or within 12 weeks of the last dose of study drug. Any complication of pregnancy affecting a female study patient and/or fetus and/or newborn that meets the SAE criteria must be reported as an SAE. Outcome for all pregnancies should be reported to the sponsor.

Adverse Events of Special Interest: All AESI, serious and non-serious, must be reported within 24 hours of identification using the same reporting process as for SAE reporting, per Section 9.4.2. Adverse events of special interest for this study include the following:

- Anaphylactic reactions
- Systemic hypersensitivity reactions
- Helminthic infections
- Any severe type of conjunctivitis or blepharitis
- Keratitis
- Clinically symptomatic eosinophilia (or eosinophilia associated with clinical symptoms)
- Severe injection site reactions
- Herpes simplex infection

VV-RIM-00127522-1.0 Approved - 05 Nov 2020 GMT-5:00

- Arthralgia

Refer to the study manual for the procedures to be followed.

9.4.4. Reporting Adverse Events Leading to Withdrawal from the Study

All AEs that lead to a patient's withdrawal from the study must be reported to the sponsor's Medical/Study Director within 30 days.

Refer to the study regulatory binders for the procedures to be followed.

9.4.5. Abnormal Laboratory, Vital Signs, or Electrocardiogram Results

The criteria for determining whether an abnormal objective test finding should be reported as an AE include:

- The test result is associated with accompanying symptoms, and/or
- The test result requires additional diagnostic testing or medical/surgical intervention, and/or
- The test result leads to a change in dosing (outside of protocol-stipulated dose adjustments), discontinuation from the study, significant additional concomitant drug treatment, or other therapy

Contact the Medical/Study Director in the event the investigator feels that an abnormal test finding should be reported as an AE, although it does not meet any of the above criteria.

Repeating an abnormal test, in the absence of any of the above conditions, does not constitute an AE. Any abnormal test result that is determined to be an error does not require reporting as an AE.

Evaluation of severity of laboratory abnormalities will be assessed according to the scale outlined in Section 9.5.1.

9.4.6. Follow-up

Adverse event information will be collected until the patient's last study visit.

Serious adverse event information will be followed up until the event is resolved or considered chronic and/or stable.

9.5. Evaluation of Severity and Causality

9.5.1. Evaluation of Severity

The severity of AEs will be graded according to the following scale:

Mild: Does not interfere in a significant manner with the patient's normal functioning level. It may be an annoyance. Prescription drugs are not ordinarily needed for relief of symptoms, but may be given because of personality of the patient.

Moderate: Produces some impairment of functioning but is not hazardous to health. It is uncomfortable or an embarrassment. Treatment for symptom may be needed.

VV-RIM-00127522-1.0 Approved - 05 Nov 2020 GMT-5:00

Severe: Produces significant impairment of functioning or incapacitation and is a definite hazard to the subject's health. Treatment for symptom may be given and/or patient hospitalized.

If a laboratory value is considered an AE, its severity should be based on the degree of physiological impairment the value indicates.

Injection Site Reactions

The severity of ISRs will be graded according to the following scale (semi-colon indicates "or" within description of grade):

Mild: Pain that does not interfere with activity; mild discomfort to touch; <5 cm of erythema or induration that does not interfere with activity

Moderate: Pain that requires repeated use of non-narcotic pain reliever >24 hours or interferes with activity; discomfort with movement; 5.1 cm to 10 cm erythema or induration or induration that interferes with activity

Severe: Pain that requires any use of narcotic pain reliever or that prevents daily activity; significant discomfort at rest; >10 cm erythema or induration; prevents daily activity; requires ER visit or hospitalization; necrosis or exfoliative dermatitis

9.5.2. Evaluation of Causality

Relationship of Adverse Events to Study Drug:

The relationship of AEs to study drug will be assessed by the blinded investigator, and will be a clinical decision based on all available information. The following question will be addressed:

Is there a reasonable possibility that the AE may have been caused by the study drug?

The possible answers are:

Not Related: There is no reasonable possibility that the event may have been caused by the study drug.

Related: There is a reasonable possibility that the event may have been caused by the study drug.

The investigator should justify the causality assessment of each SAE.

A list of factors to consider when assessing the relationship of AEs to study drug is provided below. Please note that this list is not exhaustive.

Is there a reasonable possibility that the event may have been caused by the study drug?

No:

- Due to external causes such as environmental factors or other treatment(s) being administered
- Due to the patient's disease state or clinical condition
- Do not follow a reasonable temporal sequence following the time of administration of the dose of study drug
- Do not reappear or worsen when dosing with study drug is resumed

- Are not a suspected response to the study drug based upon preclinical data or prior clinical data

Yes:

- Could not be explained by environmental factors or other treatment(s) being administered
- Could not be explained by the patient's disease state or clinical condition
- Follow a reasonable temporal sequence following the time of administration of the dose of study drug
- Resolve or improve after discontinuation of study drug
- Reappear or worsen when dosing with study drug
- Are known or suspected to be a response to the study drug based upon preclinical data or prior clinical data

Relationship of Adverse Events to Study Procedure:

The relationship of AEs to study procedure (eg, endoscopy or biopsy) will be assessed by the investigator, and will be a clinical decision based on all available information. The following question will be addressed:

Is there a reasonable possibility that the AE may have been caused by the procedure?

The possible answers are:

Not Related: There is no reasonable possibility that the event may have been caused by the procedure.

Related: There is a reasonable possibility that the event may have been caused by the procedure.

The sponsor will request information to justify the causality assessment of SAEs, as needed.

9.6. Safety Monitoring

The investigator will monitor the safety of study patient at his/her site(s) as per the requirements of this protocol and consistent with current Good Clinical Practice (GCP). Any questions or concerns should be discussed with the sponsor in a timely fashion. The sponsor will monitor the safety data from across all study sites. The Medical/Study Director will have primary responsibility for the emerging safety profile of the compound, but will be supported by other departments (eg, Pharmacovigilance and Risk Management (PVRM); Biostatistics and Data Management). Safety monitoring will be performed on an ongoing basis (eg, individual review of SAEs) and on a periodic cumulative aggregate basis.

In addition, the DMC will conduct safety reviews for all dupilumab studies (see details in Section 5.3.1).

9.7. Investigator Alert Notification

Regeneron (or designee) will inform all investigators participating in this clinical trial, as well as in any other clinical trial using the same investigational drug, of any SAE that meets the relevant requirements for expedited reporting (an AE that is serious, unexpected based on the reference safety information in the Investigator's Brochure, and has a reasonable suspected causal relationship to the study drug).

10. STATISTICAL PLAN

This section provides the basis for the SAPs for the study. The part-specific SAP(s) may be revised during the study to accommodate amendments to the clinical study protocol and to make changes to adapt to unexpected issues in study execution and data that may affect the planned analyses. The final SAP(s) will be issued before the database is locked for respective part.

Analysis variables are listed in Section 4.

Results from different parts of this study will be summarized and/or analyzed separately unless otherwise specified.

10.1. Statistical Hypothesis

For comparisons of each of the 2 dupilumab treatment groups to placebo, the following hypotheses of the co-primary endpoints will be tested, where p_d is the true proportion of patients achieving peak esophageal intraepithelial eosinophil count of ≤6 eos/hpf at week 24 and μ_d is the true mean change from baseline in the DSQ total score at week 24 in each of the dupilumab group; and p_p and μ_p are the corresponding true values in the placebo group.

- Null hypothesis (H_0): $p_p = p_d$ or $\mu_p = \mu_d$, ie, the proportion of patients achieving peak esophageal intraepithelial eosinophil count of ≤6 eos/hpf at week 24 **OR** the mean change from baseline in the DSQ total score at week 24 is the same between the dupilumab group and the placebo group.
- Alternative hypothesis (H_1): $p_p \neq p_d$ and $\mu_p \neq \mu_d$, ie, the proportion of patients achieving peak esophageal intraepithelial eosinophil count of ≤6 eos/hpf at week 24 **AND** the mean change from baseline in the DSQ total score at week 24 is different between the dupilumab group and the placebo group.

10.2. Justification of Sample Size

The assumptions used in sample size calculations for Part A are based on results from a phase 2 study of dupilumab (R668-EE-1324) and reported data of a phase 2 study of budesonide (Dellon, 2017) in patients with EoE as follows:

- In the study of dupilumab, a treatment difference of 65.2% was observed in the proportion of patients achieving peak esophageal intraepithelial eosinophil count of ≤6 eos/hpf after 12 weeks of treatment (placebo 0% vs. dupilumab 65.2%). DSQ was not assessed in this study. The overall dropout rate was 8.5% during the 12-week treatment period.

VV-RIM-00127522-1.0 Approved - 05 Nov 2020 GMT-5:00

- In the study of budesonide oral suspension, a treatment difference of 36% was observed in the proportion of patients achieving peak esophageal intraepithelial eosinophil count of ≤6 eos/hpf after 12 weeks of treatment (placebo 3% vs. budesonide 39%). The mean (standard deviation [SD]) of change from baseline in DSQ total score at week 12 was -7.5 (10.7) in placebo and -14.3 (13.0) in the budesonide group, corresponding to a treatment group difference of -6.8. The overall dropout rate was 6.5% during the 12-week treatment period.

With the consideration that a higher dropout rate may be observed for a longer treatment period, a dropout rate of 15% is assumed for all sample size calculations provided below.

Part A

The planned sample size for Part A is approximately 40 patients in each treatment group such that for the comparison of each dupilumab dose regimen to placebo:

- This sample size will yield >99% power to detect a treatment difference of 62% in the proportion of patients achieving peak esophageal intraepithelial eosinophil count of ≤6 eos/hpf at week 24 (placebo 3% vs. dupilumab 65%) at a 2-sided significance level of 5% using Fisher's exact test.
- With respect to the treatment group difference in the mean change from baseline in DSQ total score, assuming a common SD of 13.0, this sample size is expected to generate a 95% confidence interval whose half-width is 5.7. If the true treatment difference is -9.0 points, the statistical power for the co-primary endpoint of DSQ will be 80% using a two-sample t-test.

Part B

The study includes two double-blind placebo-controlled randomized parts: Part A and Part B. An objective of Part A was to evaluate the Part B sample size. Part A database lock occurred on 20 May 2020 and the study results for the co-primary endpoints from Part A are below:

- The proportion of patients achieving peak esophageal intraepithelial eosinophil count of ≤6 eos/hpf at 24 weeks was 5.1% in placebo group and 59.5% in dupilumab 300mg QW group with a treatment difference of 55.4%
- The mean (standard deviation [SD]) of change from baseline in DSQ total score at week 24 was -9.6 (15.2) in placebo and -21.9 (14.6) in the dupilumab 300mg QW group with a treatment group difference of -12.3.

Based on the Part A study results, the planned sample size for Part B is approximately 70 patients in each treatment group such that for the comparison of each dupilumab dose regimen to placebo:

- This sample size will yield >99.9% power to detect a treatment difference of 55.4% in the proportion of patients achieving peak esophageal intraepithelial eosinophil count of ≤6 eos/hpf at week 24 between placebo (5.1%) and each dupilumab treatment group (59.5%) at a 2-sided significance level of 5% using Fisher's exact test.
- This sample size will provide >99.9% power to detect a treatment difference of -12.3 points in the mean change from baseline in the total DSQ score to week 24 at a 2-sided significance level of 5% using a two-sample t-test, assuming a common SD of 15.0.

Therefore, the sample size of 70 patients/arm will provide an overall power of >99.9% (99.9% × 99.9%) for the co-primary endpoints, assuming no negative correlation between the two endpoints. In Part B, the same treatment effect for the two 300 mg dupilumab dose regimens (ie, QW and Q2W) is assumed.

Sample size calculations were made using nQuery Advisor 7.0.

10.3. Analysis Sets

10.3.1. Efficacy Analysis Sets

Part A and Part B

For Part A and Part B, the efficacy endpoints will be analyzed using the study part-specific full analysis set (FAS) that includes all randomized patients in the corresponding study part. Efficacy analyses for these 2 parts will be based on the treatment allocated by the randomization and carried out separately for each part. Analysis on the FAS will be considered to be primary.

The study-part specific per protocol set (PPS) will include all patients in the corresponding FAS except for those who are excluded due to important protocol violations.

All efficacy endpoints will be evaluated on the FAS; the co-primary endpoints will also be evaluated on the PPS.

Part C

The efficacy endpoints in Part C (the extended active treatment period) will be summarized for all patients who received any extended active treatment study drug in that part.

10.3.2. Safety Analysis Set

For Part A and Part B, the study part-specific safety analysis set (SAF) includes all randomized patients who received any study drug; it is based on the treatment received (as treated). Treatment compliance/administration and all clinical safety variables will be analyzed using the SAF.

For safety analyses of the Part C extended active treatment period, only a subset of SAF will be included, which is defined as those patients who received at least 1 dose of Part C study drug.

10.3.3. Pharmacokinetic Analysis Sets

The PK analysis set includes all treated patients who received any study drug and who had at least 1 non-missing result following the first dose of study drug.

10.3.4. Anti-Drug Antibody Analysis Sets

The ADA analysis set includes all treated patients who received any study drug and who had at least 1 non-missing ADA result following the first dose of study drug.

10.4. Statistical Methods

For continuous variables, descriptive statistics will include the following information: the number of patients reflected in the calculation (n), mean, median, SD, minimum, first and third quartiles, and maximum.

CONFIDENTIAL

VV-RIM-00127522-1.0 Approved - 05 Nov 2020 GMT-5:00

For categorical or ordinal data, frequencies and percentages will be displayed for each category.

10.4.1. Patient Disposition

The following will be provided:

- The total number of screened patients: met the inclusion criteria regarding the target indication and signed the ICF
- The total number of randomized patients: received a randomization number
- The total number of patients in each analysis set (eg, FAS, provided in Section 10.3)
- The total number of patients who discontinued the study, and the reasons for discontinuation
- The total number of patients who discontinued study treatment, and the reasons for discontinuation
- The total number of patients who took rescue medications/procedures
- A listing of patients treated but not randomized, patients randomized but not treated, and patients randomized but not treated as randomized
- A listing of patients prematurely discontinued from study treatment, along with reasons for discontinuation

10.4.2. Demography and Baseline Characteristics

Demographic and baseline characteristics will be summarized descriptively by treatment group, and by all patients combined.

10.4.3. Efficacy Analyses

10.4.3.1. Primary Efficacy Analysis

The co-primary endpoint of proportion of patients achieving a histologic response of esophageal intraepithelial eosinophil count of ≤6 eos/hpf at week 24 visit will be analyzed using the Cochran-Mantel-Haenszel (CMH) test to assess the difference in the proportion of responders in the FAS adjusting for the randomization stratification factors. In anticipation of sparse responders in the placebo group, the Fisher's exact test will be performed as a supportive analysis to confirm the results from the CMH test.

The co-primary endpoint of absolute change from baseline in the DSQ total score at week 24 will be analyzed using an analysis of covariance (ANCOVA) model for the FAS with treatment group, randomization stratification factor, and relevant baseline measurement as covariates included in the model. The cumulative proportion of responders over the entire range of possible cut-off points will be graphed to present the between-treatment-group differences at any responder level.

The intercurrent events, strategies, and the corresponding missing data handling approaches for the primary estimands of interest for the co-primary endpoints are provided in Table 4.

VV-RIM-00127522-1.0 Approved - 05 Nov 2020 GMT-5:00

Table 4: Summary of Primary Estimand for Co-primary Endpoints

Co-Primary Endpoint	Intercurrent event(s)	Strategy	Missing data handling method
Proportion of patients achieving a histologic response of esophageal intraepithelial eosinophil count of ≤6 eos/hpf at week 24 visit	Initiation of treatment with systemic and/or swallowed topical corticosteroids drugs for EoE (rescue treatment)	Composite strategy: Patients will be considered as non-responders after such events.	(1) Missing data due to COVID-19 will be imputed by multiple imputation (MI).<br>(2) Missing data due to reasons not related to COVID-19 will be imputed as non-responder.
	Initiation or change of treatment with systemic corticosteroid drugs for conditions other than EoE (prohibited medications)	Treatment policy strategy: Data collected after the patient received treatment with systemic corticosteroid drugs for conditions other than EoE will be included in the analyses.	
	Treatment discontinuation	Treatment policy strategy: Data collected after the patient discontinued treatment will be included in the analyses.	
Absolute change from baseline in the DSQ total score at week 24	Initiation of treatment with systemic and/or swallowed topical corticosteroids drugs for EoE (rescue treatment)	Hypothetical strategy: Data will be assigned using MI after such events.	Missing data will be imputed by multiple imputation (MI).
	Initiation or change of treatment with systemic corticosteroid drugs for conditions other than EoE (prohibited medications)	Treatment policy strategy: Data collected after the patient received treatment with systemic corticosteroid drugs for conditions other than EoE conditions will be included in the analyses.	
	Treatment discontinuation	Treatment policy strategy: Data collected after the patient discontinued treatment will be included in the analyses.	

To account for use of rescue treatment in the primary analysis, for peak esophageal intraepithelial eosinophil count of ≤6 eos/hpf, patients will be considered as non-responders after the use of rescue treatment. For absolute change from baseline in the DSQ total score, data will be imputed using multiple imputation (MI) for all time points subsequent to the use of rescue treatment.

If a patient has missing value for the histological response (peak esophageal intraepithelial eosinophil count of ≤6 eos/hpf) at week 24 visit due to reasons not related to COVID-19, the patient will be classified as a non-responder at week 24. Multiple imputation (MI) will be used for missing histological response due to COVID-19.

If a patient has missing value for the DSQ total score at week 24, their missing data will be imputed by multiple imputation (MI) based on similar patients who remained in the trial with observed values relevant to analysis. Similarity is based on randomized treatment group, baseline value, stratification factors, and available post-baseline values. To account for the uncertainty in the imputation, missing data at week 24 will be imputed 50 times to generate 50 complete datasets by using the MI procedure in Statistical Analysis Software (SAS).

MI will follow the 2 steps below using a random seed number of 6681774 in both steps:

- Step 1: Use the Markov Chain Monte Carlo (MCMC) method to fill in intermittent missing values (ie, those missing values followed by observed values at subsequent visits) so that a monotone missing pattern will be formed.
- Step 2: Using the datasets from step 1, missing data through week 24 will be imputed using the regression method with treatment group, randomization stratification, relevant baseline measurement, and post-baseline measurement up to week 24 included in the regression model.

Once imputations are made, the week 24 data of each of the complete datasets will be analyzed using ANCOVA. The results from the 50 analyses on the complete datasets will be combined to generate a valid overall statistical inference according to Rubin's formula (Rubin, 1987) using the SAS MIANALYZE procedure.

Sensitivity analyses will assess alternative methods to impute missing data under the assumption of missing at random or missing not at random. For the esophageal-intraepithelial-eosinophil responder analysis, alternative methods may include utilizing peak esophageal intraepithelial eosinophil count values from imputations to determine responder status. For the analysis of change from baseline in the DSQ total score, alternative methods may include mixed-effect model for repeated measures (MMRM), last observation carried forward (LOCF), and tipping point analysis. Sensitivity analyses will also include an analysis without setting data post-rescue to missing. Sensitivity analyses may include but are not limited to the methods specified above. Details will be specified in the SAP.

Handling of missing data or delayed procedures due to COVID-19 and any additional analyses required to investigate the impact of COVID-19 to understand estimated treatment effect will be detailed in the SAP.

10.4.3.2. Secondary Efficacy Analysis

10.4.3.2.1. Analysis of Secondary Efficacy Endpoints in Part A and Part B

Binary endpoints:

Secondary efficacy endpoints that measure binary responses at week 24 will be analyzed in the same fashion as the co-primary endpoint of histologic response of peak esophageal intraepithelial

VV-RIM-00127522-1.0 Approved - 05 Nov 2020 GMT-5:00

eosinophil count of ≤6 eos/hpf, including the method to handle missing data and planned sensitivity analyses.

Continuous endpoints:

For Part A enrolled patients, EoE-EREFS assessed by investigators will be used in the analysis (no centralized readings available). For Part B enrolled patients, the primary analysis of EoE-EREFS will be based on centralized readings, if available. If the centralized reading is not available, the EoE-EREFS performed by the investigator will be used for analysis.

Continuous secondary efficacy endpoints at week 24 will be analyzed using ANCOVA in a similar fashion to the co-primary endpoint of change from baseline in the DSQ total score.

For continuous efficacy data that are scheduled to be measured repeatedly post-baseline up to week 24 (eg, percent change in DSQ score from baseline to week 24), missing data will be imputed by MI as described in the primary efficacy analysis of the DSQ co-primary endpoint. Sensitivity analyses similar to those specified for the DSQ co-primary endpoint will be conducted.

For continuous efficacy data that are to be measured only once post-baseline up to week 24 (eg, percent change in peak eos/hpf from baseline to week 24), missing values at week 24 will be imputed with patient's baseline value or the available post-baseline value, whichever is worse, ie, a worst observation carried forward (WOCF) approach. MI approach is not suitable for handling missing data in this case due to the fact that the complete case patients will not have measurements available at the time points when measurements are obtained for ET patients (designed to be at their ET visits) to enable the imputation of week 24 missing values for early terminated patients. A sensitivity analysis, using the LOCF approach to impute missing values at week 24, will be performed.

To account for use of rescue treatment, data will be imputed by MI for continuous endpoints repeatedly measured or by WOCF for continuous endpoints measured only once up to week 24 visit after the use of rescue treatment.

For the four key secondary endpoints, the primary estimand and details about the intercurrent events strategy and missing data handling are presented in Table 5.

Table 5: Summary of Primary Estimand for Key Secondary Endpoints

Key Secondary Endpoint	Intercurrent event(s)	Strategy	Missing data handling method
• Absolute change in EoE-EREFS from baseline to week 24<br>• Percent change in peak esophageal intraepithelial eosinophil count (eos/hpf) from baseline to week 24<br>• Absolute change in EoE Grade Score from the EoEHSS from baseline to week 24	Initiation of treatment with systemic and/or swallowed topical corticosteroids drugs for EoE (rescue treatment)	Composite strategy: data after rescue treatment will be assigned by the worst possible value	Missing values at week 24 will be imputed with WOCF approach including baseline.
	Initiation or change of treatment with systemic corticosteroid drugs for conditions other than EoE (prohibited medications)	Treatment policy strategy: Data collected after the patient received treatment with systemic corticosteroid drugs for conditions other than	

Key Secondary Endpoint	Intercurrent event(s)	Strategy	Missing data handling method
• Absolute change in EoE Stage Score from the EoEHSS from baseline to week 24		EoE will be included in the analyses.	
	Treatment discontinuation	Treatment policy strategy: Data collected after the patient discontinued treatment will be included in the analyses.	

Sensitivity analyses will also include an analysis without setting data post-rescue to missing.

Subgroup analysis (eg, by age group) will also be performed and details will be specified in SAP.

For transcriptome endpoints, the Wilcoxon rank-sum test will be used to test if the difference in median NES of the relative change from baseline to week 24 between the dupilumab and placebo groups is statistically significant. P-values will be reported.

10.4.3.2.2. Analysis of Secondary Efficacy Endpoints in Part C

Efficacy variables measured in Part C will use the last non-missing value prior to the first dose of extended active treatment study drug as their baseline assessments. These efficacy variables will be summarized with descriptive statistics by received treatment in Part C. Inferential statistics will only be conducted as needed.

10.4.3.3. Multiplicity Considerations

Part A and Part B will be carried out as 2 separate sub-studies with no overlap in patients. Therefore, each study part will have separate and independent 2-sided alpha level of 0.05. In the event that sample size of Part B is changed based on observed data from Part A, there will be no alpha adjustment as data from Part A are considered external information to Part B.

To demonstrate efficacy for a dupilumab dose regimen, both of the individual co-primary endpoints need to be statistically significant at the 2-sided 0.05 level for that dose regimen.

Part A: Statistical significance of both co-primary efficacy endpoints will be required before drawing inferential conclusions about any secondary efficacy endpoints. The hierarchical order of secondary endpoints will be specified in Part A SAP.

Part B: Type I error rate will be controlled using a hierarchical testing procedure. The co-primary endpoints will be tested for the comparison of dupilumab QW dose regimen to placebo first and if both endpoints are statistically significant, the testing will proceed to the co-primary endpoints of dupilumab Q2W, or the secondary endpoints of dupilumab QW, with the exact order to be specified in Part B SAP.

As Part C data will be summarized using descriptive statistics, there is no multiplicity issue.

10.4.4. Safety Analysis

Safety analysis will be based on the SAF. This includes reported treatment-emergent adverse events (TEAEs) and other safety data (ie, clinical laboratory evaluations, vital signs, and 12-lead ECG results). A descriptive summary of safety results will be presented by treatment group for each study part. The use of rescue treatment will be summarized.

Any additional analyses and methods required to investigate the impact of COVID-19, including extended dosing, on the safety evaluation will be specified in the SAP.

10.4.4.1. Adverse Events

Definitions

For AEs, 2 observation periods are defined:

- The pre-treatment period is defined as the time from signing the ICF to before the first dose of study drug.
- The treatment-emergent period is defined as the day from the first dose of study treatment through the end of study including the follow-up period. The treatment-emergent period includes the placebo-controlled treatment period plus the extended active treatment period (52 weeks in total) and the 12-week follow-up period.
  - Treatment period: date of the first dose of study drug to the end of the 52-week treatment period (placebo-controlled plus extended-active treatment)
  - Follow-up period: day after the end of the treatment period to the end of study.

Treatment-emergent adverse events are defined as those that are not present at baseline or represent the exacerbation of a preexisting condition during the treatment-emergent period.

Analysis

All AEs reported in this study will be coded using the currently available version of the Medical Dictionary for Regulatory Activities (MedDRA®). Coding will be to lowest level terms. The verbatim text, the preferred term (PT), and the primary system organ class (SOC) will be listed.

Summaries of the TEAEs will include the following for each study part:

- The number (n) and percentage (%) of patients with at least 1 TEAE by SOC and PT
- TEAEs by severity (according to the grading scale outlined in Section 9.5.1), presented by SOC and PT
- TEAEs by relationship to treatment (related, not related), presented by SOC and PT
- Treatment-emergent AESIs (defined with a PT or a pre-specified grouping)

Deaths and other SAEs will be listed and summarized. The summary will include the number (n) and percentage (%) of patients with at least 1 SAE as well as the number of SAEs.

Treatment-emergent adverse events leading to permanent treatment discontinuation will be listed and summarized.

VV-RIM-00127522-1.0 Approved - 05 Nov 2020 GMT-5:00

TEAEs that occurred in Part A or Part B will be summarized by the treatment patient received in the respective part, and also for combined dupilumab QW and Q2W treatment groups.

TEAEs that occurred in Part C will be summarized by treatment received in Part C.

10.4.4.2. Other Safety

Vital Signs

Vital signs (temperature, pulse, blood pressure, and respiratory rate) will be summarized by baseline and change from baseline to each scheduled assessment time with descriptive statistics.

Number and percentage of patients with a treatment-emergent potentially clinically significant value (PCSV) will be summarized for each vital sign variable. The criteria for treatment-emergent PCSV will be defined in the SAP.

Laboratory Tests

Laboratory test results will be summarized by baseline and change from baseline to each scheduled assessment time with descriptive statistics.

Number and percentage of patients with a treatment-emergent PCSV will be summarized for each clinical laboratory test. The criteria for treatment-emergent PCSV will be defined in the SAP.

Shift tables based on baseline normal/abnormal and other tabular and graphical methods may be used to present the results for laboratory tests of interest.

Listings will be provided with flags indicating the out-of-range laboratory values.

10.4.4.3. Treatment Exposure

The duration of exposure during each study part will be calculated as follows:

- (date of last dose of study drug in the specific study part – date of first dose of study drug in the specific study part) + 7 days

In addition, duration of cumulative exposure to dupilumab across study parts will be calculated for patients who participate in both the double-blind treatment period (Part A or Part B) and the extended active treatment period as follows:

- (date of last dose of study drug – date of first dose of dupilumab in the study) + 7 days.

Number and percentage of patients exposed to study drug within a study part and across study parts will be summarized by time periods of specific lengths for each treatment group. The lengths of interest will be specified in the SAP.

In addition, duration of exposure within a study part and across study parts will be summarized as a continuous variable for each treatment group with the number of patients reflected in the calculation (n), mean, median, SD, minimum, the first and third quartiles, and maximum.

A summary of the number of study-part-specific doses for each treatment group will be provided.

VV-RIM-00127522-1.0 Approved - 05 Nov 2020 GMT-5:00

10.4.4.4. Treatment Compliance

The compliance with protocol-defined study treatment will be calculated for each of the 3 study parts separately as follows:

$$\text{Treatment compliance} = \frac{\textit{number of administered doses of study drug}}{\textit{number of planned doses of study drug}} \times 100\%$$

The treatment compliance will be summarized descriptively by specific ranges for each treatment group. The ranges of interest will be specified in the SAP.

10.4.5. Pharmacokinetics

10.4.5.1. Analysis of Drug Concentration Data

For this study, sampling will be sparse and the types of analyses will be descriptive statistics at each sampling time. No formal statistical analysis will be performed.

10.4.6. Analysis of Anti-Drug Antibody Data

ADA data will be summarized using descriptive statistics by treatment group. Samples that are positive in the ADA assay will be further characterized for the presence of neutralizing antibody.

Plots of drug concentrations will be examined and the influence of ADAs on individual PK profiles may be evaluated. Where appropriate, assessment of impact of ADA on safety and efficacy will be provided.

10.4.7. Analysis of Pharmacodynamic and Exploratory Biomarker Data

Biomarker results will be summarized by baseline, measured values, change from baseline, and percent change from baseline to each scheduled assessment time point with descriptive statistics.

10.4.8. Analysis of Quality of Life Data

Quality of life data will be analyzed using the same method as specified for the continuous secondary efficacy variables.

10.4.9. Timing of Statistical Analysis

Multiple steps of analyses are planned for this study.

- The first database lock will occur after the last Part A patient has completed their end of Part A visit including patients who have terminated early in Part A.
- The second database lock will occur after the last Part A patient has completed their week 52 visit of Part C including Part A patients who have terminated early in Part A or Part C.
- The third database lock will occur after the last Part B patient has completed their end of Part B visit including patients who have terminated early in Part B.
- After the last patient completes the last visit in Part C, analysis of all week 52 efficacy and safety endpoints may be performed.

VV-RIM-00127522-1.0 Approved - 05 Nov 2020 GMT-5:00

- The last database lock will occur after the last patient completes their last end of study visit. The end-of-study analysis will include data through the end of the 12-week follow-up period.

10.5. Additional Statistical Data Handling Conventions

The following analysis and data conventions will be followed:

Definition of baseline:

- The baseline assessment for each study part will be the latest, valid predose assessment available for the corresponding study part, respectively

General rules for handling missing data:

- If the start date of an AE or concomitant medication is incomplete or missing, it will be assumed to have occurred on or after the intake of study medication, except if an incomplete date (eg, month and year) clearly indicates that the event started prior to treatment. If the partial date indicates the same month or year of the intake of study medication date, then the start date by the study medication intake date will be imputed; otherwise, the missing day or month by the first day or the first month will be imputed.
- No imputations for missing laboratory data, ECG data, vital sign data, or physical examination data will be made.

Unscheduled assessments:

- Extra assessments (laboratory data or vital signs associated with non-protocol clinical visits or obtained in the course of investigating or managing AEs) will be included in listings and summaries. If more than 1 laboratory value is available for a given visit, the first observation will be used in summaries and all observations will be presented in listings.

10.6. Statistical Considerations Surrounding the Premature Termination of a Study

If the study is terminated prematurely for other reasons, only those parameters required for the development program and/or reporting to regulatory authorities will be summarized. Investigator and sponsor responsibilities surrounding the premature termination of a study are presented in Section 16.1.

11. DATA MANAGEMENT AND ELECTRONIC SYSTEMS

11.1. Data Management

A data management plan specifying all relevant aspects of data processing for the study (including data validation [quality-checking], cleaning, correcting, releasing) will be maintained and stored at Regeneron (Sponsor).

A medical coding plan (included in the data management plan) will specify the processes and the dictionary used for coding. All data coding (eg, AEs, baseline findings, medication, medical history/surgical history/EoE comorbidity history, etc.) will be done using internationally recognized and accepted dictionaries.

The CRF data for this study will be collected with an electronic data capture (EDC) system *Medidata Rave*.

11.2. Electronic Systems

Electronic systems that may be used to process and/or collect data in this study will include the following:

- IVRS/IWRS system – randomization and study drug supply
- EDC system – data capture/uploading
- Electronic patient diary – data capture/uploading
- Central ECG reading center-digital images
- nQuery Advisor – sample size calculations
- Statistical Analysis System (SAS) – statistical review and analysis
- Pharmacovigilance and Risk Management (PVRM) safety system
- Digital archive system for endoscopic photographic and video images

12. STUDY MONITORING

12.1. Monitoring of Study Sites

The study monitor and/or designee (eg, CRO monitor) will visit each site prior to enrollment of the first patient, and periodically during the study.

The investigator must allow study-related monitoring.

The study monitors will perform ongoing source data review to verify that data recorded in the CRF by authorized site personnel are accurate, complete, and verifiable from source documents, that the safety and rights of patients are being protected, and that the study is being conducted in accordance with the current approved protocol version and any other study agreements, ICH GCP, and all applicable regulatory requirements.

12.2. Source Document Requirements

Investigators are required to prepare and maintain adequate and accurate patient records (source documents).

The investigator must keep all source documents on file with the CRF (throughout this protocol, CRF refers to either a paper CRF or an electronic CRF). Case report forms and source documents must be available at all times for inspection by authorized representatives of the sponsor and regulatory authorities.

CONFIDENTIAL

VV-RIM-00127522-1.0 Approved - 05 Nov 2020 GMT-5:00

12.3. Case Report Form Requirements

Study data obtained in the course of the clinical study will be recorded on eCRFs within the EDC system by trained site personnel. All required CRFs must be completed for each and every patient enrolled in the study. After review of the clinical data for each patient, the investigator must provide an electronic signature. A copy of each patient CRF casebook is to be retained by the investigator as part of the study record and must be available at all times for inspection by authorized representatives of the sponsor and regulatory authorities.

Corrections to the CRF will be entered in the CRF by the investigator or an authorized designee. All changes, including date and person performing corrections, will be available via the audit trail, which is part of the EDC system. For corrections made via data queries, a reason for any alteration must be provided.

13. AUDITS AND INSPECTIONS

This study may be subject to a quality assurance audit or inspection by the sponsor or regulatory authorities. Should this occur, the investigator is responsible for:

- Informing the sponsor of a planned inspection by the authorities as soon as notification is received, and authorizing the sponsor's participation in the inspection
- Providing access to all necessary facilities, study data, and documents for the inspection or audit
- Communicating any information arising from inspection by the regulatory authorities to the sponsor immediately
- Taking all appropriate measures requested by the sponsor to resolve the problems found during the audit or inspection

Documents subject to audit or inspection include but are not limited to all source documents, CRFs, medical records, correspondence, ICFs, IRB/EC files, documentation of certification and quality control of supporting laboratories, and records relevant to the study maintained in any supporting pharmacy facilities. Conditions of study material storage are also subject to inspection. In addition, representatives of the sponsor may observe the conduct of any aspect of the clinical study or its supporting activities both within and outside of the investigator's institution.

In all instances, the confidentiality of the data must be respected.

14. ETHICAL AND REGULATORY CONSIDERATIONS

14.1. Good Clinical Practice Statement

It is the responsibility of both the sponsor and the investigators to ensure that this clinical study will be conducted in accordance with the ethical principles that have their origin in the Declaration of Helsinki, and that are consistent with the ICH guidelines for GCP and applicable regulatory requirements.

VV-RIM-00127522-1.0 Approved - 05 Nov 2020 GMT-5:00

14.2. Informed Consent

The principles of informed consent are described in ICH guidelines for GCP.

The ICF used by the investigator must be reviewed and approved by the sponsor prior to submission to the appropriate IRB/EC. A copy of the IRB- or EC-approved ICF and documentation of approval must be provided to the sponsor before study drug will be shipped to the study site.

It is the responsibility of the investigator or designee (if acceptable by local regulations) to obtain written informed consent from each adult patient, and written informed consent or assent from each adolescent patient and written informed consent from his/her parent(s) or legal guardian(s), prior to patient's participation in the study and after the aims, methods, objectives, and potential hazards of the study have been explained to the patient and adolescent patient's parent(s) or legal guardian(s) in language that he/she can understand. The ICF should be signed and dated by the adult patient, and the ICF/IAF by adolescent patient and his/her parent(s) or legal guardian(s), and by the investigator or authorized designee who reviewed the ICF/IAF with the patient/patient's parent(s) or legal guardian(s).

For adolescent patients, local law must be observed in deciding whether the consent of 1 or both parents/guardians is required. If only 1 parent or guardian signs the consent form, the investigator must document the reason the other parent or guardian did not sign. The patient may also be required to sign and date the ICF, as determined by the IRB or EC and in accordance with the local regulations and requirements.

- Patients/parents/guardians who can write but cannot read will have the ICF/IAF read to them before signing and dating the ICF/IAF.
- Patients who can understand but who can neither write nor read will have the ICF/IAF read to them in presence of an impartial witness, who will sign and date the ICF/IAF to confirm that informed consent/assent was given.

The original ICF/IAF must be retained by the investigator as part of the patient's study record, and a copy of the signed ICF must be given to the adult patient, or to the adolescent patient and his/her parent(s) or legal guardian(s).

If new safety information results in significant changes in the risk/benefit assessment, the ICF/IAF must be reviewed and updated appropriately. All study patients (and adolescent patients' parent[s] or legal guardian[s]) must be informed of the new information and provide their written consent/assent if they wish to continue in the study. The original signed revised ICF/IAF must be maintained in the patient's study record and a copy must be given to the adult patient, and the adolescent patient and his/her parent(s) or legal guardian(s).

14.3. Patients Confidentiality and Data Protection

The investigator must take all appropriate measures to ensure that the anonymity of each study patient will be maintained. Patients should be identified by a patient identification number only, on CRFs or other documents submitted to the sponsor. Documents that will not be submitted to the sponsor (eg, signed ICF/IAF) must be kept in strict confidence.

CONFIDENTIAL

VV-RIM-00127522-1.0 Approved - 05 Nov 2020 GMT-5:00

The patient's and investigator's personal data, which may be included in the sponsor database, will be treated in compliance with all applicable laws and regulations. The sponsor shall take all appropriate measures to safeguard and prevent access to this data by any unauthorized third party.

14.4. Institutional Review Board/Ethics Committee

An appropriately constituted IRB or EC, as described in ICH guidelines for GCP, must review and approve:

- The protocol, ICF/IAF, and any other materials to be provided to the patients (eg, advertising) before any patient may be enrolled in the study
- Any amendment or modification to the study protocol or ICF before implementation, unless the change is necessary to eliminate an immediate hazard to the patient, in which case the IRB or EC should be informed as soon as possible
- Ongoing studies on an annual basis or at intervals appropriate to the degree of risk

In addition, the IRB or EC should be informed of any event likely to affect the safety of patients or the continued conduct of the clinical study.

A copy of the IRB or EC approval letter with a current list of the IRB or EC members and their functions must be received by the sponsor prior to shipment of drug supplies to the investigator. The approval letter should include the study number and title, the documents reviewed, and the date of the review.

Records of the IRB or EC review and approval of all study documents (including approval of ongoing studies) must be kept on file by the investigator.

15. PROTOCOL AMENDMENTS

The sponsor may not implement a change in the design of the protocol or ICF without an IRB- or EC-approved amendment. All substantial protocol amendments will be approved by the competent authorities before changes are implemented according to national regulations.

16. PREMATURE TERMINATION OF THE STUDY OR CLOSE-OUT OF A SITE

16.1. Premature Termination of the Study

The sponsor has the right to terminate the study prematurely. Reasons may include efficacy, safety, or futility, among others. Should the sponsor decide to terminate the study, the investigator(s) will be notified in writing.

VV-RIM-00127522-1.0 Approved - 05 Nov 2020 GMT-5:00

16.2. Close-out of a Site

The sponsor and the investigator have the right to close-out a site prematurely.

Investigator's Decision

The investigator must notify the sponsor of a desire to close-out a site in writing, providing at least 30 days' notice. The final decision should be made through mutual agreement with the sponsor. Both parties will arrange the close-out procedures after review and consultation.

Sponsor's Decision

The sponsor will notify the investigator(s) of a decision to close-out a study site in writing. Reasons may include the following, among others:

- The investigator has received all items and information necessary to perform the study, but has not enrolled any patient within a reasonable period of time
- The investigator has violated any fundamental obligation in the study agreement, including but not limited to, breach of this protocol (and any applicable amendments), breach of the applicable laws and regulations, or breach of any applicable ICH guidelines
- The total number of patients required for the study are enrolled earlier than expected

In all cases, the appropriate IRB or EC and Health Authorities must be informed according to applicable regulatory requirements, and adequate consideration must be given to the protection of the patients' interests.

17. STUDY DOCUMENTATION

17.1. Certification of Accuracy of Data

A declaration assuring the accuracy and content of the data recorded on the eCRF must be signed electronically by the investigator. This signed declaration accompanies each set of patient final eCRF that will be provided to the sponsor.

17.2. Retention of Records

The investigator must retain all essential study documents, including ICFs/IAFs, source documents, investigator copies of CRFs, and drug accountability records for at least 15 years following the completion or discontinuation of the study, or longer, if a longer period is required by relevant regulatory authorities. The investigator must obtain written approval from the sponsor before discarding or destroying any essential study documents during the retention period following study completion or discontinuation. Records must be destroyed in a manner that ensures confidentiality.

If the investigator's personal situation is such that archiving can no longer be ensured, the investigator must inform the sponsor (written notification) and the relevant records will be transferred to a mutually agreed-upon destination.

VV-RIM-00127522-1.0 Approved - 05 Nov 2020 GMT-5:00

18. DATA QUALITY ASSURANCE

In accordance with ICH E6, the sponsor is responsible for quality assurance to ensure that the study is conducted and the data generated, recorded, and reported in compliance with the protocol, GCP, and any applicable regulatory requirement(s). The planned quality assurance and quality control procedures for the study are summarized.

Data Management

The sponsor is responsible for the data management of this study including quality checking of the data (Section 11.1).

Study Monitoring

The investigator must allow study-related monitoring, IRB/EC review, audits, and inspections from relevant health regulatory authorities, and provide direct access to source data documents (Section 12.1, Section 12.2, and Section 13)

The study monitors will perform ongoing source data review to verify that data recorded in the CRF by authorized site personnel are accurate, complete, and verifiable from source documents, that the safety and rights of patients are being protected, and that the study is being conducted in accordance with the current approved protocol version and any other study agreements, ICH GCP, and all applicable regulatory requirements (Section 12.1).

All subject/patient data collected during the study will be recorded on paper or electronic CRF unless the data are transmitted to the sponsor or designee electronically (eg, laboratory data). The investigator is responsible for affirming that data entries in the CRF are accurate and correct by electronically signing a declaration that accompanies each set of patient/subject final CRF (Section 12.3 and Section 17.1).

Study Documentation

The investigator must maintain accurate documentation (source data) that supports the information entered in the CRF (Section 12.2).

The investigator will retain all records and documents, including signed ICFs, pertaining to the conduct of this study for at least 15 years after study completion, unless local regulations or institutional policies require a longer retention period. No records may be destroyed during the retention period without the written approval of the sponsor. No records may be transferred to another location or party without written notification to the sponsor (Section 17.2).

19. CONFIDENTIALITY

Confidentiality of information is provided as a separate agreement.

20. FINANCING AND INSURANCE

Financing and insurance information is provided as a separate agreement.

VV-RIM-00127522-1.0 Approved - 05 Nov 2020 GMT-5:00

21. PUBLICATION POLICY

Publication rights and procedures will be outlined in a separate clinical study agreement.

22. REFERENCES

Abonia JP, Blanchard C, Butz BB, Rainey HF, Collins MH, Stringer K, et al. Involvement of mast cells in eosinophilic esophagitis. The Journal of Allergy and Clinical Immunology. 2010 Jul;126(1):140-9. PubMed PMID: 20538331. Pubmed Central PMCID: 2902643.

Abonia JP, Rothenberg ME. Eosinophilic esophagitis: rapidly advancing insights. Annual Review of Medicine. 2012;63:421-34. PubMed PMID: 22034864.

Ally MR, Maydonovitch CL, Betteridge JD, Veerappan GR, Moawad FJ. Prevalence of eosinophilic esophagitis in a United States military health-care population. Dis Esophagus. 2015 Aug-Sep;28(6):505-11. PubMed PMID: 24827543.

Arias Á1, Pérez-Martínez I2, Tenías JM3, Lucendo AJ4. Systematic review with meta-analysis: the incidence and prevalence of eosinophilic oesophagitis in children and adults in population-based studies. Aliment Pharmacol Ther. 2016 Jan;43(1):3-15. doi: 10.1111/apt.13441. Epub 2015 Oct 28.

Arora AA, Weiler CR, Katzka DA. Eosinophilic esophagitis: allergic contribution, testing, and management. Current Gastroenterology Reports. 2012 Jun;14(3):206-15. PubMed PMID: 22422505.

Assa'ad A. Eosinophilic esophagitis: association with allergic disorders. Gastrointestinal Endoscopy Clinics of North America. 2008 Jan;18(1):119-32; x. PubMed PMID: 18061106.

Barbie, DA, P Tamayo, JS Boehm, SY Kim, S E Moody, IF Dunn, AC Schinzel, P Sandy, E Meylan, C Scholl, S Frohling, EM Chan, ML Sos, K Michel, C Mermel, SJ Silver, BA Weir, JH Reiling, Q Sheng, PB Gupta, RC Wadlow, H Le, S Hoersch, BS Wittner, S Ramaswamy, DM Livingston, DM Sabatini, MMeyerson, RK Thomas, ES Lander, JP Mesirov, DE Root, DG Gilliland, T Jacks, and WC Hahn. Systematic Rna Interference Reveals That Oncogenic Kras-Driven Cancers Require Tbk1. Nature 462, no 7269. 2009 5 Nov: 108-12. PMID: 19847166

Blanchard C, Rothenberg ME. Basic pathogenesis of eosinophilic esophagitis. Gastrointestinal Endoscopy Clinics of North America. 2008 Jan;18(1):133-43; x. PubMed PMID: 18061107. Pubmed Central PMCID: 2194642.

Blanchard C, Stucke EM, Burwinkel K, Caldwell JM, Collins MH, Ahrens A, et al. Coordinate interaction between IL-13 and epithelial differentiation cluster genes in eosinophilic esophagitis. Journal of Immunology. 2010 Apr 1;184(7):4033-41. PubMed PMID: 20208004. Pubmed Central PMCID: 3807813.

Blanchard C, Wang N, Stringer KF, Mishra A, Fulkerson PC, Abonia JP, et al. Eotaxin-3 and a uniquely conserved gene-expression profile in eosinophilic esophagitis. The Journal of Clinical Investigation. 2006 Feb;116(2):536-47. PubMed PMID: 16453027. Pubmed Central PMCID: 1359059.

Blanchard C, Mingler MK, Vicario M, Abonia JP, Wu YY, Lu TX, et al. IL-13 involvement in eosinophilic esophagitis: transcriptome analysis and reversibility with glucocorticoids. The

VV-RIM-00127522-1.0 Approved - 05 Nov 2020 GMT-5:00

Journal of Allergy and Clinical Immunology. 2007 Dec;120(6):1292-300. PubMed PMID: 18073124.

Brooks R. EuroQol: the current state of play, Health Policy. 1996 Jul;37(1):53-72.

Bullock JZ, Villanueva JM, Blanchard C, Filipovich AH, Putnam PE, Collins MH, et al. Interplay of adaptive th2 immunity with eotaxin-3/c-C chemokine receptor 3 in eosinophilic esophagitis. Journal of Pediatric Gastroenterology and Nutrition. 2007 Jul;45(1):22-31. PubMed PMID: 17592361.

Charman CR, Venn AJ, et al. The patient-oriented eczema measure. Arch Dermatol 2004; 140:1513-1519.

Cianferoni A, Spergel J. Eosinophilic Esophagitis: A Comprehensive Review. Clin Rev Allergy Immunol. 2016 Apr;50(2):159-74. PubMed PMID: 26194940

Clayton F, Fang JC, Gleich GJ, Lucendo AJ, Olalla JM, Vinson LA, Lowichik A, Chen X, Emerson L, Cox K, O'Gorman MA, Peterson KA. Eosinophilic esophagitis in adults is associated with IgG4 and not mediated by IgE. Gastroenterology. 2014 Sep;147(3):602-9. doi: 10.1053/j.gastro.2014.05.036. Epub 2014 Jun 4.

DeBrosse CW, Franciosi JP, King EC, Butz BK, Greenberg AB, Collins MH, et al. Long-term outcomes in pediatric-onset esophageal eosinophilia. The Journal of Allergy and Clinical Immunology. 2011 Jul;128(1):132-8. PubMed PMID: 21636117. Pubmed Central PMCID: 3130990.

Dellon ES. Epidemiology of eosinophilic esophagitis. Gastroenterology Clinics of North America. 2014a Jun;43(2):201-18. PubMed PMID: 24813510. Pubmed Central PMCID: 4019938.

Dellon ES, Aderoju A, Woosley JT, Sandler RS, Shaheen NJ. Variability in diagnostic criteria for eosinophilic esophagitis: a systematic review. The American Journal of Gastroenterology. 2007 Oct;102(10):2300-13. PubMed PMID: 17617209.

Dellon ES, Jensen ET, Martin CF, Shaheen NJ, Kappelman MD. Prevalence of eosinophilic esophagitis in the United States. Clinical Gastroenterology and Hepatology : the official clinical practice journal of the American Gastroenterological Association. 2014b Apr;12(4):589-96 e1. PubMed PMID: 24035773. Pubmed Central PMCID: 3952040.

Dellon ES, Irani AM, Hill MR, Hirano I. Development and field testing of a novel patient-reported outcome measure of dysphagia in patients with eosinophilic esophagitis. Aliment Pharmacol Ther. 2013;38(6):634-642.

Dellon ES, Katzka DA, Collins MH, Hamdani M, Gupta SK, Hirano I; MP-101-06 Investigators. Budesonide Oral Suspension Improves Symptomatic, Endoscopic, and Histologic Parameters Compared With Placebo in Patients With Eosinophilic Esophagitis. Gastroenterology. 2017 Mar;152(4):776-786.e5. doi: 10.1053/j.gastro.2016.11.021. Epub 2016 Nov 23. PMID: 27889574

Eluri S, Runge TM, Hansen J, Kochar B, Reed CC, Robey BS, Woosley JT, Shaheen NJ, Dellon ES. Diminishing Effectiveness of Long-Term Maintenance Topical Steroid Therapy in PPI Non-Responsive Eosinophilic Esophagitis. Clin Transl Gastroenterol. 2017 Jun 15;8(6):e97. doi: 10.1038/ctg.2017.27. PMID: 28617448

EMA. Assessment Report – Jorveza. 09 November 2017, EMA/774645/2017

VV-RIM-00127522-1.0 Approved - 05 Nov 2020 GMT-5:00

Falk GW. Clinical presentation of eosinophilic esophagitis in adults. Gastroenterology Clinics of North America. 2014 Jun;43(2):231-42. PubMed PMID: 24813512.

Furuta GT, Aceves SS, The National Biome Initiative: An Allergy Perspective. J Allergy Clin Immunol. 2017 Apr;139(4):1131-1134.

Hamilton JD, Suárez-Fariñas M, Dhingra N, Cardinale I, Li X, Kostic A, Ming JE, Radin AR, Krueger JG, Graham N, Yancopoulos GD, Pirozzi G, Guttman-Yassky E. Dupilumab improves the molecular signature in skin of patients with moderate-to-severe atopic dermatitis. J Allergy Clin Immunol. 2014 Dec;134(6):1293-1300. doi: 10.1016/j.jaci.2014.10.013.

Hirano I, Aceves SS. Clinical implications and pathogenesis of esophageal remodeling in eosinophilic esophagitis. Gastroenterology Clinics of North America. 2014 Jun;43(2):297-316. PubMed PMID: 24813517.

Hirano I, Moy N, Heckman MG, Thomas CS, Gonsalves N, Achem SR. Endoscopic assessment of the oesophageal features of eosinophilic oesophagitis: validation of a novel classification and grading system. Gut. 2013 Apr;62(4):489-95. PubMed PMID: 22619364.

Hirano D, Oka S, Tanaka S, Sumimoto K, Ninomiya Y, Tamaru Y, Shigita K, Hayashi N, Urabe Y, Kitadai Y, Shimamoto F, Arihiro K, Chayama K. Clinicopathologic and endoscopic features of early-stage colorectal serrated adenocarcinoma. BMC Gastroenterol. 2017 Dec 12;17(1):158. doi: 10.1186/s12876-017-0702-x. PMID: 29233113

Howell MD, Kim BE, Gao P, Grant AV, Boguniewicz M, Debenedetto A, et al. Cytokine modulation of atopic dermatitis filaggrin skin expression. The Journal of Allergy and Clinical Immunology. 2007 Jul;120(1):150-5. PubMed PMID: 17512043. Pubmed Central PMCID: 2669594.

Hruz P, Straumann A, Bussmann C, Heer P, Simon HU, Zwahlen M, Beglinger C, Schoepfer AM; Swiss EoE study group. Escalating incidence of eosinophilic esophagitis: a 20-year prospective, population-based study in Olten County, Switzerland. J Allergy Clin Immunol. 2011 Dec;128(6):1349-1350.e5. PubMed PMID: 22019091

Hudgens S1, Evans C2, Phillips E3, Hill M3. Psychometric validation of the Dysphagia Symptom Questionnaire in patients with eosinophilic esophagitis treated with budesonide oral suspension. J Patient Rep Outcomes. 2017;1(1):3. doi: 10.1186/s41687-017-0006-5. Epub 2017 Sep 12.

Kapel RC, Miller JK, Torres C, Aksoy S, Lash R, Katzka DA. Eosinophilic esophagitis: a prevalent disease in the United States that affects all age groups. Gastroenterology. 2008 May;134(5):1316-21. PubMed PMID: 18471509.

Kwiatek MA, Hirano I, Kahrilas PJ, Rothe J, Luger D, Pandolfino JE. Mechanical properties of the esophagus in eosinophilic esophagitis. Gastroenterology. 2011 Jan;140(1):82-90. doi: 10.1053/j.gastro.2010.09.037. Epub 2010 Sep 19.

Liacouras CA, Furuta GT, Hirano I, Atkins D, Attwood SE, Bonis PA, et al. Eosinophilic esophagitis: updated consensus recommendations for children and adults. The Journal of Allergy and Clinical Immunology. 2011 Jul;128(1):3-20 e6; quiz 1-2. PubMed PMID: 21477849.

VV-RIM-00127522-1.0 Approved - 05 Nov 2020 GMT-5:00

Lucendo AJ,2, Molina-Infante J, Arias Á, et al, Guidelines on eosinophilic esophagitis: evidence-based statements and recommendations for diagnosis and management in children and adults. United European Gastroenterol J. 2017 Apr;5(3):335-358

Mishra A. Mechanism of eosinophilic esophagitis. Immunology and Allergy Clinics of North America. 2009 Feb;29(1):29-40, viii. PubMed PMID: 19141339. Pubmed Central PMCID: 2650237.

Mishra A, Rothenberg ME. Intratracheal IL-13 induces eosinophilic esophagitis by an IL-5, eotaxin-1, and STAT6-dependent mechanism. Gastroenterology. 2003 Nov;125(5):1419-27. PubMed PMID: 14598258.

Moawad FJ, Veerappan GR, Lake JM, Maydonovitch CL, Haymore BR, Kosisky SE, et al. Correlation between eosinophilic oesophagitis and aeroallergens. Alimentary Pharmacology & Therapeutics. 2010 Feb 15;31(4):509-15. PubMed PMID: 19925501.

Nicodème F, Hirano I, Chen J, Robinson K, Lin Z, Xiao Y, Gonsalves N, Kwasny MJ, Kahrilas PJ, Pandolfino JE. Esophageal distensibility as a measure of disease severity in patients with eosinophilic esophagitis. Clin Gastroenterol Hepatol. 2013 Sep;11(9):1101-1107.e1. doi: 10.1016/j.cgh.2013.03.020. Epub 2013 Apr 13.

Prasad GA, Alexander JA, Schleck CD, Zinsmeister AR, Smyrk TC, Elias RM, Locke GR 3rd, Talley NJ. Epidemiology of eosinophilic esophagitis over three decades in Olmsted County, Minnesota. Clin Gastroenterol Hepatol. 2009 Oct;7(10):1055-61. doi: 10.1016/j.cgh.2009.06.023. Epub 2009 Jul 1.

Rabin R, Gudex C, Selai C, Herdman M. From translation to version management: a history and review of methods for the cultural adaptation of the EuroQol five-dimensional questionnaire, Value Health 2014 Jan;17(1):70-76.

Roy-Ghanta S, Larosa DF, Katzka DA. Atopic characteristics of adult patients with eosinophilic esophagitis. Clin Gastroenterol Hepatol. 2008 May;6(5):531-5. doi: 10.1016/j.cgh.2007.12.045. Epub 2008 Mar 4.

Rubin, D.B. (1987), Multiple Imputation for Nonresponse in Surveys, New York: John Wiley & Sons, Inc.

Schoepfer AM, Straumann A, Panczak R, et al. Development and Validation of a Symptom-Based Activity Index for Adults with Eosinophilic Esophagitis. Gastroenterology. 2014;147(6):1255-1266.

Schoepfer A. Development, validation and evaluation of an adult and pediatric Eosinophilic Esophagitis Activity Index (EEsAI). ClinicalTrials.gov. Last updated 2 September 2013. Available at:
http://clinicaltrials.gov/ct2/show/NCT00939263?term=eosinophilic+esophagitis&recr=Open&rank=19. Accessed 27 February 2014.

Sherrill JD, Kiran KC, Blanchard C, Stucke EM, Kemme KA, Collins MH, et al. Analysis and expansion of the eosinophilic esophagitis transcriptome by RNA sequencing. Genes Immun 2014; 15(6):361-9.

VV-RIM-00127522-1.0 Approved - 05 Nov 2020 GMT-5:00

Singla MB1, Moawad FJ1. An Overview of the Diagnosis and Management of Eosinophilic Esophagitis. Clin Transl Gastroenterol. 2016 Mar 17;7:e155. doi: 10.1038/ctg.2016.4.

Spergel JM, Brown-Whitehorn TF, Beausoleil JL, Franciosi J, Shuker M, Verma R, et al. 14 years of eosinophilic esophagitis: clinical features and prognosis. Journal of Pediatric Gastroenterology and Nutrition. 2009 Jan;48(1):30-6. PubMed PMID: 19172120.

Straumann A. The natural history and complications of eosinophilic esophagitis. Gastrointestinal Endoscopy Clinics of North America. 2008 Jan;18(1):99-118; ix. PubMed PMID: 18061105.

Straumann A, Bauer M, Fischer B, Blaser K, Simon HU. Idiopathic eosinophilic esophagitis is associated with a T(H)2-type allergic inflammatory response. The Journal of Allergy and Clinical Immunology. 2001 Dec;108(6):954-61. PubMed PMID: 11742273.

Straumann A, Spichtin HP, Grize L, Bucher KA, Beglinger C, Simon HU. Natural history of primary eosinophilic esophagitis: a follow-up of 30 adult patients for up to 11.5 years. Gastroenterology. 2003 Dec;125(6):1660-9. PubMed PMID: 14724818.

Subramanian, A, P Tamayo, VK Mootha, S Mukherjee, BL Ebert, MA Gillette, A Paulovich, SL Pomeroy, TR Golub, ES Lander, and JP Mesirov. Gene Set Enrichment Analysis: A Knowledge-Based Approach for Interpreting Genome-Wide Expression Profiles. Proc Natl Acad Sci U S A 102, no. 43. 2005 25 Oct: 15545-50. PMID: 16199517

Veerappan GR, Perry JL, Duncan TJ, Baker TP, Maydonovitch C, Lake JM, Wong RK, Osgard EM. Prevalence of eosinophilic esophagitis in an adult population undergoing upper endoscopy: a prospective study. Clin Gastroenterol Hepatol. 2009 Apr;7(4):420-6, 426.e1-2. PubMed PMID: 19162236.

Weinbrand-Goichberg J, Segal I, Ovadia A, Levine A, Dalal I. Eosinophilic esophagitis: an immune-mediated esophageal disease. Immunologic Research. 2013 Jul;56(2-3):249-60. PubMed PMID: 23579771.

Wen T, Stucke EM, Grotjan TM, Kemme KA, Abonia JP, Putnam PE, Franciosi JP, Garza JM, Kaul A, King EC, Collins MH, Kushner JP, Rothenberg ME. Molecular diagnosis of eosinophilic esophagitis by gene expression profiling. Gastroenterology. 2013 Dec;145(6):1289-99. PubMed PMID: 23978633

Wright BL, Kulis M, Guo R, Orgel KA, Wolf WA, Burks AW, Vickery BP, Dellon ES. Food-specific IgG4 is associated with eosinophilic esophagitis. J Allergy Clin Immunol. 2016 Oct;138(4):1190-1192.e3. doi: 10.1016/j.jaci.2016.02.024. Epub 2016 Apr 6.

CONFIDENTIAL

VV-RIM-00127522-1.0 Approved - 05 Nov 2020 GMT-5:00

23. INVESTIGATOR'S AGREEMENT

I have read the attached protocol: A PHASE 3, RANDOMIZED, 3-PART STUDY TO INVESTIGATE THE EFFICACY AND SAFETY OF DUPILUMAB IN ADULT AND ADOLESCENT PATIENTS WITH EOSINOPHILIC ESOPHAGITIS, and agree to abide by all provisions set forth therein.

I agree to comply with the current International Council for Harmonisation Guideline for Good Clinical Practice and the laws, rules, regulations, and guidelines of the community, country, state, or locality relating to the conduct of the clinical study.

I also agree that persons debarred from conducting or working on clinical studies by any court or regulatory agency will not be allowed to conduct or work on studies for the sponsor or a partnership in which the sponsor is involved. I will immediately disclose it in writing to the sponsor if any person who is involved in the study is debarred, or if any proceeding for debarment is pending, or, to the best of my knowledge, threatened.

This document contains confidential information of the sponsor, which must not be disclosed to anyone other than the recipient study staff and members of the Institutional Review Board or Ethics Committee. I agree to ensure that this information will not be used for any purpose other than the evaluation or conduct of the clinical investigation without the prior written consent of the sponsor.

______________________________ ____________________

(Signature of Investigator) (Date)

____

(Printed Name)

VV-RIM-00127522-1.0 Approved - 05 Nov 2020 GMT-5:00

SIGNATURE OF SPONSOR'S RESPONSIBLE OFFICERS

(Medical/Study Director, Regulatory Representative, Clinical Study Team Lead, and Biostatistician)

To the best of my knowledge, this protocol accurately describes the conduct of the study.

Study Title:	A Phase 3, Randomized, 3-Part Study to Investigate the Efficacy and Safety of Dupilumab in Adult and Adolescent Patients with Eosinophilic Esophagitis
Protocol Number:	R668-EE-1774
Protocol Version:	R668-EE-1774 Amendment 5

See appended electronic signature page

Sponsor's Responsible Medical/Study Director

See appended electronic signature page

Sponsor's Responsible Regulatory Liaison

See appended electronic signature page

Sponsor's Responsible Clinical Study Team Lead

See appended electronic signature page

Sponsor's Responsible Biostatistician

Signature Page for VV-RIM-00127522 v1.0

ESig Approval	[redacted]
ESig Approval	[redacted]
ESig Approval	[redacted]
ESig Approval	[redacted]

Signature Page for VV-RIM-00127522 v1.0 Approved
