# Supplementary material for: Dupilumab Improves Histopathologic Features in Patients With Eosinophilic Esophagitis: LIBERTY EoE TREET Study Results
Source: Gastro Hep Adv. 2025 Feb 24;4(6):100646. doi: 10.1016/j.gastha.2025.100646 (PMC12143628; doi:10.1016/j.gastha.2025.100646)
Supplement: Supplementary Materials [file mmc4.docx]

- Why was this study done?

Eosinophilic esophagitis (EoE) is an immune-mediated disease of the esophagus in which eosinophils (a type of white blood cell involved in the defense against allergens) accumulate in the lining of the esophagus. Patients with EoE have difficulties swallowing food (dysphagia) which can cause poor nutrition, emotional distress, and reduced social activity. EoE diagnosis is partially established by finding an abnormally high number of eosinophils in esophageal biopsies. However, this does not always correlate with symptoms patients might experience. In this study, researchers examined microscopic changes in the cells of the esophagus and assessed whether these measurements could be used to monitor more accurately patients’ response to treatment.

- What did the researchers do?

The LIBERTY EoE TREET study is a clinical trial where researchers assessed the effect of dupilumab 300 mg weekly against placebo in adolescents and adults with EoE. Biopsies were taken when the study started, and after 24 and 52 weeks of treatment. Cellular changes were assessed microscopically and scored using the EoE histology scoring system (EoEHSS).

- What did the researchers find?

Dupilumab treatment for 24 weeks led to greater improvements in several microscopic features of the disease compared with placebo. These improvements were maintained after 52 weeks.

- What do the findings mean?

These results show that dupilumab improved microscopic disease features of EoE assessed using the EoEHSS. This scoring system provides a more comprehensive assessment of cellular changes in the esophagus than eosinophil counts only and may better guide patient care.
